# Supplementary figures and images for: White matter lesions and DTI metrics related to various types of dysfunction in cerebral palsy: A meta-analysis and systematic review
Source: PLoS One. 2025 Jan 24;20(1):e0312378. doi: 10.1371/journal.pone.0312378 (PMC11760009; doi:10.1371/journal.pone.0312378)

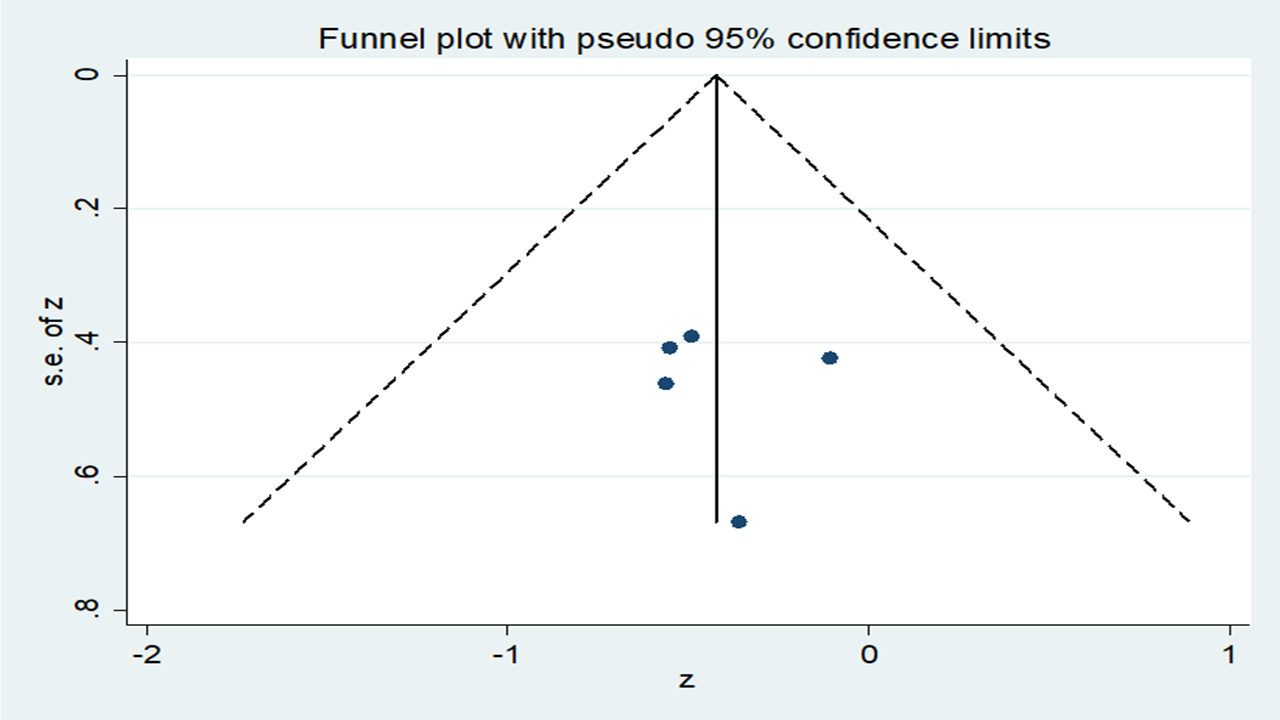

Supplement: S1 Fig — (TIF) [file pone.0312378.s001.TIF]

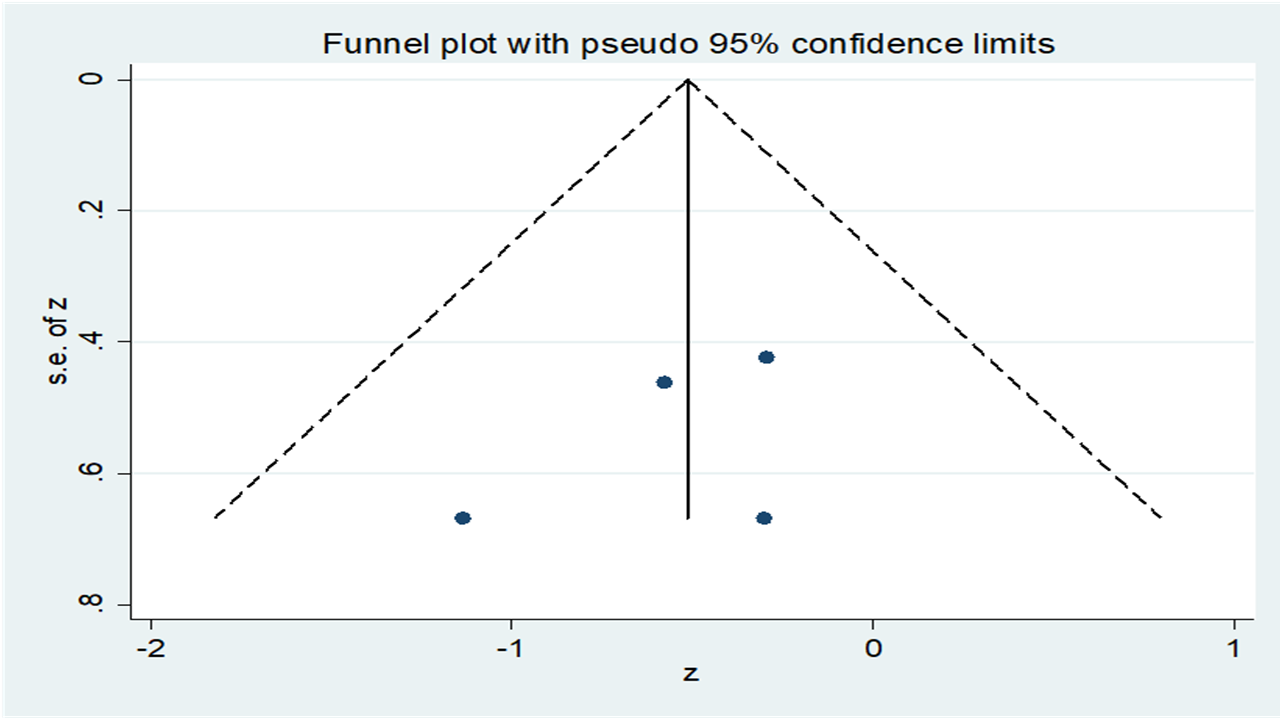

Supplement: S2 Fig — (TIF) [file pone.0312378.s002.TIF]

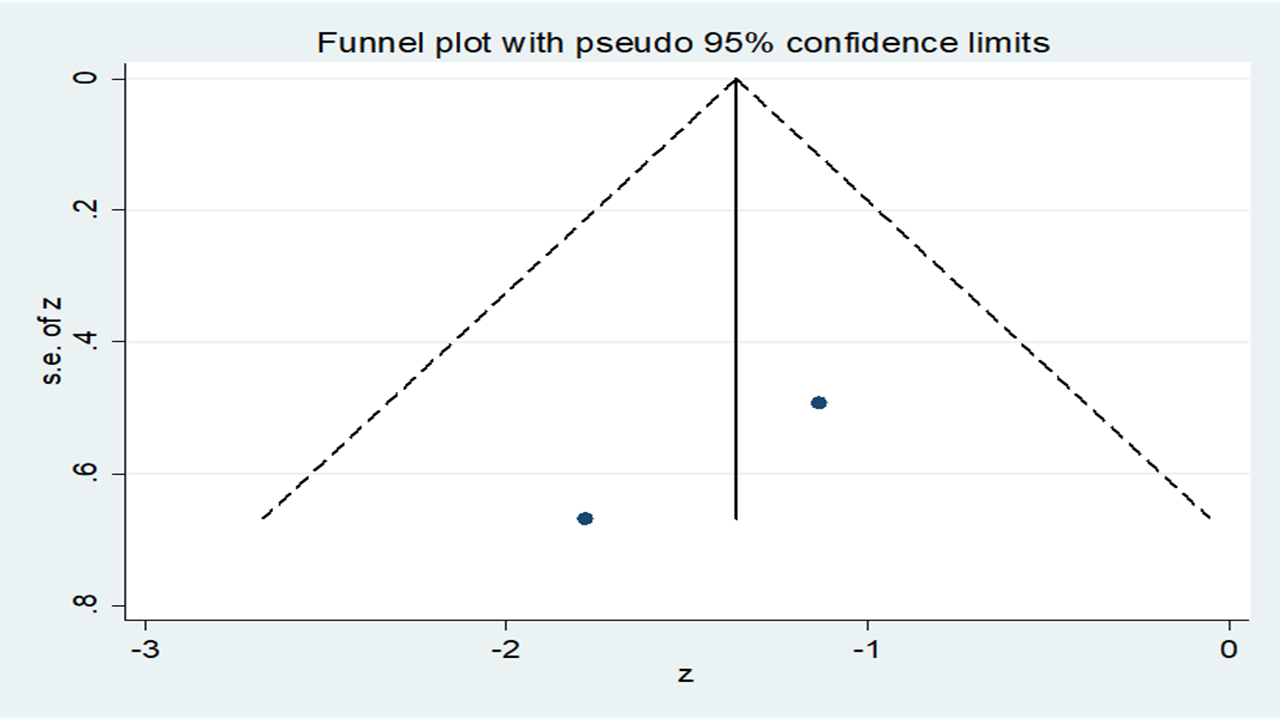

Supplement: S3 Fig — (TIF) [file pone.0312378.s003.TIF]

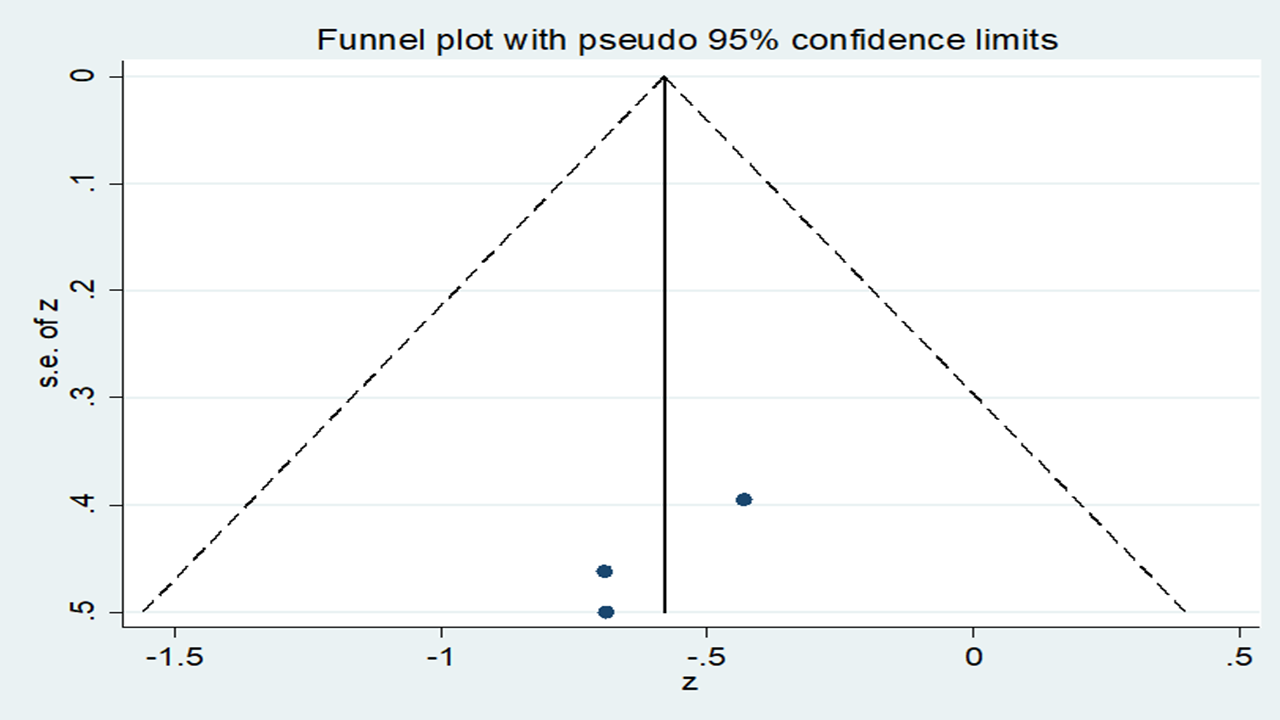

Supplement: S4 Fig — (TIF) [file pone.0312378.s004.TIF]

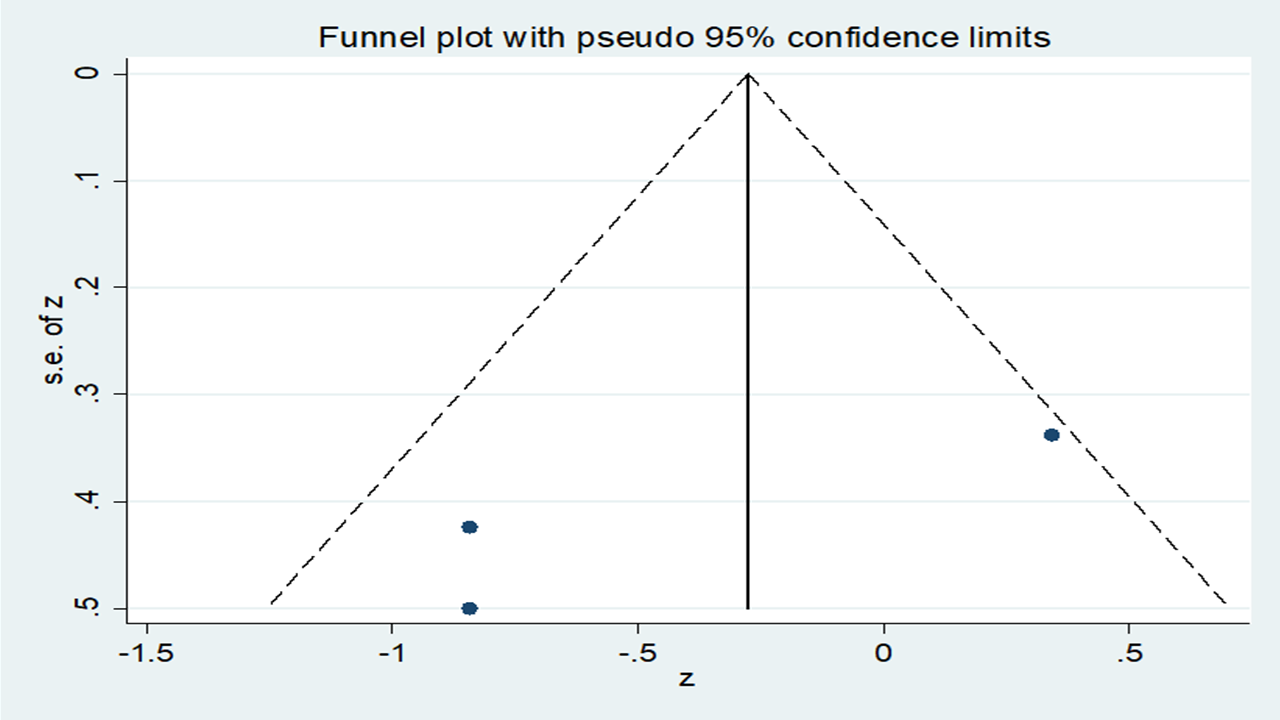

Supplement: S5 Fig — (TIF) [file pone.0312378.s005.TIF]

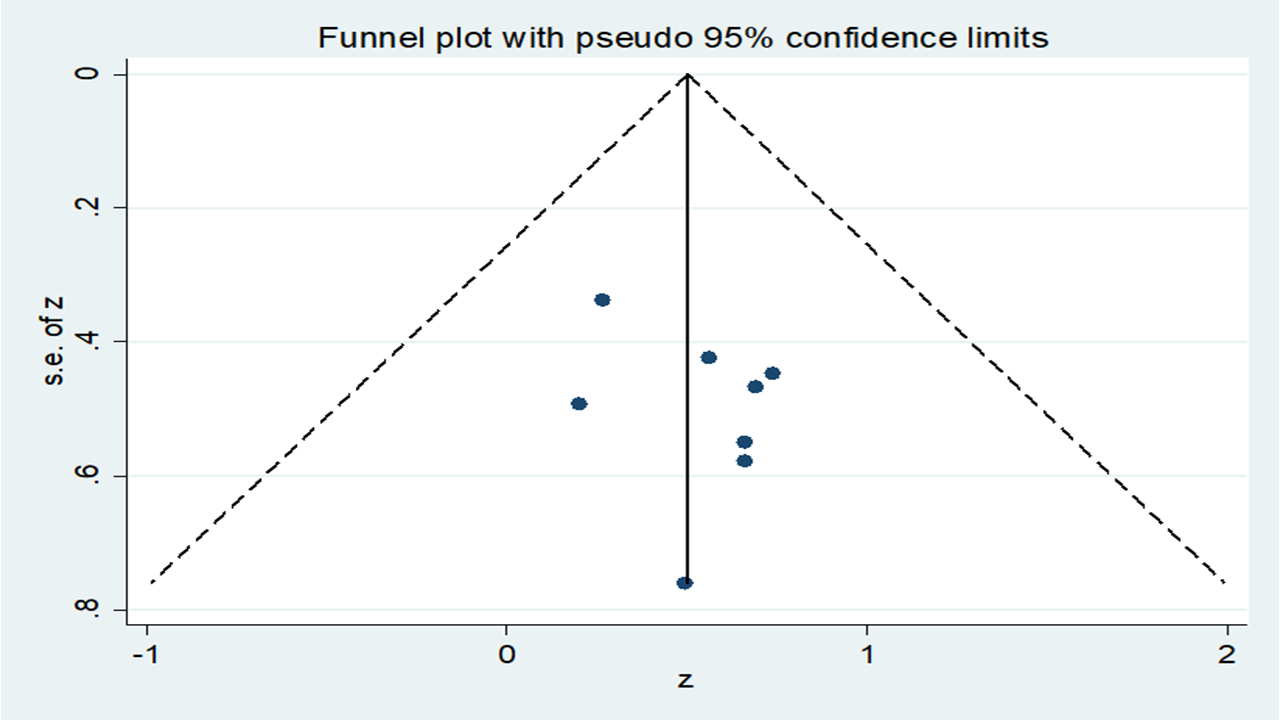

Supplement: S6 Fig — (TIF) [file pone.0312378.s006.TIF]
