# Supplementary material for: White matter lesions and DTI metrics related to various types of dysfunction in cerebral palsy: A meta-analysis and systematic review
Source: PLoS One. 2025 Jan 24;20(1):e0312378. doi: 10.1371/journal.pone.0312378 (PMC11760009; doi:10.1371/journal.pone.0312378)
Supplement: S1 Table — (DOCX) [file pone.0312378.s007.docx]

Supporting information

**TableS1.Abbreviation**

| Cerebral Palsy | CP |
| --- | --- |
| unilateral cerebral palsy | UCP |
| Magnetic Resonance Imaging | MRI |
| Diffusion Tensor Imaging | DTI |
| Diffusion Tensor Image Analysis along the Perivascular Space | DTI-ALPS |
| White Matter | WM |
| Fractional Anisotropy | FA |
| Mean Diffusivity | MD |
| Radial Diffusivity | RD |
| Average Diffusion Coefficient | ADC |
| Fiber Number | FN |
| Apparent Fiber Density | AFD |
| Asymmetry Index | AI |
| Anterior Cingulate Cortex | ACC |
| Corticospinal Tract | CST |
| Posterior Limb of the Internal Capsule | PLIC |
| Posterior Thalamic Radiation | PTR |
| Corpus Callosum | CC |
| Optic Radiation | OR |
| Superior Longitudinal Fasciculus | SLF |
| Periventricular Leukomalacia | PVL |
| Parietal White Matter | PWM |
| Frontal White Matter | FWM |
| Occipital White Matter | OWM |
| Temporal White Matter | TWM |
| Melbourne assess-ment 2 | MA2 |
| Gross Motor Function Classification System | GMFCS |
| Gross Motor Function Measure | GMFM |
| Box and Block Test | BBT |
| Assisting Hand Assessment | AHA |
| Manual Ability Classification System | MACS |
| Jebsen-Taylor Hand Function Test | JTHFT |
| Children's Hand-use Experience Questionnaire | CHEQ |
| Selective Control Assessment of the Lower Extremity | SCALE |
| Time Up and Go Test | TUG |
| Dysphagia Disorder Survey | DDS |
| Peabody Picture Vocabulary Test | PPVT |
| Wisconsin Card Sorting Test | WCST |
| Global Pain Scale | GPS |
| three-dimensional instrumented gait analysis | 3DGA |
| 10 Meter Walk Test | 10MWT |
| Functional Level of Hemiplegia | FxL |
| Revisie Amsterdamse Kinder Intelligentie Test | RAKIT |

|  |
| --- |
|  |
|  |
|  |
|  |
