# Supplementary material for: White matter lesions and DTI metrics related to various types of dysfunction in cerebral palsy: A meta-analysis and systematic review
Source: PLoS One. 2025 Jan 24;20(1):e0312378. doi: 10.1371/journal.pone.0312378 (PMC11760009; doi:10.1371/journal.pone.0312378)
Supplement: S2 Table — (DOCX) [file pone.0312378.s008.docx]

Supporting information

**TableS2 the NOS Evaluation Score Form of All Studies**

| **Study** | **Selection** | | | | **Comparability** | **Outcome** | | | **Total score** |
| --- | --- | --- | --- | --- | --- | --- | --- | --- | --- |
|  | Representativeness of the exposed cohort | Selection of the non-exposed cohort | Ascertainment of exposure | The outcome of interest was not present at the start of the study | Comparability of cohorts based on the design or analysis | Assessment of outcome | Effcient follow-up duration | Adequacy of follow up |  |
| Araneda, R.21019 | 1 | 1 | 1 | 1 | 1 | 1 | 0 | 0 | 6 |
| Galli, J.2018 | 1 | 1 | 1 | 0 | 1 | 1 | 1 | 0 | 6 |
| Ballester-Plané, J.2017 | 1 | 1 | 1 | 1 | 1 | 1 | 1 | 0 | 7 |
| Hoon, A. H., Jr.2009 | 1 | 1 | 1 | 1 | 2 | 1 | 1 | 1 | 9 |
| Scheck, S. M.2015 | 1 | 1 | 1 | 1 | 2 | 1 | 1 | 1 | 9 |
| Mourão, L. F.2017 | 1 | 1 | 1 | 1 | 1 | 1 | 1 | 1 | 8 |
| Laporta-Hoyos, O.2017 | 1 | 1 | 1 | 1 | 2 | 1 | 1 | 1 | 9 |
| Rai, Y.2013 | 1 | 1 | 1 | 1 | 1 | 1 | 0 | 0 | 6 |
| Laporta-Hoyos, O.2023 | 1 | 1 | 0 | 1 | 1 | 1 | 1 | 1 | 7 |
| Jeroen Vermeulen, R.2011 | 1 | 1 | 1 | 1 | 0 | 1 | 1 | 0 | 6 |
| Rha, D. W.2012 | 1 | 1 | 1 | 1 | 1 | 1 | 1 | 1 | 8 |
| Trivedi, R.2010 | 1 | 1 | 1 | 1 | 2 | 1 | 1 | 1 | 9 |
| Arrigoni, F.2016 | 1 | 1 | 1 | 1 | 2 | 1 | 1 | 1 | 9 |
| Arrigoni, F.2015 | 1 | 1 | 1 | 0 | 2 | 1 | 1 | 1 | 8 |
| Vuong, A.2021 | 1 | 1 | 1 | 1 | 1 | 1 | 1 | 0 | 7 |
| Lee, J. D.2011 | 1 | 1 | 1 | 1 | 1 | 1 | 1 | 1 | 8 |
| Yoshida, S.2010 | 1 | 1 | 1 | 1 | 2 | 1 | 1 | 1 | 9 |
| Van Gestel, L.2013 | 1 | 1 | 1 | 1 | 2 | 1 | 1 | 1 | 9 |
| Meyns, P.2016 | 1 | 1 | 0 | 0 | 1 | 1 | 1 | 1 | 6 |
| Jaatela, J.2023 | 1 | 1 | 1 | 1 | 2 | 1 | 1 | 1 | 9 |
| Azizi, S.2021 | 1 | 1 | 1 | 1 | 1 | 1 | 1 | 1 | 8 |
| Jaatela, J.2023 | 1 | 1 | 1 | 1 | 2 | 1 | 1 | 1 | 9 |
| Mackey, A.2011 | 1 | 1 | 1 | 1 | 2 | 1 | 1 | 1 | 9 |
| Ferre, C.2018 | 1 | 1 | 1 | 0 | 1 | 1 | 1 | 1 | 7 |
| Kim, J. H.2015 | 1 | 1 | 1 | 1 | 0 | 1 | 1 | 1 | 6 |
| Pannek, K.2014 | 1 | 1 | 1 | 1 | 2 | 1 | 1 | 1 | 9 |
| Tsao, H.2015 | 1 | 1 | 1 | 1 | 2 | 1 | 1 | 1 | 9 |
| Kuczynski, A. M.2018 | 1 | 1 | 1 | 1 | 1 | 1 | 1 | 1 | 8 |
| Mailleux, L.2020 | 1 | 1 | 1 | 1 | 2 | 1 | 1 | 1 | 9 |
| Simona Fiori2015 | 1 | 1 | 1 | 1 | 2 | 1 | 1 | 1 | 9 |
| Kuo, H. C.2017 | 1 | 1 | 1 | 1 | 0 | 1 | 1 | 1 | 7 |
| Weinstein, M.2018 | 1 | 1 | 1 | 1 | 2 | 1 | 1 | 1 | 9 |
| Holmström, L.2011 | 1 | 1 | 1 | 1 | 1 | 1 | 1 | 1 | 8 |
| Scheck, S. M.2016 | 1 | 1 | 0 | 1 | 1 | 1 | 1 | 0 | 6 |
| Yin, Y.2023 | 1 | 1 | 1 | 1 | 2 | 1 | 0 | 0 | 8 |
| Weinstein, M.2014 | 1 | 1 | 1 | 1 | 2 | 1 | 1 | 1 | 9 |
| Kim, H. S.2022 | 1 | 0 | 1 | 0 | 2 | 1 | 1 | 1 | 7 |
| Hung, Y. C.2019 | 1 | 1 | 1 | 0 | 1 | 1 | 1 | 0 | 8 |
| Hasegawa 2018 | 1 | 1 | 1 | 1 | 2 | 1 | 0 | 0 | 7 |
| Jiang2019 | 1 | 1 | 1 | 1 | 0 | 1 | 1 | 0 | 6 |
| Madhavan 2014 | 1 | 1 | 1 | 1 | 2 | 1 | 1 | 1 | 9 |
| Wang 2014 | 1 | 1 | 1 | 1 | 2 | 1 | 1 | 1 | 9 |
| Hodge2017 | 1 | 1 | 1 | 1 | 1 | 1 | 1 | 0 | 7 |
| Reid2016 | 1 | 1 | 1 | 1 | 2 | 1 | 1 | 1 | 9 |
| Schertz 2016 | 1 | 1 | 1 | 1 | 2 | 1 | 1 | 1 | 9 |
| Weinstein 2015 | 1 | 1 | 1 | 1 | 2 | 1 | 1 | 1 | 9 |
