# Supplementary material for: White matter lesions and DTI metrics related to various types of dysfunction in cerebral palsy: A meta-analysis and systematic review
Source: PLoS One. 2025 Jan 24;20(1):e0312378. doi: 10.1371/journal.pone.0312378 (PMC11760009; doi:10.1371/journal.pone.0312378)
Supplement: S3 Table — (DOCX) [file pone.0312378.s009.docx]

Supporting information

**Table 3. Characteristics of the Included Studies.**

| Study | Year | Country | Sample size | Age at MRI（year） | sensorimotor pathways | DTI metrics | Scales | r | P | NOS score |
| --- | --- | --- | --- | --- | --- | --- | --- | --- | --- | --- |
| Araneda, R. | 2019 | Belgium | 40 | —— | OR |  | field of view |  |  | 6 |
| Galli, J. | 2018 | Italy | 9 | 8.75± 2.58 | SLF |  | hree-dimensional block construction task |  |  | 6 |
| Ballester-Plané, J. | 2017 | Spain | 25 | 21.55±8.10 | WM |  | GMFCS |  |  | 7 |
| Hoon, A. H., Jr. | 2009 | USA | 28 | 5.83 ±2.5 | PTR | Imaging Grading | Classification of walking disorders | 0.52 | 0.003 | 9 |
|  |  |  |  |  | PTR |  | proprioception of the upper limbs | -0.69 | 0.03 |  |
|  |  |  |  |  | PTR |  | proprioception of the lower limbs | -0.67 | 0.02 |  |
| Scheck, S. M. | 2015 | Australia | 71 | （5-17） | CST | MD | Flanker task | 0.402 | 0.0412 | 9 |
|  |  |  |  |  | ACC |  |  | -0.5856 | 0.0045 |  |
| Mourão, L. F. | 2017 | USA | 20 | （5.91-17.5） | CC | FA | Dysphagia Disorder Survey (DDS) | -0.667 | 0.013 | 8 |
|  |  |  |  |  |  | RD |  | 0.594 | 0.032 |  |
| Laporta-Hoyos, O. | 2017 | Spain | 33 | 24.42±12.61 | WM |  | Wisconsin card sorting test (WCST) |  |  | 9 |
| Rai, Y. | 2013 | India | 22 | 7.72±2.6 | WM |  | Revisie Amsterdamse Kinder Intelligentie Test （RAKIT） | | | 6 |
| Laporta-Hoyos, O. | 2023 | Australia | 37 | 11.8±3.2 | WM |  | PPVT-IV |  |  | 7 |
| Jeroen Vermeulen, R. | 2011 | Netherlands | 5 | （5-23） | WM |  | Computer-Based instrument for Low motor Language Test | | | 6 |
| Rha, D. W. | 2012 | South Korea | 19 | （0.7-2.9） | CST |  | GMFCS |  |  | 8 |
| Trivedi, R. | 2010 | India | 39 | 8 | motor tracts | MD | GMFCS | 0.342 | 0.004 | 9 |
|  |  |  |  |  | sensory tracts | MD |  | 0.278 | 0.023 |  |
|  |  |  |  |  | CST | FA |  | -0.48 | 0.01 |  |
| Arrigoni, F. | 2016 | Italy | 25 | 9.59±0.59 | CST | FA | GMFCS | -0.52 | 0.05 | 9 |
|  |  |  |  |  | PTR |  |  | -0.5 | 0.05 |  |
|  |  |  |  |  | CST |  | MACS | -0.5 | 0.01 |  |
|  |  |  |  |  | PTR |  |  | -0.45 | 0.02 |  |
|  |  |  |  |  | corona radiata |  |  | -0.45 | 0.02 |  |
|  |  |  |  |  | SLF |  |  | -0.4 | 0.05 |  |
| Arrigoni, F. | 2015 | Italy | 25 | 11.8 ±3.1 | WM |  | GMFCS |  |  | 8 |
| Vuong, A. | 2021 | USA | 12 | 11.5 | WM |  | GMFCS、CSALE、GMFM |  |  | 7 |
| Lee, J. D. | 2011 | South Korea | 23 | 2.2±2.0 | WM |  | GMFCS |  |  | 8 |
| Yoshida, S. | 2010 | Japan | 34 | 12.86±4.80 | CST |  | GMFCS | -0.1 | 0.05 | 9 |
|  |  |  |  |  | PLIC |  |  | -0.57 | 0.01 |  |
|  |  |  |  |  | PTR |  |  | -0.38 | 0.05 |  |
| Van Gestel, L. | 2013 | Belgium | 25 | (3-12) | CST | FA | modified Ashworth | 0.32 |  | 9 |
|  |  |  |  |  |  | ADC |  | 0.31 |  |  |
|  |  |  |  |  | CST |  | Global Pain Scale (GPS) | 0.3 |  |  |
| Meyns, P. | 2016 | Belgium | 59 | (3.58-12.33) | CST、CC、LV |  | 3D Instrumented Gait Analysis (3DGA) |  |  | 6 |
| Jaatela, J. | 2023 | Finland | 50 | (10-18) | CC |  | Hi-End Foot-scan system |  |  | 9 |
| Azizi, S. | 2021 | Iran | 26 | 9.03±2.73 | CST | MD | 10 Meter Walk Test (10MWT) | -0.638 | 0.014 | 8 |
|  |  |  |  |  |  | RD |  | -0.688 | 0.005 |  |
|  |  |  |  |  |  | FA |  | 0.664 | 0.01 |  |
|  |  |  |  |  | CST | MD | TUG | 0.66 | 0.01 |  |
|  |  |  |  |  |  | RD |  | 0.694 | 0.006 |  |
| Jaatela, J. | 2023 | Finland | 25 | （10.8-18.1） | CST | MD | BBT | -0.82 | 0.048 | 9 |
|  |  |  |  |  |  | AFD |  | 0.92 | 0.01 |  |
|  |  |  |  |  | CST | FA | Static standing and dynamic gait stability | -0.5 | 0.046 |  |
|  |  |  |  |  |  | AFD |  | -0.71 | 0.031 |  |
| Mackey, A. | 2011 | New Zealand | 19 | 15 | CST | FA | MA2 | -0.67 | 0.05 | 9 |
|  |  |  |  |  | CST | FA | BBT | -0.58 | 0.05 |  |
| Ferre, C. | 2018 | USA | 44 | 9.1±3.1 | CST | FA | BBT | -0.39 | 0.05 | 7 |
| Kim, J. H. | 2015 | South Korea | 40 | 13.7 ± 3.0 | CST |  | Functional Level of Hemiplegia(FxL) |  |  | 6 |
| Pannek, K. | 2014 | Australia | 80 | (5-17) | CST | FA | AHA | 0.44 | 0.05 | 9 |
| Tsao, H. | 2015 | Australia | 80 | 11.3 ± 3.3 | CST | FA | MA2 | 0.33 | 0.01 | 9 |
|  |  |  |  |  | CST | FA | AHA | 0.26 | 0.01 |  |
| Kuczynski, A. M. | 2018 | Canada | 33 |  | CST | FA | KINARM Robotics Mission | -0.39 | 0.01 | 8 |
| Mailleux, L. | 2020 | Belgium | 34 | 10.25± 2.58 | CST | MD | AHA | -0.49 |  | 9 |
|  |  |  |  |  |  | MD | MA2 | -0.51 |  |  |
|  |  |  |  |  |  | MD | JTHFT | -0.51 |  |  |
| Simona Fiori | 2015 | Italy | 36 | 12.61±3.20 | CPC | AI | AHA | -0.31 | 0.034 | 9 |
| Kuo, H. C. | 2017 | USA | 20 | 9.2±3.2 | CST |  | MACS |  |  | 7 |
| Weinstein, M. | 2018 | UK | 15 | 9.4±2.5 | CC | AD | JTTHF | 0.76 | 0.006 | 9 |
|  |  |  |  |  |  | MD |  | 0.66 | 0.03 |  |
| Holmström, L. | 2011 | Sweden | 15 | (7.2-17.0) | PLIC |  | BBT |  |  | 8 |
| Scheck, S. M. | 2016 | Australia | 46 | (5-17) | CST |  | JTHFT |  |  | 6 |
| Yin, Y. | 2023 | China | 18 | 8.37±2.47 | glymphatic system |  | MACS |  |  | 8 |
| Weinstein, M. | 2014 | Israel | 12 | 10.6±2.7 | PLIC | FA | CHEQ | 0.76 | 0.01 | 9 |
|  |  |  |  |  | CC | FA | CHEQ | 0.8 | 0.017 |  |
|  |  |  |  |  |  | MD |  | -0.75 | 0.031 |  |
|  |  |  |  |  |  | FA | JTTHF | -0.83 | 0.011 |  |
| Kim, H. S. | 2022 | South Korea | 92 | 7.03±2.56 | CST | FA | Upper Limb Length | -0.578 |  | 7 |
|  |  |  |  |  |  | MD |  | 0.512 |  |  |
| Hung, Y. C. | 2019 | USA | 39 | (6-17) | CC | AD | The drawer task | -0.33 | 0.037 | 8 |
| Hasegawa | 2018 | Japan | 25 | 0.29 | CST | FA | GMFCS | -0.33 | 0.05 | 7 |
|  |  |  |  |  | PTR | FA |  | -0.28 | 0.05 |  |
|  |  |  |  |  | CST | ADC | GMFCS | 0.48 | 0.06 |  |
| Jiang | 2019 | China | 20 | 11.7±2.1 | CST | FA | GMFCS | -0.73 | 0.01 | 6 |
|  |  |  |  |  | PLIC | FA |  | -0.8 | 0.01 |  |
|  |  |  |  |  | PTR | FA |  | -0.46 | 0.01 |  |
| Madhavan | 2014 | USA | 8 | 1 | CST | FA | GMFCS | -0.48 | 0.01 | 9 |
| Wang | 2014 | China | 46 | 1.86±0.56 | CST | FA | GMFCS | -0.42 | 0.01 | 9 |
| Hodge | 2017 | Canada | 28 | 10.3±4.6 | CST | FA | AHA | 0.61 | 0.04 | 7 |
| Reid | 2016 | Australia | 24 | 11.7±2.7 | CST | FA | AHA | 0.58 | 0.03 | 9 |
| Schertz | 2016 | Israel | 20 | 10.9±1.8 | CST | FA | AHA | 0.19 | 0.05 | 9 |
|  |  |  |  |  | PLIC |  |  | 0.64 | 0.04 |  |
| Weinstein | 2015 | Israel | 12 | 11.0±3.6 | CST | FA | AHA | 0.56 | 0.05 | 9 |
