# Supplementary material for: White matter lesions and DTI metrics related to various types of dysfunction in cerebral palsy: A meta-analysis and systematic review
Source: PLoS One. 2025 Jan 24;20(1):e0312378. doi: 10.1371/journal.pone.0312378 (PMC11760009; doi:10.1371/journal.pone.0312378)
Supplement: S4 Table — (DOCX) [file pone.0312378.s010.docx]

Supporting information

**TableS4.** **Table of All Study Identifiers**

| Identifier | Title | Yaer | Reason for Exclusion | Ultimately Included or Not |
| --- | --- | --- | --- | --- |
| Form Pubmed | | | | |
| 1 | Neuroimaging in cerebral palsy | 2004 | Title and abstract clearly do not meet inclusion criteria | No |
| 2 | Early Imaging and Adverse Neurodevelopmental Outcome in Asphyxiated Newborns Treated With Hypothermia | 2017 | Non-CP | No |
| 3 | A connectome-based approach to assess motor outcome after neonatal arterial ischemic stroke | 2021 | Non-CP | No |
| 4 | Isolated Medial Rectus Nuclear Palsy as a Rare Presentation of Midbrain Infarction | 2015 | Title and abstract clearly do not meet inclusion criteria | No |
| 5 | MR imaging and outcome of term neonates with perinatal asphyxia: value of diffusion-weighted MR imaging and ¹H MR spectroscopy | 2011 | Title and abstract clearly do not meet inclusion criteria | No |
| 6 | Transependymal Movement of Cerebrospinal Fluid in Neurological and Psychiatric Pathological Conditions | 2016 | Title and abstract clearly do not meet inclusion criteria | No |
| 7 | Giant axonal neuropathy: diffusion-weighted imaging features of the brain | 2006 | Title and abstract clearly do not meet inclusion criteria | No |
| 8 | Effects of Piano Training in Unilateral Cerebral Palsy Using Probabilistic and Deterministic Tractography: A Case Report | 2021 | Title and abstract clearly do not meet inclusion criteria | No |
| 9 | Clinical and imaging outcomes after intrathecal injection of umbilical cord tissue mesenchymal stem cells in cerebral palsy: a randomized double-blind sham-controlled clinical trial | 2021 | No correlation analysis | No |
| 10 | Clinical and Radiological Profiles of COVID-19 Patients with Neurological Symptomatology: A Comparative Study | 2021 | Title and abstract clearly do not meet inclusion criteria | No |
| 11 | Impact of early brain lesions on the optic radiations in children with cerebral palsy | 2022 | No correlation analysis | No |
| 12 | Brain lesions in preterm infants: initial diagnosis and follow-up | 2010 | review | No |
| 13 | The effects of hemorrhagic parenchymal infarction on the establishment of sensori-motor structural and functional connectivity in early infancy | 2014 | Non-CP | No |
| 14 | Whole-Brain DTI Assessment of White Matter Damage in Children with Bilateral Cerebral Palsy: Evidence of Involvement beyond the Primary Target of the Anoxic Insult | 2016 |  | Yes |
| 15 | Diffusion tensor brain imaging findings at term-equivalent age may predict neurologic abnormalities in low birth weight preterm infants | 2003 | Non-CP | No |
| 16 | Impact of anti-gravity locomotion (AlterG) training on structure and function of corticospinal tract and gait in children with cerebral palsy | 2019 | No correlation analysis | No |
| 17 | Assessment of neuroplasticity of corticospinal tract induced by antigravity treadmill (AlterG) in cerebral palsy children | 2018 | No correlation analysis | No |
| 18 | Contribution of altered corticospinal microstructure to gait impairment in children with cerebral palsy | 2021 |  | Yes |
| 19 | The impact of an anti-gravity treadmill (AlterG) training on walking capacity and corticospinal tract structure in children with cerebral palsy | 2017 | No correlation analysis | No |
| 20 | The impact of AlterG training on balance and structure of vestibulospinal tract in cerebral palsy children | 2018 | No correlation analysis | No |
| 21 | Early predictors of neurodevelopment after perinatal arterial ischemic stroke: a systematic review and meta-analysis | 2023 | review | No |
| 22 | CST recovery in pediatric hemiplegic patients: Diffusion tensor tractography study | 2013 | No correlation analysis | No |
| 23 | Susceptibility-Weighted Imaging Identifies Iron-Oxide-Labeled Human Neural Stem Cells: Automated Computational Detection | 2016 | Title and abstract clearly do not meet inclusion criteria | No |
| 24 | Whole-brain structural connectivity in dyskinetic cerebral palsy and its association with motor and cognitive function | 2017 |  | Yes |
| 25 | Phase II Randomized Controlled Trial of Constraint-Induced Movement Therapy in Multiple Sclerosis. Part 2: Effect on White Matter Integrity | 2018 | Title and abstract clearly do not meet inclusion criteria | No |
| 26 | Segmental Alterations of the Corpus Callosum in Progressive Supranuclear Palsy: A Multiparametric Magnetic Resonance Imaging Study | 2021 | Title and abstract clearly do not meet inclusion criteria | No |
| 27 | [Utility of spinal MRI tractography and spinal MRI angiography in the diagnosis of spastic diplegia] | 2022 | Title and abstract clearly do not meet inclusion criteria | No |
| 28 | Prophylactic maternal N-acetylcysteine in rats prevents maternal inflammation-induced offspring cerebral injury shown on magnetic resonance imaging | 2013 | Title and abstract clearly do not meet inclusion criteria | No |
| 29 | Quantitative diffusion tensor MRI fiber tractography of sensorimotor white matter development in premature infants | 2005 | Title and abstract clearly do not meet inclusion criteria | No |
| 30 | Agenesis of the arcuate fasciculi in congenital bilateral perisylvian syndrome: a diffusion tensor imaging and tractography study | 2010 | Title and abstract clearly do not meet inclusion criteria | No |
| 31 | Neuroimaging Perspectives of Perinatal Arterial Ischemic Stroke | 2020 | Title and abstract clearly do not meet inclusion criteria | No |
| 32 | Motor Skill Training May Restore Impaired Corticospinal Tract Fibers in Children With Cerebral Palsy | 2020 | No correlation analysis | No |
| 33 | Neuroimaging in cerebral palsy | 2004 | Title and abstract clearly do not meet inclusion criteria | No |
| 34 | A new framework for analysis of three-dimensional shape and architecture of human skeletal muscles from in vivo imaging data | 2022 | Title and abstract clearly do not meet inclusion criteria | No |
| 35 | Mitii™ ABI: study protocol of a randomised controlled trial of a web-based multi-modal training program for children and adolescents with an Acquired Brain Injury (ABI) | 2015 | Title and abstract clearly do not meet inclusion criteria | No |
| 36 | COMBIT: protocol of a randomised comparison trial of COMbined modified constraint induced movement therapy and bimanual intensive training with distributed model of standard upper limb rehabilitation in children with congenital hemiplegia | 2013 | Title and abstract clearly do not meet inclusion criteria | No |
| 37 | Diffusion Tensor Imaging Abnormalities in the Cerebral White Matter Correlate with Sex-Dependent Neurobehavioral Deficits in Adult Mice with Neonatal Ischemia | 2016 | Title and abstract clearly do not meet inclusion criteria | No |
| 38 | Parechovirus Encephalitis and Neurodevelopmental Outcomes | 2016 | Title and abstract clearly do not meet inclusion criteria | No |
| 39 | [Joubert syndrome: findings at conventional magnetic resonance image and at diffusion tensor imaging] | 2012 | Title and abstract clearly do not meet inclusion criteria | No |
| 40 | Development of the corpus callosum and cognition after neonatal encephalopathy | 2023 | Non-CP | No |
| 41 | Etiology of impaired selective motor control: emerging evidence and its implications for research and treatment in cerebral palsy | 2014 | review | No |
| 42 | Correlating Quantitative MRI-based Apparent Diffusion Coefficient Metrics with 24-month Neurodevelopmental Outcomes in Neonates from the HEAL Trial | 2023 | Title and abstract clearly do not meet inclusion criteria | No |
| 43 | Diffusion tensor magnetic resonance imaging tractography in progressive supranuclear palsy | 2011 | Title and abstract clearly do not meet inclusion criteria | No |
| 44 | H1 haplotype of the MAPT gene is associated with lower regional gray matter volume in healthy carriers | 2009 | Title and abstract clearly do not meet inclusion criteria | No |
| 45 | Electric field simulations of transcranial direct current stimulation in children with perinatal stroke | 2023 | No correlation analysis | No |
| 46 | Cognitive impairment in progressive supranuclear palsy-Richardson's syndrome is related to white matter damage | 2016 | Title and abstract clearly do not meet inclusion criteria | No |
| 47 | Regional vulnerability of longitudinal cortical association connectivity: Associated with structural network topology alterations in preterm children with cerebral palsy | 2015 | No correlation analysis | No |
| 48 | Cortical ischaemic patterns in term partial-prolonged hypoxic-ischaemic injury-the inter-arterial watershed demonstrated through atrophy | 2020 | Title and abstract clearly do not meet inclusion criteria | No |
| 49 | Early micro- and macrostructure of sensorimotor tracts and development of cerebral palsy in high risk infants | 2021 | No correlation analysis | No |
| 50 | Degenerative changes of the corticospinal tract in pediatric patients showing deteriorated motor function: A diffusion tensor tractography study | 2015 | No correlation analysis | No |
| 51 | Diffusion tensor imaging demonstrated radiologic differences between diplegic and quadriplegic cerebral palsy | 2012 | No correlation analysis | No |
| 52 | Comparative assessment of therapeutic response to physiotherapy with or without botulinum toxin injection using diffusion tensor tractography and clinical scores in term diplegic cerebral palsy children | 2013 | Title and abstract clearly do not meet inclusion criteria | No |
| 53 | Chorioamnionitis in the pathogenesis of brain injury in preterm infants | 2014 | Title and abstract clearly do not meet inclusion criteria | No |
| 54 | Study of Effect of Sympathetic Nerve on Children's Brain Diseases Based on Analysis of Magnetic Resonance Imaging Kurtosis | 2020 | Title and abstract clearly do not meet inclusion criteria | No |
| 55 | Intravenous thrombolytic therapy in patients with stroke mimics: baseline characteristics and safety profile | 2011 | Title and abstract clearly do not meet inclusion criteria | No |
| 56 | Case report and literature review: fatal cerebral fat embolism following facial autologous fat graft | 2023 | review | No |
| 57 | In utero methadone exposure permanently alters anatomical and functional connectivity: A preclinical evaluation | 2023 | Title and abstract clearly do not meet inclusion criteria | No |
| 58 | Objective and Clinically Feasible Analysis of Diffusion MRI Data can Help Predict Dystonia After Neonatal Brain Injury | 2021 | Non-CP | No |
| 59 | The clinical syndrome and etiological mechanism of infarction involving the nucleus prepositus hypoglossi | 2008 | Title and abstract clearly do not meet inclusion criteria | No |
| 60 | Diffusion tensor imaging-demonstrated differences between hemiplegic and diplegic cerebral palsy with symmetric periventricular leukomalacia | 2013 | Non-CP | No |
| 61 | Neurodevelopmental Outcomes of Neonatal Rotavirus-Associated Leukoencephalopathy | 2022 | Title and abstract clearly do not meet inclusion criteria | No |
| 62 | Inferior rectus palsy as an isolated ocular motor sign: acquired etiologies and outcome | 2013 | Title and abstract clearly do not meet inclusion criteria | No |
| 63 | Patterns of ischemic injury on brain images in neonatal group B Streptococcal meningitis | 2018 | Title and abstract clearly do not meet inclusion criteria | No |
| 64 | Midbrain infarction presenting with monocular elevation palsy and ptosis: topographic lesion analysis | 2015 | Title and abstract clearly do not meet inclusion criteria | No |
| 65 | Traumatic bilateral common carotid artery dissection due to strangulation. A case report | 2006 | Title and abstract clearly do not meet inclusion criteria | No |
| 66 | Three-dimensional skeletal muscle architecture in the lower legs of living human infants | 2023 | Title and abstract clearly do not meet inclusion criteria | No |
| 67 | Consequences of intraventricular hemorrhage in a rabbit pup model | 2009 | Title and abstract clearly do not meet inclusion criteria | No |
| 68 | Hypoglycemic encephalopathy mimicking acute ischemic stroke in clinical presentation and magnetic resonance imaging: a case report | 2019 | Title and abstract clearly do not meet inclusion criteria | No |
| 69 | Gray and White Matter Correlates of Dysphagia in Progressive Supranuclear Palsy | 2021 | Title and abstract clearly do not meet inclusion criteria | No |
| 70 | Full Activation Profiles and Integrity of Corticospinal Pathways in Adults With Bilateral Spastic Cerebral Palsy | 2019 | No correlation analysis | No |
| 71 | Developmental neuroplasticity of the white matter connectome in children with perinatal stroke | 2020 | No correlation analysis | No |
| 72 | Imaging Developmental and Interventional Plasticity Following Perinatal Stroke | 2021 | review | No |
| 73 | The relation between neuroimaging and visual impairment in children and adolescents with cerebral palsy: A systematic review | 2024 | review | No |
| 74 | Thalamopeduncular Tumors in Pediatric Age: Advanced Preoperative Imaging to Define Safe Surgical Planning: A Multicentric Experience | 2023 | Title and abstract clearly do not meet inclusion criteria | No |
| 75 | 99mTc annexin V imaging of neonatal hypoxic brain injury | 2000 | Title and abstract clearly do not meet inclusion criteria | No |
| 76 | Muscle architecture in children with cerebral palsy and ankle contractures: an investigation using diffusion tensor imaging | 2019 | No correlation analysis | No |
| 77 | Functional and Structural Brain Connectivity in Children With Bilateral Cerebral Palsy Compared to Age-Related Controls and in Response to Intensive Rapid-Reciprocal Leg Training | 2022 | No correlation analysis | No |
| 78 | Tractography of white-matter tracts in very preterm infants: a 2-year follow-up study | 2013 | Title and abstract clearly do not meet inclusion criteria | No |
| 79 | Arterial spin-labelling perfusion MRI and outcome in neonates with hypoxic-ischemic encephalopathy | 2015 | Non-CP | No |
| 80 | Myth: cerebral palsy cannot be predicted by neonatal brain imaging | 2011 | review | No |
| 81 | Diffusion tensor imaging in a patient with cerebral palsy and hypersomnia | 2014 | Title and abstract clearly do not meet inclusion criteria | No |
| 82 | Rare Presentation of Midbrain Infarction: Isolated Medial Rectus Palsy | 2015 | No correlation analysis | No |
| 83 | Very preterm children at risk for developmental coordination disorder have brain alterations in motor areas | 2019 | No correlation analysis | No |
| 84 | Early diffusion-weighted magnetic resonance imaging findings in neonatal herpes encephalitis | 2006 | Non-CP | No |
| 85 | Long term motor function after neonatal stroke: Lesion localization above all | 2015 | Non-CP | No |
| 86 | Early Detection of Hypothermic Neuroprotection Using T2-Weighted Magnetic Resonance Imaging in a Mouse Model of Hypoxic Ischemic Encephalopathy | 2018 | Title and abstract clearly do not meet inclusion criteria | No |
| 87 | Concurrent decrease of brain white matter tracts' thicknesses and fractional anisotropy after antenatal hypoxia-ischemia detected with tract-based spatial statistics analysis | 2017 | No correlation analysis | No |
| 88 | Near-term fetal hypoxia-ischemia in rabbits: MRI can predict muscle tone abnormalities and deep brain injury | 2012 | Title and abstract clearly do not meet inclusion criteria | No |
| 89 | Fetal brain magnetic resonance imaging response acutely to hypoxia-ischemia predicts postnatal outcome | 2007 | Title and abstract clearly do not meet inclusion criteria | No |
| 90 | White matter injury correlates with hypertonia in an animal model of cerebral palsy | 2007 | Title and abstract clearly do not meet inclusion criteria | No |
| 91 | Unmyelinated axon loss with postnatal hypertonia after fetal hypoxia | 2014 | Title and abstract clearly do not meet inclusion criteria | No |
| 92 | Motor deficits are triggered by reperfusion-reoxygenation injury as diagnosed by MRI and by a mechanism involving oxidants | 2012 | Title and abstract clearly do not meet inclusion criteria | No |
| 93 | Spinal cord injury in hypertonic newborns after antenatal hypoxia-ischemia in a rabbit model of cerebral palsy | 2017 | Title and abstract clearly do not meet inclusion criteria | No |
| 94 | Developmental changes in diffusion anisotropy coincide with immature oligodendrocyte progression and maturation of compound action potential | 2005 | Title and abstract clearly do not meet inclusion criteria | No |
| 95 | Diffuse reduction of white matter connectivity in cerebral palsy with specific vulnerability of long range fiber tracts | 2013 | No correlation analysis | No |
| 96 | Brain structural connectivity increases concurrent with functional improvement: evidence from diffusion tensor MRI in children with cerebral palsy during therapy | 2015 | No correlation analysis | No |
| 97 | Diffusion tensor imaging shows different topographic involvement of the thalamus in progressive supranuclear palsy and corticobasal degeneration | 2009 | No correlation analysis | No |
| 98 | Caveats in diffusion tensor imaging interpretation | 2010 | Title and abstract clearly do not meet inclusion criteria | No |
| 99 | Quantitative analysis of brain pathology based on MRI and brain atlases--applications for cerebral palsy | 2011 | Title and abstract clearly do not meet inclusion criteria | No |
| 100 | Atlas-based analysis of neurodevelopment from infancy to adulthood using diffusion tensor imaging and applications for automated abnormality detection | 2010 | Title and abstract clearly do not meet inclusion criteria | No |
| 101 | Left hemispheric status epilepticus with crossed cerebellar diaschisis | 2018 | Title and abstract clearly do not meet inclusion criteria | No |
| 102 | Uncrossed corticospinal tract in health and genetic disorders: Review, case report, and clinical implications | 2021 | review | No |
| 103 | Cognition in cerebral palsy: White matter matters | 2021 | Title and abstract clearly do not meet inclusion criteria | No |
| 104 | Validity of semi-quantitative scale for brain MRI in unilateral cerebral palsy due to periventricular white matter lesions: Relationship with hand sensorimotor function and structural connectivity | 2015 | No correlation analysis | No |
| 105 | Corticopontocerebellar Connectivity Disruption in Congenital Hemiplegia | 2015 |  | Yes |
| 106 | The relationship between neuroimaging and motor outcome in children with cerebral palsy: A systematic review - Part A. Structural imaging | 2020 | review | No |
| 107 | Ischemic and hemorrhagic brain stem lesions mimicking diabetic ophthalmoplegia | 1995 | Title and abstract clearly do not meet inclusion criteria | No |
| 108 | An Individualized Approach to Neuroplasticity After Early Unilateral Brain Damage | 2019 | review | No |
| 109 | MRI and Motor Outcomes in Children with Cerebral Palsy | 2015 | Title and abstract clearly do not meet inclusion criteria | No |
| 110 | White matter changes associated with cognitive visual dysfunctions in children with cerebral palsy: A diffusion tensor imaging study | 2018 |  | Yes |
| 111 | Bilateral Thalamic Ischemic Stroke Secondary to Occlusion of the Artery of Percheron | 2018 | Title and abstract clearly do not meet inclusion criteria | No |
| 112 | ST3GAL5-Related Disorders: A Deficiency in Ganglioside Metabolism and a Genetic Cause of Intellectual Disability and Choreoathetosis | 2018 | Title and abstract clearly do not meet inclusion criteria | No |
| 113 | Regional microstructural damage and patterns of eye movement impairment: a DTI and video-oculography study in neurodegenerative parkinsonian syndromes | 2017 | Title and abstract clearly do not meet inclusion criteria | No |
| 114 | Motor Abilities in Adolescents Born Preterm Are Associated With Microstructure of the Corpus Callosum | 2019 | Non-CP | No |
| 115 | Effect of sensory and motor connectivity on hand function in pediatric hemiplegia | 2017 | Title and abstract clearly do not meet inclusion criteria | No |
| 116 | Neural Correlates of Impaired Grasp Function in Children with Unilateral Spastic Cerebral Palsy | 2023 | Title and abstract clearly do not meet inclusion criteria | No |
| 117 | Case Report: Brain Metastasis Confined to the Infarcted Area Following Stroke | 2020 | Title and abstract clearly do not meet inclusion criteria | No |
| 118 | A fatal case of infective endocarditis caused by an unusual suspect: Serratia marcescens | 2012 | Title and abstract clearly do not meet inclusion criteria | No |
| 119 | Clinical diagnosis of MM2-type sporadic Creutzfeldt-Jakob disease | 2005 | Title and abstract clearly do not meet inclusion criteria | No |
| 120 | Language comprehension in young people with severe cerebral palsy in relation to language tracts: a diffusion tensor imaging study | 2013 | Title and abstract clearly do not meet inclusion criteria | No |
| 121 | Diffusion-weighted imaging changes in cerebral watershed distribution following neonatal encephalopathy are not invariably associated with an adverse outcome | 2013 | Non-CP | No |
| 122 | Creutzfeldt-Jakob disease with paralysis of the unilateral vocal cord and soft palate | 2011 | Title and abstract clearly do not meet inclusion criteria | No |
| 123 | Cerebellar peduncle injury predicts motor impairments in preterm infants: A quantitative tractography study at term-equivalent age | 2018 |  | Yes |
| 124 | Pontine and cerebellar injury in neonatal hypoxic-ischemic encephalopathy: MRI features and clinical outcomes | 2020 | Non-CP | No |
| 125 | Long-term preservation of pharyngeal swallowing function in MM2-cortical-type sporadic Creutzfeldt-Jakob disease | 2021 | Title and abstract clearly do not meet inclusion criteria | No |
| 126 | Changes in the thalamus in atypical parkinsonism detected using shape analysis and diffusion tensor imaging | 2014 | Title and abstract clearly do not meet inclusion criteria | No |
| 127 | Segmental Diffusion Properties of the Corticospinal Tract and Motor Outcome in Hemiparetic Children With Perinatal Stroke | 2017 |  | Yes |
| 128 | White matter alterations and their associations with motor function in young adults born preterm with very low birth weight | 2018 | Title and abstract clearly do not meet inclusion criteria | No |
| 129 | Diffusion MRI in corticofugal fibers correlates with hand function in unilateral cerebral palsy | 2011 |  | Yes |
| 130 | Neuroimaging in cerebral palsy: Patterns of brain dysgenesis and injury | 2005 | review | No |
| 131 | Neuroimaging in spasticity and movement disorders | 2003 | review | No |
| 132 | Diffusion tensor imaging of periventricular leukomalacia shows affected sensory cortex white matter pathways | 2002 | No correlation analysis | No |
| 133 | Neuroimaging: applications in disorders of early brain development | 2000 | review | No |
| 134 | Sensory and motor deficits in children with cerebral palsy born preterm correlate with diffusion tensor imaging abnormalities in thalamocortical pathways | 2009 |  | Yes |
| 135 | Pathogenesis, neuroimaging and management in children with cerebral palsy born preterm | 2010 | Title and abstract clearly do not meet inclusion criteria | No |
| 136 | Ischemic Stroke due to Virologically-Confirmed Varicella Zoster Virus Vasculopathy: A Case Series | 2019 | Title and abstract clearly do not meet inclusion criteria | No |
| 137 | Characterization of Atypical Corticospinal Tract Microstructure and Hand Impairments in Early-Onset Hemiplegic Cerebral Palsy: Preliminary Findings | 2023 | No correlation analysis | No |
| 138 | White and gray matter development in human fetal, newborn and pediatric brains | 2006 | Title and abstract clearly do not meet inclusion criteria | No |
| 139 | Relationship Between Integrity of the Corpus Callosum and Bimanual Coordination in Children With Unilateral Spastic Cerebral Palsy | 2019 |  | Yes |
| 140 | Transient dysautonomia in an acute phase of encephalopathy with biphasic seizures and late reduced diffusion | 2017 | Title and abstract clearly do not meet inclusion criteria | No |
| 141 | [A case of non-traumatic direct carotid-cavernous fistula presenting with cerebral infarction] | 2012 | Title and abstract clearly do not meet inclusion criteria | No |
| 142 | One-and-a-half syndrome--two cases | 2007 | Title and abstract clearly do not meet inclusion criteria | No |
| 143 | Diffusion tensor brain imaging and tractography | 2002 | Title and abstract clearly do not meet inclusion criteria | No |
| 144 | Diffusion tensor analysis of corpus callosum in progressive supranuclear palsy | 2008 | Title and abstract clearly do not meet inclusion criteria | No |
| 145 | Limb-specific thalamocortical tracts are impaired differently in hemiplegic and diplegic subtypes of cerebral palsy | 2023 |  | Yes |
| 146 | Altered corpus callosum structure in adolescents with cerebral palsy: connection to gait and balance | 2023 |  | Yes |
| 147 | Retinal ganglion cell topography predicts visual field function in spastic cerebral palsy | 2020 | No correlation analysis | No |
| 148 | Diffusion Tensor Imaging Tractography Detecting Isolated Oculomotor Nerve Damage After Traumatic Brain Injury | 2017 | Title and abstract clearly do not meet inclusion criteria | No |
| 149 | Focal Cortical Dysplasia with hippocampal sclerosis | 2023 | Title and abstract clearly do not meet inclusion criteria | No |
| 150 | Aberrant Pyramidal Tract in Comparison with Pyramidal Tract on Diffusion Tensor Tractography: A Mini-Review | 2017 | review | No |
| 151 | Fetal and Neonatal Effects of N-Acetylcysteine When Used for Neuroprotection in Maternal Chorioamnionitis | 2016 | Non-CP | No |
| 152 | Dissecting aneurysm of vertebral artery manifestating as contralateral abducens nerve palsy | 2013 | No correlation analysis | No |
| 153 | Early Diagnosis of Spastic Cerebral Palsy in Infants with Periventricular White Matter Injury Using Diffusion Tensor Imaging | 2019 | No correlation analysis | No |
| 154 | Specific White Matter Lesions Related to Motor Dysfunction in Spastic Cerebral Palsy: A Meta-analysis of Diffusion Tensor Imaging Studies | 2020 | review | No |
| 155 | Structural network performance for early diagnosis of spastic cerebral palsy in periventricular white matter injury | 2021 |  | Yes |
| 156 | New means to assess neonatal inflammatory brain injury | 2015 | Non-CP | No |
| 157 | Diffusion tensor imaging of white matter and developmental outcome | 2008 | Title and abstract clearly do not meet inclusion criteria | No |
| 158 | Medial lemniscus lesion in pediatric hemiplegic patients without corticospinal tract and posterior thalamic radiation lesion | 2012 | Title and abstract clearly do not meet inclusion criteria | No |
| 159 | Isolated shoulder palsy diagnosed from needle EMG and an associated movement | 2015 | Title and abstract clearly do not meet inclusion criteria | No |
| 160 | [Cerebral peduncle infarction with pure dysarthria--case report] | 2007 | Title and abstract clearly do not meet inclusion criteria | No |
| 161 | Tuberculous meningitis presenting as mesencephalic infarction and syringomyelia | 2007 | Title and abstract clearly do not meet inclusion criteria | No |
| 162 | Early diffusion-weighted images in infants with subcortical leukomalacia | 2010 | Title and abstract clearly do not meet inclusion criteria | No |
| 163 | Isolated index finger palsy due to cortical infarction | 2014 | Title and abstract clearly do not meet inclusion criteria | No |
| 164 | Brain structural and microstructural alterations associated with cerebral palsy and motor impairments in adolescents born extremely preterm and/or extremely low birthweight | 2015 | No correlation analysis | No |
| 165 | Imaging of pediatric and congenital brain disease | 1992 | Title and abstract clearly do not meet inclusion criteria | No |
| 166 | Neurodevelopmental impairment is associated with altered white matter development in a cohort of school-aged children born very preterm | 2021 | Title and abstract clearly do not meet inclusion criteria | No |
| 167 | Different patterns of punctate white matter lesions in serially scanned preterm infants | 2014 | No correlation analysis | No |
| 168 | Artery of Percheron infarction a rare anatomical variant and a diagnostic challenge: Case report | 2021 | Title and abstract clearly do not meet inclusion criteria | No |
| 169 | Predictive Factors for the Occurrence of Visual and Ischemic Complications After Open Surgery for Paraclinoid Aneurysms of the Internal Carotid Artery | 2016 | Title and abstract clearly do not meet inclusion criteria | No |
| 170 | Pure motor trigeminal neuropathy in a woman with tegmental pontine infarction | 2013 | Title and abstract clearly do not meet inclusion criteria | No |
| 171 | Neonatal diffusion tensor brain imaging predicts later motor outcome in preterm neonates with white matter abnormalities | 2016 | Non-CP | No |
| 172 | Limb Length Discrepancy and Corticospinal Tract Disruption in Hemiplegic Cerebral Palsy | 2022 |  | Yes |
| 173 | Chronic fetal hypoxia affects axonal maturation in guinea pigs during development: A longitudinal diffusion tensor imaging and T2 mapping study | 2015 | Title and abstract clearly do not meet inclusion criteria | No |
| 174 | Motor function outcomes of pediatric patients with hemiplegic cerebral palsy after rehabilitation treatment: a diffusion tensor imaging study | 2015 | No correlation analysis | No |
| 175 | Modeling developmental plasticity after perinatal stroke: defining central therapeutic targets in cerebral palsy | 2013 | Title and abstract clearly do not meet inclusion criteria | No |
| 176 | Association between brain structural network efficiency at term-equivalent age and early development of cerebral palsy in very preterm infants | 2021 | Title and abstract clearly do not meet inclusion criteria | No |
| 177 | Neuro-imaging characteristics of sensory impairment in cerebral palsy; a systematic review | 2023 | review | No |
| 178 | Unusual presentation of acute encephalopathy with biphasic seizures and late reduced diffusion in Miller-Dieker syndrome | 2022 | Title and abstract clearly do not meet inclusion criteria | No |
| 179 | [Bilateral Medial Medulla Infarction Mimicking Guillain-Barré Syndrome and its Variants] | 2020 | Title and abstract clearly do not meet inclusion criteria | No |
| 180 | Anisotropy of transcallosal motor fibres indicates functional impairment in children with periventricular leukomalacia | 2011 | Title and abstract clearly do not meet inclusion criteria | No |
| 181 | Sensory tractography and robot-quantified proprioception in hemiparetic children with perinatal stroke | 2017 | Title and abstract clearly do not meet inclusion criteria | No |
| 182 | Corticospinal tract diffusion properties and robotic visually guided reaching in children with hemiparetic cerebral palsy | 2018 |  | Yes |
| 183 | Dyskinesia Impairment Scale scores in Dutch pre-school children after neonatal therapeutic hypothermia | 2020 | Non-CP | No |
| 184 | Serial evaluation of swallowing function in a long-term survivor of V180I genetic Creutzfeldt-Jakob disease | 2020 | Title and abstract clearly do not meet inclusion criteria | No |
| 185 | Using diffusion tensor imaging to identify corticospinal tract projection patterns in children with unilateral spastic cerebral palsy | 2017 |  | Yes |
| 186 | Early-onset or rapidly progressive scoliosis in children: check the eyes! | 2013 | Title and abstract clearly do not meet inclusion criteria | No |
| 187 | Changes in diffusion tensor tractographic findings associated with constraint-induced movement therapy in young children with cerebral palsy | 2014 | Title and abstract clearly do not meet inclusion criteria | No |
| 188 | Isolated Bilateral Cerebral Peduncular Infarction Manifesting Pseudobulbar Palsy and Quadriparesis: a Case Report | 2021 | Title and abstract clearly do not meet inclusion criteria | No |
| 189 | Corticoreticular tract lesion in children with developmental delay presenting with gait dysfunction and trunk instability | 2017 | Title and abstract clearly do not meet inclusion criteria | No |
| 190 | Early diffusion-weighted MRI and 1H-Magnetic Resonance Spectroscopy in asphyxiated full-term neonates | 2005 | Title and abstract clearly do not meet inclusion criteria | No |
| 191 | Diffusion tensor imaging is associated with motor outcomes of very preterm born children at 11 years of age | 2020 | Title and abstract clearly do not meet inclusion criteria | No |
| 192 | Starting a DBS service for children: It's not the latitude but the attitude - Establishment of the paediatric DBS centre in Northern Finland | 2022 | Title and abstract clearly do not meet inclusion criteria | No |
| 193 | Artery of Percheron infarction: review of literature with a case report | 2015 | review | No |
| 194 | White matter integrity in dyskinetic cerebral palsy: Relationship with intelligence quotient and executive function | 2017 |  | Yes |
| 195 | White matter microstructure and receptive vocabulary in children with cerebral palsy: The role of interhemispheric connectivity | 2023 |  | Yes |
| 196 | Frontal interhemispheric structural connectivity, attention, and executive function in children with perinatal stroke | 2022 | Title and abstract clearly do not meet inclusion criteria | No |
| 197 | Changes in a cerebellar peduncle lesion in a patient with Dandy-Walker malformation: A diffusion tensor imaging study | 2013 | Title and abstract clearly do not meet inclusion criteria | No |
| 198 | Analysis of structure-function network decoupling in the brain systems of spastic diplegic cerebral palsy | 2017 | Title and abstract clearly do not meet inclusion criteria | No |
| 199 | Isolated inferior rectus palsy due to midbrain infarction detected by diffusion-weighted MRI | 2006 | Title and abstract clearly do not meet inclusion criteria | No |
| 200 | Motor pathway injury in patients with periventricular leucomalacia and spastic diplegia | 2011 |  | Yes |
| 201 | Surgical and molecular considerations in the treatment of pediatric thalamopeduncular tumors | 2017 | Title and abstract clearly do not meet inclusion criteria | No |
| 202 | Diffusion-tensor MR imaging and fiber tractography: a new method of describing aberrant fiber connections in developmental CNS anomalies | 2005 | Title and abstract clearly do not meet inclusion criteria | No |
| 203 | Safety and feasibility of countering neurological impairment by intravenous administration of autologous cord blood in cerebral palsy | 2012 | Title and abstract clearly do not meet inclusion criteria | No |
| 204 | Diffusion tensor magnetic resonance imaging of microstructural abnormalities in children with brain injury | 2003 | Title and abstract clearly do not meet inclusion criteria | No |
| 205 | Advanced fiber tracking in early acquired brain injury causing cerebral palsy | 2015 | Title and abstract clearly do not meet inclusion criteria | No |
| 206 | A Semi-Supervised Graph Convolutional Network for Early Prediction of Motor Abnormalities in Very Preterm Infants | 2023 | Title and abstract clearly do not meet inclusion criteria | No |
| 207 | [Research on brain white matter network in cerebral palsy infant] | 2017 | Title and abstract clearly do not meet inclusion criteria | No |
| 208 | Contemporary therapeutic strategies for occlusion of the artery of Percheron: a review of the literature | 2015 | review | No |
| 209 | [Analysis of 58 neonatal cases with cerebral infarction] | 2013 | Non-CP | No |
| 210 | Structure of brain grey and white matter in infants with spastic cerebral palsy and periventricular white matter injury | 2023 | Title and abstract clearly do not meet inclusion criteria | No |
| 211 | Transclival Approach for Resection of a Pontine Cavernous Malformation: 2-Dimensional Operative Video | 2020 | Title and abstract clearly do not meet inclusion criteria | No |
| 212 | Peak Width of Skeletonized Mean Diffusivity as a Marker of Diffuse Cerebrovascular Damage | 2020 | Title and abstract clearly do not meet inclusion criteria | No |
| 213 | Perfusion and diffusion tensor imaging in a patient with locked-in syndrome after neurosurgical vascular bypass and endovascular embolization of a basilar artery aneurysm: case report | 2006 | Title and abstract clearly do not meet inclusion criteria | No |
| 214 | Association of Acute Infarct Topography With Development of Cerebral Palsy and Neurologic Impairment in Neonates With Stroke | 2023 | Title and abstract clearly do not meet inclusion criteria | No |
| 215 | Pediatric ASPECTS predicts outcomes following acute symptomatic neonatal arterial stroke | 2020 | Title and abstract clearly do not meet inclusion criteria | No |
| 216 | Upper limb function and cortical organization in youth with unilateral cerebral palsy | 2014 | Title and abstract clearly do not meet inclusion criteria | No |
| 217 | Significance of MRI in diagnosis and differential diagnosis of Parkinson's disease | 2010 | Title and abstract clearly do not meet inclusion criteria | No |
| 218 | [Differential diagnosis of parkinsonian syndromes using MRI] | 2010 | Title and abstract clearly do not meet inclusion criteria | No |
| 219 | The relationship between neuroimaging and motor outcome in children with cerebral palsy: A systematic review-Part B diffusion imaging and tractography | 2020 | review | No |
| 220 | White matter characteristics of motor, sensory and interhemispheric tracts underlying impaired upper limb function in children with unilateral cerebral palsy | 2020 | Title and abstract clearly do not meet inclusion criteria | No |
| 221 | An unusual cause of pseudomedian nerve palsy | 2011 | Title and abstract clearly do not meet inclusion criteria | No |
| 222 | Resting State and Diffusion Neuroimaging Predictors of Clinical Improvements Following Constraint-Induced Movement Therapy in Children With Hemiplegic Cerebral Palsy | 2015 | Title and abstract clearly do not meet inclusion criteria | No |
| 223 | Spatial analysis of diffusion tensor tractography statistics along the inferior fronto-occipital fasciculus with application in progressive supranuclear palsy | 2013 | No correlation analysis | No |
| 224 | The Challenge of Diffusion Magnetic Resonance Imaging in Cerebral Palsy: A Proposed Method to Identify White Matter Pathways | 2023 | Title and abstract clearly do not meet inclusion criteria | No |
| 225 | The Effects of Low Frequency Repetitive Transcranial Magnetic Stimulation on White Matter Structural Connectivity in Children with Cerebral Palsy | 2018 | Title and abstract clearly do not meet inclusion criteria | No |
| 226 | Neonatal neurobehavior after therapeutic hypothermia for hypoxic ischemic encephalopathy | 2015 | Title and abstract clearly do not meet inclusion criteria | No |
| 227 | Quantitative analysis of magnetic resonance images and neurological outcome in encephalopathic neonates treated with whole-body hypothermia | 2010 | Title and abstract clearly do not meet inclusion criteria | No |
| 228 | Magnetic resonance imaging--insights into brain injury and outcomes in premature infants | 2009 | Title and abstract clearly do not meet inclusion criteria | No |
| 229 | A case of posterior cerebral artery occlusion that developed into an artery of Percheron infarction | 2022 | Title and abstract clearly do not meet inclusion criteria | No |
| 230 | Long-Term Neuropathological Changes Associated with Cerebral Palsy in a Nonhuman Primate Model of Hypoxic-Ischemic Encephalopathy | 2017 | Non-CP | No |
| 231 | Discovering the sense of touch: protocol for a randomised controlled trial examining the efficacy of a somatosensory discrimination intervention for children with hemiplegic cerebral palsy | 2018 | Title and abstract clearly do not meet inclusion criteria | No |
| 232 | [Assessment of motor and sensory pathways of the brain using diffusion-tensor tractography in children with cerebral palsy] | 2014 | Title and abstract clearly do not meet inclusion criteria | No |
| 233 | Neonatal Functional and Structural Connectivity Are Associated with Cerebral Palsy at Two Years of Age | 2020 | Title and abstract clearly do not meet inclusion criteria | No |
| 234 | Macrostructural and Microstructural Brain Lesions Relate to Gait Pathology in Children With Cerebral Palsy | 2016 |  | Yes |
| 235 | Emotional facial palsy following striato-capsular infarction | 2008 | Title and abstract clearly do not meet inclusion criteria | No |
| 236 | Umbilical cord blood therapy potentiated with erythropoietin for children with cerebral palsy: a double-blind, randomized, placebo-controlled tria | 2013 | No correlation analysis | No |
| 237 | Potentiation of cord blood cell therapy with erythropoietin for children with CP: a 2 × 2 factorial randomized placebo-controlled trial | 2020 | Title and abstract clearly do not meet inclusion criteria | No |
| 238 | Reliability of fractional anisotropy measurement for children with cerebral palsy | 2014 | No correlation analysis | No |
| 239 | SARS-CoV-19-associated Rhino-orbital and cerebral mucormycosis: clinical and radiological presentations | 2022 | Title and abstract clearly do not meet inclusion criteria | No |
| 240 | Magnetic resonance imaging pattern recognition in childhood bilateral basal ganglia disorders | 2020 | review | No |
| 241 | [Acute pseudobulbar paralysis: the use of diffusion techniques with magnetic resonance] | 1999 | Title and abstract clearly do not meet inclusion criteria | No |
| 242 | At the boundary of the self: the insular cortex in patients with childhood-onset schizophrenia, their healthy siblings, and normal volunteers | 2014 | Title and abstract clearly do not meet inclusion criteria | No |
| 243 | The Role of the Corpus Callosum in Pediatric Dysphagia: Preliminary Findings from a Diffusion Tensor Imaging Study in Children with Unilateral Spastic Cerebral Palsy | 2017 |  | Yes |
| 244 | [Multiple cerebral hemorrhages due to varicella-zoster virus vasculopathy presenting as cranial nerve palsy] | 2020 | Title and abstract clearly do not meet inclusion criteria | No |
| 245 | Quantitative cranial magnetic resonance imaging in neonatal hypoxic-ischemic encephalopathy | 2012 | Non-CP | No |
| 246 | Radial artery grafts for symptomatic cavernous carotid aneurysms in elderly patients | 2011 | Title and abstract clearly do not meet inclusion criteria | No |
| 247 | Fiber-tracking techniques can predict the degree of neurologic impairment for periventricular leukomalacia | 2008 | Title and abstract clearly do not meet inclusion criteria | No |
| 248 | Diffusion tensor imaging in children with periventricular leukomalacia: variability of injuries to white matter tracts | 2007 | No correlation analysis | No |
| 249 | [Two cases of top of the basilar syndrome with onset seizure] | 2005 | Title and abstract clearly do not meet inclusion criteria | No |
| 250 | Diffusion tensor imaging on teenagers, born at term with moderate hypoxic-ischemic encephalopathy | 2005 | Non-CP | No |
| 251 | Relationship between cortex and pulvinar abnormalities on diffusion-weighted imaging in status epilepticus | 2016 | Title and abstract clearly do not meet inclusion criteria | No |
| 252 | [A case of bilateral thalamic infarction with the characteristic MRI finding] | 2022 | Title and abstract clearly do not meet inclusion criteria | No |
| 253 | Reversible ischemic neurological deficit (RIND) due to exercise testing for the diagnosis of angina pectoris | 2003 | Title and abstract clearly do not meet inclusion criteria | No |
| 254 | Neurite orientation dispersion and density imaging quantifies corticospinal tract microstructural organization in children with unilateral cerebral palsy | 2019 | Title and abstract clearly do not meet inclusion criteria | No |
| 255 | Magnetic Resonance Imaging in Tauopathy Animal Models | 2021 | Title and abstract clearly do not meet inclusion criteria | No |
| 256 | Artery of Percheron Infarct: ARarity Not to be Missed | 2017 | Title and abstract clearly do not meet inclusion criteria | No |
| 257 | Empyema necessitatis due to Pseudomonas aeruginosa in a child with cerebral palsy | 2020 | Title and abstract clearly do not meet inclusion criteria | No |
| 258 | Internal carotid artery dissection heralded by an oculomotor nerve palsy: case report and literature review | 2011 | review | No |
| 259 | Isolated nuclear facial palsy, a rare variant of pure motor lacunar stroke | 2008 | Title and abstract clearly do not meet inclusion criteria | No |
| 260 | The utility of the fronto-temporal horn ratio on cranial ultrasound in premature newborns: a ventriculomegaly marker | 2021 | Title and abstract clearly do not meet inclusion criteria | No |
| 261 | [Unilateral external ophthalmoplegia caused by ipsilateral oculomotor nuclear lesion: analysis with diffusion weighted MRI] | 1994 | Title and abstract clearly do not meet inclusion criteria | No |
| 262 | Clinical Study of Eleven Patients with Midbrain Infarction-Induced Oculomotor Nerve Palsy | 2016 | Title and abstract clearly do not meet inclusion criteria | No |
| 263 | Conjugate Eye Deviation Caused by Upper Medial Medullary Infarction: A Case Report | 2018 | Title and abstract clearly do not meet inclusion criteria | No |
| 264 | Apparent diffusion coefficient measurements in progressive supranuclear palsy | 2000 | Title and abstract clearly do not meet inclusion criteria | No |
| 265 | Unilateral opercular infarction presenting with Foix-Chavany-Marie Syndrome | 2014 | Title and abstract clearly do not meet inclusion criteria | No |
| 266 | Quantitative evaluation of brain development using anatomical MRI and diffusion tensor imaging | 2013 | No correlation analysis | No |
| 267 | Reprint of "Quantitative evaluation of brain development using anatomical MRI and diffusion tensor imaging" | 2014 | Title and abstract clearly do not meet inclusion criteria | No |
| 268 | [Orolingual angioedema as complication after rt-PA in stroke patient treated with ACE inhibitor] | 2008 | Title and abstract clearly do not meet inclusion criteria | No |
| 269 | Low signal intensity and increased anisotropy on magnetic resonance imaging in the white matter lesion after head trauma: unrecognized findings of diffuse axonal injury | 2007 | Title and abstract clearly do not meet inclusion criteria | No |
| 270 | Diffusion tensor imaging in arginase deficiency reveals damage to corticospinal tracts | 2010 | No correlation analysis | No |
| 271 | [Creutzfeldt-Jakob Disease: Atypical Presentation of a Very Rare Disease] | 2021 | Title and abstract clearly do not meet inclusion criteria | No |
| 272 | Visual perception in preterm children: what are we currently measuring? | 2011 | Title and abstract clearly do not meet inclusion criteria | No |
| 273 | Diffusion tensor imaging and voxel based morphometry study in early progressive supranuclear palsy | 2006 | Title and abstract clearly do not meet inclusion criteria | No |
| 274 | Neuroimaging biomarkers of preterm brain injury: toward developing the preterm connectome | 2012 | Title and abstract clearly do not meet inclusion criteria | No |
| 275 | Assessment of the structural brain network reveals altered connectivity in children with unilateral cerebral palsy due to periventricular white matter lesions | 2014 |  | Yes |
| 276 | Fixel-based analysis reveals alterations is brain microstructure and macrostructure of preterm-born infants at term equivalent age | 2018 | Title and abstract clearly do not meet inclusion criteria | No |
| 277 | Brain microstructure and morphology of very preterm-born infants at term equivalent age: Associations with motor and cognitive outcomes at 1 and 2 years | 2020 | Title and abstract clearly do not meet inclusion criteria | No |
| 278 | Altered White Matter Connectivity Associated with Intergyral Brain Disorganization in Hemiplegic Cerebral Palsy | 2019 | Title and abstract clearly do not meet inclusion criteria | No |
| 279 | Cortical somatosensory reorganization in children with spastic cerebral palsy: a multimodal neuroimaging study | 2014 | Title and abstract clearly do not meet inclusion criteria | No |
| 280 | Reorganization of the somatosensory cortex in hemiplegic cerebral palsy associated with impaired sensory tracts | 2018 | Title and abstract clearly do not meet inclusion criteria | No |
| 281 | Maturation of Corticospinal Tracts in Children With Hemiplegic Cerebral Palsy Assessed by Diffusion Tensor Imaging and Transcranial Magnetic Stimulation | 2019 | Title and abstract clearly do not meet inclusion criteria | No |
| 282 | Advanced neuroimaging and its role in predicting neurodevelopmental outcomes in very preterm infants | 2016 | Title and abstract clearly do not meet inclusion criteria | No |
| 283 | Early Detection of Cerebral Palsy Using Sensorimotor Tract Biomarkers in Very Preterm Infants | 2019 | Title and abstract clearly do not meet inclusion criteria | No |
| 284 | Neuroradiological and neurophysiological characteristics of patients with dyskinetic cerebral palsy | 2014 | Title and abstract clearly do not meet inclusion criteria | No |
| 285 | Increased GABA-A receptor binding and reduced connectivity at the motor cortex in children with hemiplegic cerebral palsy: a multimodal investigation using 18F-fluoroflumazenil PET, immunohistochemistry, and MR imaging | 2013 | Title and abstract clearly do not meet inclusion criteria | No |
| 286 | Peripheral type facial palsy in a patient with dorsolateral medullary infarction with infranuclear involvement of the caudal pons | 2008 | Title and abstract clearly do not meet inclusion criteria | No |
| 287 | Different clinical courses of various radiologic findings in fibromuscular dysplasia during a 7-year follow-up: A case report | 2020 | Title and abstract clearly do not meet inclusion criteria | No |
| 288 | Rapid Regression of Carotid Artery Stenosis Shortly after Intensive Medical Therapy | 2022 | Title and abstract clearly do not meet inclusion criteria | No |
| 289 | Correlation of basal ganglia magnetic resonance spectroscopy with Apgar score in perinatal asphyxia | 1999 | Title and abstract clearly do not meet inclusion criteria | No |
| 290 | Neural correlates of developmental coordination disorder | 2013 | Title and abstract clearly do not meet inclusion criteria | No |
| 291 | White Matter Injury and General Movements in High-Risk Preterm Infants | 2017 | Title and abstract clearly do not meet inclusion criteria | No |
| 292 | Multimodal assessment of motor pathways and intracortical connections in functional hemispherectomy | 2020 | Title and abstract clearly do not meet inclusion criteria | No |
| 293 | Role of diffusion tensor imaging as an independent predictor of cognitive and language development in extremely low-birth-weight infants | 2014 | Title and abstract clearly do not meet inclusion criteria | No |
| 294 | Effect of antenatal magnesium sulphate on MRI biomarkers of white matter development at term equivalent age: The magnum study | 2020 | Title and abstract clearly do not meet inclusion criteria | No |
| 295 | Effect of antenatal magnesium sulphate on MRI biomarkers of white matter development at term equivalent age: The MagNUM Study | 2022 | Title and abstract clearly do not meet inclusion criteria | No |
| 296 | Conjugate downward and upward vertical gaze palsy due to unilateral rostral midbrain infarction | 2012 | Title and abstract clearly do not meet inclusion criteria | No |
| 297 | Deep Brain Stimulation and Hypoxemic Perinatal Encephalopathy: State of Art and Perspectives | 2021 | Title and abstract clearly do not meet inclusion criteria | No |
| 298 | Exploring structural connectomes in children with unilateral cerebral palsy using graph theory | 2023 | Title and abstract clearly do not meet inclusion criteria | No |
| 299 | Neuroregenerative potential of intravenous G-CSF and autologous peripheral blood stem cells in children with cerebral palsy: a randomized, double-blind, cross-over study | 2017 | Title and abstract clearly do not meet inclusion criteria | No |
| 300 | DTI correlates of cognition in term children with spastic diplegic cerebral palsy | 2013 |  | Yes |
| 301 | Neonatal brain injury influences structural connectivity and childhood functional outcomes | 2022 | Non-CP | No |
| 302 | MR Imaging of hypoxic ischemic encephalopathy - Distribution Patterns and ADC value correlations | 2018 | Title and abstract clearly do not meet inclusion criteria | No |
| 303 | Pseudoperipheral palsy due to cortical infarction | 2009 | Title and abstract clearly do not meet inclusion criteria | No |
| 304 | Therapeutic effects of an anti-gravity locomotor training (AlterG) on postural balance and cerebellum structure in children with Cerebral Palsy | 2017 | Title and abstract clearly do not meet inclusion criteria | No |
| 305 | Changes in White Matter Integrity following Intensive Voice Treatment (LSVT LOUD®) in Children with Cerebral Palsy and Motor Speech Disorders | 2017 | Title and abstract clearly do not meet inclusion criteria | No |
| 306 | Surface-Based fMRI-Driven Diffusion Tractography in the Presence of Significant Brain Pathology: A Study Linking Structure and Function in Cerebral Palsy | 2016 |  | Yes |
| 307 | Correction: Surface-Based fMRI-Driven Diffusion Tractography in the Presence of Significant Brain Pathology: A Study Linking Structure and Function in Cerebral Palsy | 2016 | Title and abstract clearly do not meet inclusion criteria | No |
| 308 | Midbrain and bilateral paramedian | 2017 | Title and abstract clearly do not meet inclusion criteria | No |
| 309 | An Australian population study of factors associated with MRI patterns in cerebral palsy | 2014 | Title and abstract clearly do not meet inclusion criteria | No |
| 310 | Midbrain and bilateral paramedian thalamic stroke due to artery of Percheron occlusion | 2016 | Title and abstract clearly do not meet inclusion criteria | No |
| 311 | Comparing quantitative tractography metrics of motor and sensory pathways in children with periventricular leukomalacia and different levels of gross motor function | 2012 |  | Yes |
| 312 | Detection of focal cerebral injury using diffusion tensor magnetic resonance imaging in a boy with becker muscular dystrophy | 2009 | Title and abstract clearly do not meet inclusion criteria | No |
| 313 | Diffusion tensor imaging study of the response to constraint-induced movement therapy of children with hemiparetic cerebral palsy and adults with chronic stroke | 2014 | Title and abstract clearly do not meet inclusion criteria | No |
| 314 | Isolated acute pseudobulbar palsy with infarction of artery of percheron: case report and literature review | 2021 | review | No |
| 315 | Diffusion-weighted brain imaging study of patients with clinical diagnosis of corticobasal degeneration, progressive supranuclear palsy and Parkinson's disease | 2008 | Title and abstract clearly do not meet inclusion criteria | No |
| 316 | Communication skills in children aged 6-8 years, without cerebral palsy cooled for neonatal hypoxic-ischemic encephalopathy | 2022 | Non-CP | No |
| 317 | Corpus Callosum Integrity Relates to Improvement of Upper-Extremity Function Following Intensive Rehabilitation in Children With Unilateral Spastic Cerebral Palsy | 2021 | Title and abstract clearly do not meet inclusion criteria | No |
| 318 | Neonatal erythropoietin mitigates impaired gait, social interaction and diffusion tensor imaging abnormalities in a rat model of prenatal brain injury | 2018 | Non-CP | No |
| 319 | Neonatal brain structure on MRI and diffusion tensor imaging, sex, and neurodevelopment in very-low-birthweight preterm children | 2009 | Title and abstract clearly do not meet inclusion criteria | No |
| 320 | Movement disorders due to bilirubin toxicity | 2015 | Title and abstract clearly do not meet inclusion criteria | No |
| 321 | MRI structural connectivity, disruption of primary sensorimotor pathways, and hand function in cerebral palsy | 2011 | Title and abstract clearly do not meet inclusion criteria | No |
| 322 | Neonatal DTI early after birth predicts motor outcome in preterm infants with periventricular hemorrhagic infarction | 2015 | Title and abstract clearly do not meet inclusion criteria | No |
| 323 | The extent of intrauterine growth restriction determines the severity of cerebral injury and neurobehavioural deficits in rodents | 2017 | Title and abstract clearly do not meet inclusion criteria | No |
| 324 | Perimesencephalic nonaneurysmal subarachnoid hemorrhage with unilateral third cranial nerve palsy: two case reports | 2012 | Title and abstract clearly do not meet inclusion criteria | No |
| 325 | Imaging biomarkers in Parkinson's disease and Parkinsonian syndromes: current and emerging concepts | 2017 | Title and abstract clearly do not meet inclusion criteria | No |
| 326 | Neuroimaging Advances in Parkinson's Disease and Atypical Parkinsonian Syndromes | 2020 | Title and abstract clearly do not meet inclusion criteria | No |
| 327 | Predicting motor outcome in preterm infants from very early brain diffusion MRI using a deep learning convolutional neural network (CNN) model | 2020 | Title and abstract clearly do not meet inclusion criteria | No |
| 328 | Soleus muscle weakness in cerebral palsy: Muscle architecture revealed with Diffusion Tensor Imaging | 2019 | Duplicate Article | No |
| 329 | Correction: Soleus muscle weakness in cerebral palsy: Muscle architecture revealed with Diffusion Tensor Imaging | 2020 | No correlation analysis | No |
| 330 | Isolated cerebral mucormycosis: A case discussion | 2023 | Title and abstract clearly do not meet inclusion criteria | No |
| 331 | In vivo evaluation of white matter pathology in patients of progressive supranuclear palsy using TBSS | 2012 | Title and abstract clearly do not meet inclusion criteria | No |
| 332 | Medial posterior choroidal artery territory infarction associated with tumor removal in the pineal/tectum/thalamus region through the occipital transtentorial approach | 2013 | Title and abstract clearly do not meet inclusion criteria | No |
| 333 | Six Months Guided Exercise Therapy Improves Motor Abilities and White Matter Connectivity in Children with Cerebral Palsy | 2020 | Title and abstract clearly do not meet inclusion criteria | No |
| 334 | Characterisation of the Corticospinal Tract Using Diffusion Magnetic Resonance Imaging in Unilateral and Bilateral Cerebral Palsy Patients | 2018 | Title and abstract clearly do not meet inclusion criteria | No |
| 335 | Experimental cerebral palsy causes microstructural brain damage in areas associated to motor deficits but no spatial memory impairments in the developing rat | 2021 | Title and abstract clearly do not meet inclusion criteria | No |
| 336 | Grey and White Matter Clinico-Anatomical Correlates of Disinhibition in Neurodegenerative Disease | 2016 | Title and abstract clearly do not meet inclusion criteria | No |
| 337 | Corticospinal tract abnormalities and ventricular dilatation: A transdiagnostic comparative tractography study | 2021 | Title and abstract clearly do not meet inclusion criteria | No |
| 338 | Eight-and-a-half syndrome | 2009 | Title and abstract clearly do not meet inclusion criteria | No |
| 339 | [A Case of Moyamoya Disease with Postoperative Cerebral Hyperperfusion Syndrome Followed by Cerebral Infarction due to Watershed Shift] | 2018 | Title and abstract clearly do not meet inclusion criteria | No |
| 340 | Imaging functional motor connectivity in hemiparetic children with perinatal stroke | 2019 | Title and abstract clearly do not meet inclusion criteria | No |
| 341 | Normal diffusion-weighted imaging in cerebral air embolism complicating angiography | 2000 | Title and abstract clearly do not meet inclusion criteria | No |
| 342 | New insights into the pathology of white matter tracts in cerebral palsy from diffusion magnetic resonance imaging: a systematic review | 2012 | review | No |
| 343 | Extent of altered white matter in unilateral and bilateral periventricular white matter lesions in children with unilateral cerebral palsy | 2016 |  | Yes |
| 344 | Structural connectivity of the anterior cingulate in children with unilateral cerebral palsy due to white matter lesions | 2015 |  | Yes |
| 345 | Imaging Predictors of Improvement From a Motor Learning-Based Intervention for Children With Unilateral Cerebral Palsy | 2016 |  | Yes |
| 346 | Midbrain ischemia presenting as vertical gaze palsy: value of diffusion-weighted magnetic resonance imaging | 2004 | Title and abstract clearly do not meet inclusion criteria | No |
| 347 | Brain magnetic resonance imaging techniques in the diagnosis of parkinsonian syndromes | 2010 | Title and abstract clearly do not meet inclusion criteria | No |
| 348 | Superior alternating hemiplegia (Weber's syndrome)- Case report | 2022 | Title and abstract clearly do not meet inclusion criteria | No |
| 349 | Selective Hand Motor Cortex Lesions Masquerading as "Pseudoperipheral Nerve Palsy" | 2020 | Title and abstract clearly do not meet inclusion criteria | No |
| 350 | [A case of cerebral embolism with Coronavirus disease 2019] | 2021 | Title and abstract clearly do not meet inclusion criteria | No |
| 351 | [A case of cerebral embolism caused by Cardiobacterium hominis endocarditis] | 2013 | Title and abstract clearly do not meet inclusion criteria | No |
| 352 | MRI-based radiologic scoring system for extent of brain injury in children with hemiplegia | 2014 | review | No |
| 353 | Diffusion tensor imaging demonstrates focal lesions of the corticospinal tract in hemiparetic patients with cerebral palsy | 2007 | No correlation analysis | No |
| 354 | Improvement of Gait Dysfunction after Applying a Hinged Ankle-Foot Orthosis in a Hemiplegic Cerebral Palsy Patient with Disrupted Medial Lemniscus: A Case Report | 2021 | Title and abstract clearly do not meet inclusion criteria | No |
| 355 | Diffusion tensor tractography can predict hemiparesis in infants with high risk factors | 2009 | No correlation analysis | No |
| 356 | Motor function and white matter connectivity in children cooled for neonatal encephalopathy | 2021 | Title and abstract clearly do not meet inclusion criteria | No |
| 357 | Disrupted brain connectivity in children treated with therapeutic hypothermia for neonatal encephalopathy | 2021 | Title and abstract clearly do not meet inclusion criteria | No |
| 358 | Mammillary body abnormalities and cognitive outcomes in children cooled for neonatal encephalopathy | 2023 | Title and abstract clearly do not meet inclusion criteria | No |
| 359 | Diffusion Imaging of Cerebral Diaschisis in Neonatal Arterial Ischemic Stroke | 2019 | Title and abstract clearly do not meet inclusion criteria | No |
| 360 | Imaging cerebral palsy | 2013 | Title and abstract clearly do not meet inclusion criteria | No |
| 361 | Movement disorders | 2016 | Title and abstract clearly do not meet inclusion criteria | No |
| 362 | Diffusion Tensor Imaging to Predict Neurodevelopmental Impairment in Infants after Hypoxic-Ischemic Injury | 2023 | No correlation analysis | No |
| 363 | Widespread diffusion changes differentiate Parkinson's disease and progressive supranuclear palsy | 2018 | Title and abstract clearly do not meet inclusion criteria | No |
| 364 | Clinical features and imaging markers of small vessel disease in symptomatic acute subcortical cerebral microinfarcts | 2022 | Title and abstract clearly do not meet inclusion criteria | No |
| 365 | Quantitative diffusion tensor imaging in cerebral palsy due to periventricular white matter injury | 2005 | No correlation analysis | No |
| 366 | MRI combined with early clinical variables are excellent outcome predictors for newborn infants undergoing therapeutic hypothermia after perinatal asphyxia | 2021 | Title and abstract clearly do not meet inclusion criteria | No |
| 367 | Concurrent erythropoietin and hypothermia treatment improve outcomes in a term nonhuman primate model of perinatal asphyxia | 2013 | Title and abstract clearly do not meet inclusion criteria | No |
| 368 | Correlation of quantitative sensorimotor tractography with clinical grade of cerebral palsy | 2010 |  | Yes |
| 369 | Treatment-induced plasticity in cerebral palsy: a diffusion tensor imaging study | 2008 | Title and abstract clearly do not meet inclusion criteria | No |
| 370 | Changes in the integrity of thalamocortical connections are associated with sensorimotor deficits in children with congenital hemiplegia | 2015 |  | Yes |
| 371 | Reduced integrity of sensorimotor projections traversing the posterior limb of the internal capsule in children with congenital hemiparesis | 2014 | Title and abstract clearly do not meet inclusion criteria | No |
| 372 | Complete lingual palsy from bilateral Dejerine syndrome (bilateral medial medullary stroke) | 2016 | Title and abstract clearly do not meet inclusion criteria | No |
| 373 | Isolated shoulder palsy due to a cortical infarction | 2011 | Title and abstract clearly do not meet inclusion criteria | No |
| 374 | Skew Deviation: Case Report and Review of the Literature | 2017 | Title and abstract clearly do not meet inclusion criteria | No |
| 375 | MR imaging of term infants with hypoxic-ischaemic encephalopathy as a predictor of neurodevelopmental outcome and late MRI appearances | 2010 | Title and abstract clearly do not meet inclusion criteria | No |
| 376 | Ulnar nerve palsy-like motor and sensory loss caused by a small cortical infarct | 2012 | Title and abstract clearly do not meet inclusion criteria | No |
| 377 | [A case of successful thromobolytic therapy in a patient with cerebral embolism during angiography] | 1999 | Title and abstract clearly do not meet inclusion criteria | No |
| 378 | Aortic dissection diagnosed on stroke computed tomography protocol: a case report | 2021 | Title and abstract clearly do not meet inclusion criteria | No |
| 379 | [State of bilateral opercular disorder and pseudobulbar paralysis of late onset in unilateral perisylvian dysplasia] | 1997 | Title and abstract clearly do not meet inclusion criteria | No |
| 380 | Human parechovirus causes encephalitis with white matter injury in neonates | 2008 | Title and abstract clearly do not meet inclusion criteria | No |
| 381 | Mutation in the AP4M1 gene provides a model for neuroaxonal injury in cerebral palsy | 2009 | Title and abstract clearly do not meet inclusion criteria | No |
| 382 | Selective Motor Control is a Clinical Correlate of Brain Motor Tract Impairment in Children with Spastic Bilateral Cerebral Palsy | 2021 |  | Yes |
| 383 | Improved Myelination following Camp Leg Power, a Selective Motor Control Intervention for Children with Spastic Bilateral Cerebral Palsy: A Diffusion Tensor MRI Study | 2023 | No correlation analysis | No |
| 384 | Early prediction of unilateral cerebral palsy in infants at risk: MRI versus the hand assessment for infants | 2020 | Title and abstract clearly do not meet inclusion criteria | No |
| 385 | White and gray matter alterations in adults with Niemann-Pick disease type C: a cross-sectional study | 2010 | Title and abstract clearly do not meet inclusion criteria | No |
| 386 | Effects of three kinds of head acupuncture therapies on regulation of brain microenvironment and rehabilitation of nerve function in rats with cerebral palsy | 2021 | Title and abstract clearly do not meet inclusion criteria | No |
| 387 | A novel form of autosomal recessive hereditary spastic paraplegia caused by a new SPG7 mutation | 2007 | Title and abstract clearly do not meet inclusion criteria | No |
| 388 | [The diagnosis of neurodegenerative disorders based on clinical and pathological findings using an MRI approach] | 2011 | Title and abstract clearly do not meet inclusion criteria | No |
| 389 | A Novel Magnetic Resonance Imaging Score Predicts Neurodevelopmental Outcome After Perinatal Asphyxia and Therapeutic Hypothermia | 2018 | Title and abstract clearly do not meet inclusion criteria | No |
| 390 | Quantification and Monitoring of the Effect of Botulinum Toxin A on Paretic Calf Muscles of Children With Cerebral Palsy With MRI: A Preliminary Study | 2021 | Title and abstract clearly do not meet inclusion criteria | No |
| 391 | Understanding the relationship between brain and upper limb function in children with unilateral motor impairments: A multimodal approach | 2018 |  | Yes |
| 392 | [Isolated cranial nerve palsy secondary to carotid dissection] | 2003 | Title and abstract clearly do not meet inclusion criteria | No |
| 393 | Disrupted thalamocortical connectivity in PSP: a resting-state fMRI, DTI, and VBM study | 2011 | Title and abstract clearly do not meet inclusion criteria | No |
| 394 | Impact of stroke volume on motor outcome in neonatal arterial ischemic stroke | 2020 | Title and abstract clearly do not meet inclusion criteria | No |
| 395 | Factors associated with MRI success in children cooled for neonatal encephalopathy and controls | 2023 | Title and abstract clearly do not meet inclusion criteria | No |
| 396 | Diffusion tensor imaging of Parkinson's disease, multiple system atrophy and progressive supranuclear palsy: a tract-based spatial statistics study | 2014 | Title and abstract clearly do not meet inclusion criteria | No |
| 397 | Cerebellar growth, volume and diffusivity in children cooled for neonatal encephalopathy without cerebral palsy | 2023 | Title and abstract clearly do not meet inclusion criteria | No |
| 398 | The plasticity of the corticospinal tract in children with obstetric brachial plexus palsy after Botulinum Toxin A treatment | 2018 | Title and abstract clearly do not meet inclusion criteria | No |
| 399 | [Efficacy and mechanism of scalp acupuncture for spastic cerebral palsy] | 2023 | Title and abstract clearly do not meet inclusion criteria | No |
| 400 | [Developmental outcomes of very preterm and extremely preterm infants at the 12 months corrected age] | 2023 | Title and abstract clearly do not meet inclusion criteria | No |
| 401 | Isolated Medial Longitudinal Fasciculus Midbrain Infarction Mimicking Medial Rectus Paralysis | 2021 | Title and abstract clearly do not meet inclusion criteria | No |
| 402 | Isolated Medial Rectus Palsy: Rare Presentation of Mesencephalon Infarction | 2017 | Title and abstract clearly do not meet inclusion criteria | No |
| 403 | Impaired glymphatic system revealed by DTI-ALPS in cerebral palsy due to periventricular leukomalacia: relation with brain lesion burden and hand dysfunction | 2023 |  | Yes |
| 404 | Anatomical characterization of athetotic and spastic cerebral palsy using an atlas-based analysis | 2013 | Title and abstract clearly do not meet inclusion criteria | No |
| 405 | Athetotic and spastic cerebral palsy: anatomic characterization based on diffusion-tensor imaging | 2011 | Title and abstract clearly do not meet inclusion criteria | No |
| 406 | Symmetrical central tegmental tract (CTT) hyperintense lesions on magnetic resonance imaging in children | 2009 | Title and abstract clearly do not meet inclusion criteria | No |
| 407 | Quantitative diffusion tensor tractography of the motor and sensory tract in children with cerebral palsy | 2010 | No correlation analysis | No |
| 408 | Cerebral air embolism after removal of internal jugular venous catheter: A case report | 2009 | Title and abstract clearly do not meet inclusion criteria | No |
| 409 | Changes of White Matter Diffusion Anisotropy in Response to a 6-Week iPad Application-Based Occupational Therapy Intervention in Children with Surgically Treated Hydrocephalus: A Pilot Study | 2016 | Title and abstract clearly do not meet inclusion criteria | No |
| 410 | White matter and cortical changes in atypical parkinsonisms: A multimodal quantitative MR study | 2017 | Title and abstract clearly do not meet inclusion criteria | No |
| 411 | The safety and efficacy of umbilical cord blood mononuclear cells in individuals with spastic cerebral palsy: a randomized double-blind sham-controlled clinical trial | 2022 | Title and abstract clearly do not meet inclusion criteria | No |
| 412 | DTI Tract-Based Quantitative Susceptibility Mapping: An Initial Feasibility Study to Investigate the Potential Role of Myelination in Brain Connectivity Change in Cerebral Palsy Patients During Autologous Cord Blood Cell Therapy Using a Rotationally-Invariant Quantitative Measure | 2021 | Title and abstract clearly do not meet inclusion criteria | No |
| 413 | Changes of Structural Brain Network Following Repetitive Transcranial Magnetic Stimulation in Children With Bilateral Spastic Cerebral Palsy: A Diffusion Tensor Imaging Study | 2020 | No correlation analysis | No |
| 414 | Progression of Microstructural Degeneration in Progressive Supranuclear Palsy and Corticobasal Syndrome: A Longitudinal Diffusion Tensor Imaging Study | 2016 | Title and abstract clearly do not meet inclusion criteria | No |
| 415 | Score for neonatal acute physiology-II and neonatal pain predict corticospinal tract development in premature newborns | 2013 | Non-CP | No |
| From Cochrane Library | | | | |
| 416 | Effectiveness of GAME (Goals Activity Motor Enrichment) for infants at high risk of cerebral palsy | 2017 | Title and abstract clearly do not meet inclusion criteria | No |
| 417 | HABIT-ILE: a randomised trial of hand arm bimanual intensive training including lower extremity training for children with bilateral cerebral palsy | 2018 | No correlation analysis | No |
| 418 | Clinical and imaging outcomes after intrathecal injection of umbilical cord tissue mesenchymal stem cells in cerebral palsy: a randomized double-blind sham-controlled clinical trial | 2021 | Duplicate Article | No |
| 419 | HABIT-ILE improves corticospinal tract integrity in children with bilateral cerebral palsy | 2022 | No correlation analysis | No |
| 420 | Phase II Randomized Controlled Trial of Constraint-Induced Movement Therapy in Multiple Sclerosis. Part 2: effect on White Matter Integrity | 2018 | Title and abstract clearly do not meet inclusion criteria | No |
| 421 | To what extent can motor skill training improve the structural connectivity of the corticospinal tract fibers in children with cerebral palsy? | 2020 | Title and abstract clearly do not meet inclusion criteria | No |
| 422 | Botulinum toxin type A in the treatment of lower limb spasticity in children with cerebral palsy | 2019 | Title and abstract clearly do not meet inclusion criteria | No |
| 423 | Comparative assessment of therapeutic response to physiotherapy with or without botulinum toxin injection using diffusion tensor tractography and clinical scores in term diplegic cerebral palsy children | 2013 | Duplicate Article | No |
| 424 | Comparative assessment of therapeutic response to physiotherapy with or without botulinum toxin injection using diffusion tensor tractography and clinical scores in term diplegic cerebral palsy children | 2013 | Duplicate Article | No |
| 425 | Development of an exercise program for children with cerebral palsy following orthopaedic surgery | 2012 | Title and abstract clearly do not meet inclusion criteria | No |
| 426 | Brain structural connectivity increases concurrent with functional improvement: evidence from diffusion tensor MRI in children with cerebral palsy during therapy | 2015 | Duplicate Article | No |
| 427 | Relationship Between Integrity of the Corpus Callosum and Bimanual Coordination in Children With Unilateral Spastic Cerebral Palsy | 2019 | Duplicate Article | No |
| 428 | Diffusion tensor analysis of corpus callosum in progressive supranuclear palsy | 2008 | Duplicate Article | No |
| 429 | Effect of rehabilitation on motor outcomes and brain structure connectivity of children with developmental coordination disorder | 2017 | Title and abstract clearly do not meet inclusion criteria | No |
| 430 | Neuroregenerative potential of intravenous infusion of G-CSF followed by mobilized peripheral blood mononuclear cells in children with cerebral palsy | 2015 | Duplicate Article | No |
| 431 | Neuroplastic cerebral grey and white matter changes following constraint-induced movement therapy for chronic hemiparetic MS: randomised controlled trial | 2015 | Title and abstract clearly do not meet inclusion criteria | No |
| 432 | The Effects of Low Frequency Repetitive Transcranial Magnetic Stimulation on White Matter Structural Connectivity in Children with Cerebral Palsy | 2018 | Duplicate Article | No |
| 433 | Umbilical cord blood therapy potentiated with erythropoietin for children with cerebral palsy: a double-blind, randomized, placebo-controlled trial | 2013 | Duplicate Article | No |
| 434 | Potentiation of cord blood cell therapy with erythropoietin for children with CP: a 2?×?2 factorial randomized placebo-controlled trial | 2020 | Title and abstract clearly do not meet inclusion criteria | No |
| 435 | MRI and Neurodevelopment in Preterm Infants Following Administration of High-Dose Caffeine | 2008 | Title and abstract clearly do not meet inclusion criteria | No |
| 436 | Efficacy of Allogeneic Umbilical Cord Derived Hematopoietic and Mesenchymal Stem Cells in Cerebral Palsy | 2019 | Title and abstract clearly do not meet inclusion criteria | No |
| 437 | Is it possible to predict the effect of balance training based on networks in the brain in children with cerebral palsy? | 2016 | Title and abstract clearly do not meet inclusion criteria | No |
| 438 | Speech therapy for children with dysarthria acquired before three years of age | 2016 | Title and abstract clearly do not meet inclusion criteria | No |
| 439 | Effect of antenatal magnesium sulphate on MRI biomarkers of white matter development at term equivalent age: the magnum study | 2020 | Duplicate Article | No |
| 440 | Effect of antenatal magnesium sulphate on MRI biomarkers of white matter development at term equivalent age: the MagNUM Study | 2022 | Duplicate Article | No |
| 441 | Neuroregenerative potential of intravenous G-CSF and autologous peripheral blood stem cells in children with cerebral palsy: a randomized, double-blind, cross-over study | 2017 | Duplicate Article | No |
| 442 | Prognostic models for predicting clinical disease progression, worsening and activity in people with multiple sclerosis | 2023 | Title and abstract clearly do not meet inclusion criteria | No |
| 443 | Diffusion tensor imaging study of the response to constraint-induced movement therapy of children with hemiparetic cerebral palsy and adults with chronic stroke | 2014 | Duplicate Article | No |
| 444 | Improvement in upper extremity function in children with unilateral spastic cerebral palsy after intensive training correlates with interhemispheric connectivity | 2018 | No correlation analysis | No |
| 445 | Treatment-induced plasticity in cerebral palsy: a diffusion tensor imaging study | 2008 | Duplicate Article | No |
| 446 | Efficacy and mechanism of scalp acupuncture for spastic cerebral palsy | 2023 | Title and abstract clearly do not meet inclusion criteria | No |
| 447 | The safety and efficacy of umbilical cord blood mononuclear cells in individuals with spastic cerebral palsy: a randomized double-blind sham-controlled clinical trial | 2022 | Title and abstract clearly do not meet inclusion criteria | No |
| 448 | DTI Tract-Based Quantitative Susceptibility Mapping: an Initial Feasibility Study to Investigate the Potential Role of Myelination in Brain Connectivity Change in Cerebral Palsy Patients During Autologous Cord Blood Cell Therapy Using a Rotationally-Invariant Quantitative Measure | 2021 | Title and abstract clearly do not meet inclusion criteria | No |
| From Embase | | | | |
| 449 | Erratum to Sensory and motor deficits in children with cerebral palsy born preterm correlate with diffusion tensor imaging abnormalities in thalamocortical pathways [DMCN (2009), 51, (697 704)] | 2009 | No correlation analysis | No |
| 450 | Diffusion tensor imaging of periventricular leukomalacia - Initial experience | 2014 | Title and abstract clearly do not meet inclusion criteria | No |
| 451 | Early Imaging and Adverse Neurodevelopmental Outcome in Asphyxiated Newborns Treated With Hypothermia | 2017 | Duplicate Article | No |
| 452 | A connectome-based approach to assess motor outcome after neonatal arterial ischemic stroke | 2021 | Duplicate Article | No |
| 453 | White matter organization and response to constraint induced therapy in children with cerebral palsy | 2010 | No correlation analysis | No |
| 454 | Neurodevelopmental outcomes of preterm infants | 2008 | Title and abstract clearly do not meet inclusion criteria | No |
| 455 | Preterm birth: Transition to adulthood | 2010 | Title and abstract clearly do not meet inclusion criteria | No |
| 456 | The Role of White Matter in the Neural Control of Swallowing: A Systematic Review | 2021 | review | No |
| 457 | Effects of Piano Training in Unilateral Cerebral Palsy Using Probabilistic and Deterministic Tractography: A Case Report | 2021 | Duplicate Article | No |
| 458 | Clinical and imaging outcomes after intrathecal injection of umbilical cord tissue mesenchymal stem cells in cerebral palsy: a randomized double-blind sham-controlled clinical trial | 2021 | Duplicate Article | No |
| 459 | Improvement of the integrity of upper and lower extremity fibers of the corticospinal tract in children with bilateral cerebral palsy following intensive therapy | 2022 | Title and abstract clearly do not meet inclusion criteria | No |
| 460 | HABIT-ILE improves corticospinal tract integrity in children with bilateral cerebral palsy | 2022 | Duplicate Article | No |
| 461 | Corticospinal tract (re)organization in children with bilateral cerebral palsy correlates with upper and lower extremities function | 2021 | Title and abstract clearly do not meet inclusion criteria | No |
| 462 | Impact of early brain lesions on the retrogeniculate visual pathway in children with cerebral palsy: A diffusion tensor imaging study | 2019 |  | Yes |
| 463 | Impact of early brain lesions on the optic radiations in children with cerebral palsy | 2022 | Duplicate Article | No |
| 464 | Localization of Basal Ganglia and Thalamic Damage in Dyskinetic Cerebral Palsy | 2016 | Title and abstract clearly do not meet inclusion criteria | No |
| 465 | Brain lesions in preterm infants: Initial diagnosis and follow-up | 2010 | Duplicate Article | No |
| 466 | The rationale for routine cerebral ultrasound in premature infants | 2015 | Title and abstract clearly do not meet inclusion criteria | No |
| 467 | The effects of hemorrhagic parenchymal infarction on the establishment of sensori-motor structural and functional connectivity in early infancy | 2014 | Duplicate Article | No |
| 468 | Relationship between brain structure on magnetic resonance imaging and motor outcomes in children with cerebral palsy: A systematic review | 2013 | review | No |
| 469 | Whole-brain DTI assessment of white matter damage in children with bilateral cerebral palsy: Evidence of involvement beyond the primary target of the anoxic insult | 2016 | Duplicate Article | No |
| 470 | Whole-brain assessment of white matter damage in children with cerebral palsy: A DTI study | 2015 |  | Yes |
| 471 | Diffusion tensor brain imaging findings at term-equivalent age may predict neurologic abnormalities in low birth weight preterm infants | 2003 | Duplicate Article | No |
| 472 | Impact of anti-gravity locomotion (AlterG) training on structure and function of corticospinal tract and gait in children with cerebral palsy | 2019 | Duplicate Article | No |
| 473 | Assessment of neuroplasticity of corticospinal tract induced by antigravity treadmill (AlterG) in cerebral palsy children | 2018 | Duplicate Article | No |
| 474 | Contribution of altered corticospinal microstructure to gait impairment in children with cerebral palsy | 2021 | Duplicate Article | No |
| 475 | The impact of an anti-gravity treadmill (AlterG) training on walking capacity and corticospinal tract structure in children with cerebral palsy | 2017 | Duplicate Article | No |
| 476 | The impact of AlterG training on balance and structure of vestibulospinal tract in cerebral palsy children | 2018 | Duplicate Article | No |
| 477 | Early predictors of neurodevelopment after perinatal arterial ischemic stroke: a systematic review and meta-analysis | 2023 | review | No |
| 478 | CST recovery in pediatric hemiplegic patients: Diffusion tensor tractography study | 2013 | Duplicate Article | No |
| 479 | A role of diffusion tensor imaging in movement disorder surgery | 2010 | Title and abstract clearly do not meet inclusion criteria | No |
| 480 | Mapping corticospinal tract projection patterns in unilateral cerebral palsy | 2017 | Title and abstract clearly do not meet inclusion criteria | No |
| 481 | Prophylactic maternal N-acetylcysteine in rats prevents maternal inflammation-induced offspring cerebral injury shown on magnetic resonance imaging | 2013 | Duplicate Article | No |
| 482 | Prophylactic maternal N-acetyl-cysteine (NAC) prevents maternal inflammation-induced offspring cerebral injury evident by magnetic resonance imaging (MRI) | 2013 | Duplicate Article | No |
| 483 | Magnesium sulphate (Mg) prevents maternal inflammation induced offspring cerebral injury evident by magnetic resonance imaging (MRI) | 2011 | Title and abstract clearly do not meet inclusion criteria | No |
| 484 | Possibilities of magnetic resonance imaging of the brain in infantile cerebral palsy | 2018 | Title and abstract clearly do not meet inclusion criteria | No |
| 485 | Neuroimaging of White Matter Injury, Intraventricular and Cerebellar Hemorrhage | 2014 | Title and abstract clearly do not meet inclusion criteria | No |
| 486 | Normal imaging in patients with cerebral palsy: What does it tell us? | 2013 | Title and abstract clearly do not meet inclusion criteria | No |
| 487 | MRI in Normal Myelination: A Pictorial Review | 2021 | review | No |
| 488 | Neuroimaging Perspectives of Perinatal Arterial Ischemic Stroke | 2020 | Duplicate Article | No |
| 489 | Changes in corticospinal tract fibers of children with cerebral palsy following motor skill training: A DTI study | 2017 | Title and abstract clearly do not meet inclusion criteria | No |
| 490 | To what extent can motor skill training improve the structural connectivity of the corticospinal tract fibers in children with cerebral palsy? | 2020 | Duplicate Article | No |
| 491 | Motor Skill Training May Restore Impaired Corticospinal Tract Fibers in Children With Cerebral Palsy | 2020 | Duplicate Article | No |
| 492 | Can motor skill learning restore impaired corticospinal tract fibers in children with unilateral cerebral palsy? A DTI study | 2016 | Title and abstract clearly do not meet inclusion criteria | No |
| 493 | Capturing neuroplastic changes after bimanual intensive rehabilitation in children with unilateral spastic cerebral palsy: A combined DTI, TMS and fMRI pilot study | 2015 | Duplicate Article | No |
| 494 | Corticospinal dysgenesis and upper-limb deficits in congenital hemiplegia: A diffusion tensor imaging study | 2007 | No correlation analysis | No |
| 495 | Periventricular leucomalacia: A review | 2004 | Title and abstract clearly do not meet inclusion criteria | No |
| 496 | Hypoglycaemia and neonatal brain injury | 2013 | Non-CP | No |
| 497 | Neural and physiological changes following intensive voice therapy in children with motor speech disorders secondary to cerebral palsy | 2015 | Title and abstract clearly do not meet inclusion criteria | No |
| 498 | A new framework for analysis of three-dimensional shape and architecture of human skeletal muscles from in vivo imaging data | 2022 | Duplicate Article | No |
| 499 | COMBIT: Protocol of a randomised comparison trial of COMbined modified constraint induced movement therapy and bimanual intensive training with distributed model of standard upper limb rehabilitation in children with congenital hemiplegia | 2013 | Duplicate Article | No |
| 500 | Diffusion Tensor Imaging Abnormalities in the Cerebral White Matter Correlate with Sex-Dependent Neurobehavioral Deficits in Adult Mice with Neonatal Ischemia | 2016 | Non-CP | No |
| 501 | Cerebral palsy of the elbow and forearm | 2014 | Title and abstract clearly do not meet inclusion criteria | No |
| 502 | Joubert syndrome: Findings at conventional magnetic resonance image and at diffusion tensor imaging | 2012 | Duplicate Article | No |
| 503 | Etiology of impaired selective motor control: Emerging evidence and its implications for research and treatment in cerebral palsy | 2014 | Duplicate Article | No |
| 504 | Analysis of near-term white matter microstructure to predict gait in preterm toddlers: A multivariate linear regression model using forward feature selection optimized with cross validation | 2017 | No correlation analysis | No |
| 505 | Neonatal microstructural development of the corpus callosum and gait temporal-spatial parameters in very low birth weight preterm children at 18-20 months: A diffusion imaging study | 2013 | Non-CP | No |
| 506 | Compound Heterozygous Variants in ROBO1 Cause a Neurodevelopmental Disorder With Absence of Transverse Pontine Fibers and Thinning of the Anterior Commissure and Corpus Callosum | 2017 | Title and abstract clearly do not meet inclusion criteria | No |
| 507 | Regional vulnerability of longitudinal cortical association connectivity: Associated with structural network topology alterations in preterm children with cerebral palsy | 2015 | Duplicate Article | No |
| 508 | Brain Outcomes in Runted Piglets: A Translational Model of Fetal Growth Restriction | 2022 | Title and abstract clearly do not meet inclusion criteria | No |
| 509 | Degenerative changes of the corticospinal tract in pediatric patients showing deteriorated motor function: A diffusion tensor tractography study | 2015 | Duplicate Article | No |
| 510 | Diffusion tensor imaging demonstrated radiologic differences between diplegic and quadriplegic cerebral palsy | 2012 | Duplicate Article | No |
| 511 | Comparative assessment of therapeutic response to physiotherapy with or without botulinum toxin injection using diffusion tensor tractography and clinical scores in term diplegic cerebral palsy children | 2013 | Duplicate Article | No |
| 512 | Chorioamnionitis in the Pathogenesis of Brain Injury in Preterm Infants | 2014 | Duplicate Article | No |
| 513 | Magnetic resonance imaging in the encephalopathic term newborn | 2014 | Title and abstract clearly do not meet inclusion criteria | No |
| 514 | In utero methadone exposure permanently alters anatomical and functional connectivity: A preclinical evaluation | 2023 | Duplicate Article | No |
| 515 | Diffusion tensor imaging-demonstrated differences between hemiplegic and diplegic cerebral palsy with symmetric periventricular leukomalacia | 2013 | Duplicate Article | No |
| 516 | Three-dimensional architecture of the medial gastrocnemius muscle in human infants in vivo | 2022 | Title and abstract clearly do not meet inclusion criteria | No |
| 517 | Three-dimensional skeletal muscle architecture in the lower legs of living human infants | 2023 | Duplicate Article | No |
| 518 | Consequences of intraventricular hemorrhage in a rabbit pup model | 2009 | Duplicate Article | No |
| 519 | New understanding of adolescent brain development: Relevance to transitional healthcare for young people with long term conditions | 2013 | Title and abstract clearly do not meet inclusion criteria | No |
| 520 | Full Activation Profiles and Integrity of Corticospinal Pathways in Adults With Bilateral Spastic Cerebral Palsy | 2019 | Duplicate Article | No |
| 521 | The influence of early nutrition on brain growth and neurodevelopment in extremely preterm babies: A narrative review | 2019 | review | No |
| 522 | Imaging Developmental and Interventional Plasticity following Perinatal Stroke | 2021 | Duplicate Article | No |
| 523 | Little brain, big expectations | 2020 | Title and abstract clearly do not meet inclusion criteria | No |
| 524 | Safety and Outcomes of Dentate Nucleus Deep Brain Stimulation for Cerebellar Ataxia | 2022 | Title and abstract clearly do not meet inclusion criteria | No |
| 525 | Muscle architecture in children with cerebral palsy and ankle contractures: an investigation using diffusion tensor imaging | 2019 | Duplicate Article | No |
| 526 | Functional and structural brain connectivity in children with bilateral cerebral palsy compared to age-related controls and in response to intensive rapid-reciprocal leg training | 2022 | Duplicate Article | No |
| 527 | Tractography of white-matter tracts in very preterm infants: A 2-year follow-up study | 2013 | Duplicate Article | No |
| 528 | Tractography of white matter tracts in very preterm infants-A 2-year follow-up study | 2013 | Title and abstract clearly do not meet inclusion criteria | No |
| 529 | Brain imaging and prognosis of neurodevelopmental outcome in preterm infants | 2015 | Title and abstract clearly do not meet inclusion criteria | No |
| 530 | Patterns of neonatal hypoxic-ischaemic brain injury | 2010 | Non-CP | No |
| 531 | Value of sequential MRI in preterm infants | 2013 | Title and abstract clearly do not meet inclusion criteria | No |
| 532 | Diffusion tensor imaging in a patient with cerebral palsy and hypersomnia | 2013 | No correlation analysis | No |
| 533 | Diffusion tensor imaging in a patient with cerebral palsy and hypersomnia | 2014 | Duplicate Article | No |
| 534 | Very preterm children at risk for developmental coordination disorder have brain alterations in motor areas | 2019 | Duplicate Article | No |
| 535 | Earlier markers for cerebral palsy and clinical research in premature infants | 2007 | Title and abstract clearly do not meet inclusion criteria | No |
| 536 | Magnetic resonance imaging of the preterm infant brain | 2014 | Title and abstract clearly do not meet inclusion criteria | No |
| 537 | Magnetic Resonance Imaging of the Preterm Infant Brain | 2014 | Duplicate Article | No |
| 538 | Concurrent decrease of brain white matter tracts' thicknesses and fractional anisotropy after antenatal hypoxia-ischemia detected with tract-based spatial statistics analysis | 2017 | Duplicate Article | No |
| 539 | White matter injury correlates with hypertonia in an animal model of cerebral palsy | 2007 | Duplicate Article | No |
| 540 | Unmyelinated axon loss with postnatal hypertonia after fetal hypoxia | 2014 | Duplicate Article | No |
| 541 | Spinal cord injury in hypertonic newborns after antenatal hypoxia-ischemia in a rabbit model of cerebral palsy | 2017 | Duplicate Article | No |
| 542 | Developmental changes in diffusion anisotropy coincide with immature oligodendrocyte progression and maturation of compound action potential | 2005 | Duplicate Article | No |
| 543 | Occult spastic diplegic cerebral palsy recognition using efficient machine learning for big data and structural connectivity abnormalities analysis | 2018 | Title and abstract clearly do not meet inclusion criteria | No |
| 544 | Brain development in infants born preterm: Looking beyond injury | 2013 | Title and abstract clearly do not meet inclusion criteria | No |
| 545 | Can diffusion tensor imaging predict cerebral palsy in term neonates with hypoxic ischemic encephalopathy? | 2019 | No correlation analysis | No |
| 546 | The relationship between white matter integrity and motor function in spastic diplegic cerebral palsy with and without periventricular leukomalacia | 2012 | Title and abstract clearly do not meet inclusion criteria | No |
| 547 | Diffuse reduction of white matter connectivity in cerebral palsy with specific vulnerability of long range fiber tracts | 2013 | Duplicate Article | No |
| 548 | Brain structural connectivity increases concurrent with functional improvement: Evidence from diffusion tensor MRI in children with cerebral palsy during therapy | 2015 | Duplicate Article | No |
| 549 | The role of diffusion-tensor magnetic resonance imaging and tractography in the diagnosis of structural brain disorders in children with cerebral palsy | 2016 | Title and abstract clearly do not meet inclusion criteria | No |
| 550 | Therapeutic potency of cord blood stem cells in patients with cerebral palsy: A systemic literature review | 2018 | review | No |
| 551 | Caveats in diffusion tensor imaging interpretation | 2010 | Duplicate Article | No |
| 552 | Quantitative analysis of brain pathology based on MRI and brain atlases-Applications for cerebral palsy | 2011 | Title and abstract clearly do not meet inclusion criteria | No |
| 553 | Atlas-based analysis of neurodevelopment from infancy to adulthood using diffusion tensor imaging and applications for automated abnormality detection | 2010 | No correlation analysis | No |
| 554 | Evidence of axonal injury in a neonatal mouse model of periventricular leukomalacia | 2010 | Title and abstract clearly do not meet inclusion criteria | No |
| 555 | Preterm birth and developmental problems in the preschool age. Part I: Minor motor problems | 2012 | Title and abstract clearly do not meet inclusion criteria | No |
| 556 | Inhibition of the sodium-hydrogen exchanger after neonatal hypoxia-ischemia results in sparing of white matter injury and improved memory and learning | 2011 | Non-CP | No |
| 557 | Relationship between sensorimotor tracts and sensorimotor function in children with unilateral spastic cerebral palsy | 2018 |  | Yes |
| 558 | Setting Up a Pediatric Deep Brain Stimulation Unit | 2022 | Title and abstract clearly do not meet inclusion criteria | No |
| 559 | Are We Getting It Right? A Scoping Review of Outcomes Reported in Cell Therapy Clinical Studies for Cerebral Palsy | 2022 | review | No |
| 560 | White matter changes associated with cognitive visual dysfunctions in children with cerebral palsy: A diffusion tensor imaging study | 2018 | Duplicate Article | No |
| 561 | Identifying Effective Treatments for Dystonia in Patients With Cerebral Palsy A Precision Therapeutics Approach | 2023 | Title and abstract clearly do not meet inclusion criteria | No |
| 562 | Are diffusion tensor imaging and tractography useful for predicting clinical outcome after childhood stroke? A case study | 2010 | No correlation analysis | No |
| 563 | Severe retinopathy of prematurity predicts delayed white matter maturation and poorer neurodevelopment | 2017 | Title and abstract clearly do not meet inclusion criteria | No |
| 564 | Spatial distribution and quantitative tract-based study of chronic white matter lesions in dyskinetic type of cerebral palsy secondary to neonatal hypoxic encephalopathy | 2014 | Non-CP | No |
| 565 | Impaired voluntary movement control and its rehabilitation in cerebral palsy | 2016 | Title and abstract clearly do not meet inclusion criteria | No |
| 566 | HABIT+tDCS: a study protocol of a randomised controlled trial (RCT) investigating the synergistic efficacy of hand-arm bimanual intensive therapy (HABIT) plus targeted non-invasive brain stimulation to improve upper extremity function in school-age children with unilateral cerebral palsy | 2022 | Title and abstract clearly do not meet inclusion criteria | No |
| 567 | The magic of movement: Neural plasticity and influence on outcomes in childhood motor disorders | 2014 | Title and abstract clearly do not meet inclusion criteria | No |
| 568 | White Matter Changes in Children with Hemiplegia Following Constraint-induced Movement Therapy | 2022 | Title and abstract clearly do not meet inclusion criteria | No |
| 569 | Treatment of Central Paralysis of Upper Extremity Using Contralateral C7 Nerve Transfer via Posterior Spinal Route | 2019 | Title and abstract clearly do not meet inclusion criteria | No |
| 570 | Effect of sensory and motor connectivity on hand function in pediatric hemiplegia | 2017 | Duplicate Article | No |
| 571 | Neural Correlates of Impaired Grasp Function in Children with Unilateral Spastic Cerebral Palsy | 2023 | Duplicate Article | No |
| 572 | Reorganization of thalamo-cortical fibers underlies sparing of visual fields in preterm unilateral periventricular damage | 2010 | Title and abstract clearly do not meet inclusion criteria | No |
| 573 | Plasticity of the visual system after early brain damage | 2010 | Title and abstract clearly do not meet inclusion criteria | No |
| 574 | Soleus muscle architecture indicates impaired plantarflexor strength in cerebral palsy | 2018 | Title and abstract clearly do not meet inclusion criteria | No |
| 575 | Visualization of language and pyramidal tracts in young patients with severe cerebral palsy: A diffusion tensor imaging study | 2012 | No correlation analysis | No |
| 576 | Language comprehension in young people with severe cerebral palsy in relation to language tracts: A diffusion tensor imaging study | 2013 | Duplicate Article | No |
| 577 | Magnetic resonance imaging and developmental outcome following preterm birth: Review of current evidence | 2008 | review | No |
| 578 | Pediatric unilateral spatial neglect: A systematic review | 2021 | review | No |
| 579 | Cerebellar peduncle injury predicts motor impairments in preterm infants: A quantitative tractography study at term-equivalent age | 2018 | Duplicate Article | No |
| 580 | Comparison of descending motor pathways between prenatal and perinatal unilateral brain injuries | 2014 | Title and abstract clearly do not meet inclusion criteria | No |
| 581 | Brain magnetic resonance imaging and outcome after hypoxic ischaemic encephalopathy | 2016 | Title and abstract clearly do not meet inclusion criteria | No |
| 582 | Corticospinal tract DTI interrogations and motor outcome in perinatal stroke | 2012 | Title and abstract clearly do not meet inclusion criteria | No |
| 583 | Corticospinal tract integrity and motor outcomes using diffusion tensor imaging in perinatal stroke | 2012 | No correlation analysis | No |
| 584 | Segmental Diffusion Properties of the Corticospinal Tract and Motor Outcome in Hemiparetic Children with Perinatal Stroke | 2017 | Duplicate Article | No |
| 585 | Predicting motor outcome using DTI quantification of corticospinal tract integrity after perinatal stroke | 2014 | Title and abstract clearly do not meet inclusion criteria | No |
| 586 | White matter alterations and their associations with motor function in young adults born preterm with very low birth weight | 2018 | Duplicate Article | No |
| 587 | Neuroimaging in cerebral palsy: Patterns of brain dysgenesis and injury | 2005 | Duplicate Article | No |
| 588 | Pathogenesis, neuroimaging and management in children with cerebral palsy born preterm | 2010 | Title and abstract clearly do not meet inclusion criteria | No |
| 589 | Diffusion tensor imaging of periventricular leukomalacia shows affected sensory cortex white matter pathways | 2002 | No correlation analysis | No |
| 590 | Sensory and motor deficits in children with cerebral palsy born preterm correlate with diffusion tensor imaging abnormalities in thalamocortical pathways | 2009 | No correlation analysis | No |
| 591 | The relationship between early neuroimaging findings and the outcome of neonatal seizure | 2012 | Non-CP | No |
| 592 | White and gray matter development in human fetal, newborn and pediatric brains | 2006 | Title and abstract clearly do not meet inclusion criteria | No |
| 593 | Relationship Between Integrity of the Corpus Callosum and Bimanual Coordination in Children With Unilateral Spastic Cerebral Palsy | 2019 | Duplicate Article | No |
| 594 | Using diffusion tensor imaging based measurements to predict outcomes of constraint induced movement therapy in children with hemiplegic cerebral palsy | 2017 | No correlation analysis | No |
| 595 | Using diffusion tensor imaging based measurements to predict outcomes of constraint induced movement therapy in children with hemiplegic cerebral palsy | 2017 | Duplicate Article | No |
| 596 | Effect of rehabilitation on motor outcomes and brain structure connectivity of children with developmental coordination disorder | 2017 | Duplicate Article | No |
| 597 | Will a novel semi-quantitative scale for classification of structural brain MRI improve patient care and research in children with cerebral palsy? | 2014 | Title and abstract clearly do not meet inclusion criteria | No |
| 598 | Brain white matter integrity in cerebral palsy: relation with balance control | 2019 | Title and abstract clearly do not meet inclusion criteria | No |
| 599 | Cerebral blood flow and DTI metrics changes in children with cerebral palsy following therapy | 2014 | Title and abstract clearly do not meet inclusion criteria | No |
| 600 | Aberrant pyramidal tract in comparison with pyramidal tract on diffusion tensor tractography: A mini-review | 2017 | review | No |
| 601 | Arcuate fasciculus in young people with severe cerebral palsy: A diffusion tensor imaging study | 2011 |  | Yes |
| 602 | Early diagnosis of spastic cerebral palsy in infants with periventricular white matter injury using diffusion tensor imaging | 2019 | Duplicate Article | No |
| 603 | Specific White Matter Lesions Related to Motor Dysfunction in Spastic Cerebral Palsy: A Meta-analysis of Diffusion Tensor Imaging Studies | 2020 | Duplicate Article | No |
| 604 | Structural network performance for early diagnosis of spastic cerebral palsy in periventricular white matter injury | 2020 | Title and abstract clearly do not meet inclusion criteria | No |
| 605 | Structural network performance for early diagnosis of spastic cerebral palsy in periventricular white matter injury | 2021 | Duplicate Article | No |
| 606 | Correlation between diffusion tensor imaging parameters and classification of gross motor function in infants of periventricular leucomalacia with cerebral palsy | 2014 | No correlation analysis | No |
| 607 | A comparison of microstructural maturational changes of the corpus callosum in preterm and full-term children: A diffusion tensor imaging study | 2012 | Duplicate Article | No |
| 608 | Diffusion tensor imaging of white matter and developmental outcome | 2008 | Duplicate Article | No |
| 609 | Medial lemniscus lesion in pediatric hemiplegic patients without corticospinal tract and posterior thalamic radiation lesion | 2012 | Duplicate Article | No |
| 610 | Prematurity and foetal growth restriction are associated with altered white matter maturation at nine years of age | 2016 | Title and abstract clearly do not meet inclusion criteria | No |
| 611 | Correlation of Different MRI Scoring Systems with Long-Term Cognitive Outcome in Cooled Asphyxiated Newborns | 2023 | Title and abstract clearly do not meet inclusion criteria | No |
| 612 | 45: Microstructural thalamic injury in a rat model of chorioamnionitis: potential avenues for neurorepair | 2019 | Title and abstract clearly do not meet inclusion criteria | No |
| 613 | Neurodevelopmental impairment is associated with altered white matter development in a cohort of school-aged children born very preterm | 2021 | Duplicate Article | No |
| 614 | Neonatal diffusion tensor brain imaging predicts later motor outcome in preterm neonates with white matter abnormalities | 2016 | Duplicate Article | No |
| 615 | Limb Length Discrepancy and Corticospinal Tract Disruption in Hemiplegic Cerebral Palsy | 2022 | Duplicate Article | No |
| 616 | Chronic fetal hypoxia affects axonal maturation in Guinea pigs during development: A longitudinal diffusion tensor imaging and T<inf>2</inf> mapping study | 2015 | No correlation analysis | No |
| 617 | Motor function outcomes of pediatric patients with hemiplegic cerebral palsy after rehabilitation treatment: A diffusion tensor imaging study | 2015 | Duplicate Article | No |
| 618 | Modeling developmental plasticity after perinatal stroke: Defining central therapeutic targets in cerebral palsy | 2013 | Duplicate Article | No |
| 619 | Stroke in the fetus and neonate | 2006 | Title and abstract clearly do not meet inclusion criteria | No |
| 620 | Targeted neuroimmunomodulatory therapies for functional improvement in cerebral palsy | 2021 | Title and abstract clearly do not meet inclusion criteria | No |
| 621 | The importance of MRI tractography in the examination of adult patients with cerebral palsy | 2020 | Duplicate Article | No |
| 622 | Mutations in gamma adducin are associated with inherited cerebral palsy | 2013 | Title and abstract clearly do not meet inclusion criteria | No |
| 623 | Sensory tractography and robot-quantified proprioception in hemiparetic children with perinatal stroke | 2017 | Duplicate Article | No |
| 624 | Corticospinal tract diffusion properties and robotic visually guided reaching in children with hemiparetic cerebral palsy | 2018 | Duplicate Article | No |
| 625 | Using diffusion tensor imaging as a surrogate to identify corticospinal tract connectivity in children with unilateral spastic cerebral palsy | 2014 | No correlation analysis | No |
| 626 | Using diffusion tensor imaging to identify corticospinal tract projection patterns in children with unilateral spastic cerebral palsy | 2017 | Duplicate Article | No |
| 627 | Changes in diffusion tensor tractographic findings associated with constraint-induced movement therapy in young children with cerebral palsy | 2014 | Duplicate Article | No |
| 628 | The Role of Neuroimaging in Predicting Neurodevelopmental Outcomes of Preterm Neonates | 2014 | Title and abstract clearly do not meet inclusion criteria | No |
| 629 | Corticoreticular tract lesion in children with developmental delay presenting with gait dysfunction and trunk instability | 2017 | Duplicate Article | No |
| 630 | Diffusion tensor imaging is associated with motor outcomes of very preterm born children at 11 years of age | 2020 | Title and abstract clearly do not meet inclusion criteria | No |
| 631 | The MRI-compatible neonatal incubator in practice | 2013 | Non-CP | No |
| 632 | Changes in a cerebellar peduncle lesion in a patient with Dandy-Walker malformation a diffusion tensor imaging study | 2013 | Duplicate Article | No |
| 633 | Analysis of structure–function network decoupling in the brain systems of spastic diplegic cerebral palsy | 2017 | Title and abstract clearly do not meet inclusion criteria | No |
| 634 | Motor pathway injury in patients with periventricular leucomalacia and spastic diplegia | 2011 | Duplicate Article | No |
| 635 | Diffusion tensor imaging with fiber tractography: Assessment of developing brain and aberrant fiber connections in CNS anomalies | 2010 | No correlation analysis | No |
| 636 | Diffusion-tensor MR imaging and fiber tractography: A new method of describing aberrant fiber connections in developmental CNS anomalies | 2005 | Duplicate Article | No |
| 637 | Safety and feasibility of countering neurological impairment by intravenous administration of autologous cord blood in cerebral palsy | 2012 | Duplicate Article | No |
| 638 | Neuroregenerative potential of intravenous infusion of G-CSF followed by mobilized peripheral blood mononuclear cells in children with cerebral palsy | 2015 | Duplicate Article | No |
| 639 | Advanced fiber tracking in early acquired brain injury causing cerebral palsy | 2015 | Duplicate Article | No |
| 640 | Advanced fiber tractography in cerebral palsy suggests primary and secondary microstructural changes along the corticospinal tract and somatosensory projections | 2013 | Title and abstract clearly do not meet inclusion criteria | No |
| 641 | Brain white matter network measures for cerebral palsy | 2018 | Title and abstract clearly do not meet inclusion criteria | No |
| 642 | Research on brain white matter network in cerebral palsy infant | 2017 | Duplicate Article | No |
| 643 | In-depth analysis reveals complex molecular aetiology in a cohort of idiopathic cerebral palsy | 2022 | Title and abstract clearly do not meet inclusion criteria | No |
| 644 | Structure of brain grey and white matter in infants with spastic cerebral palsy and periventricular white matter injury | 2023 | Duplicate Article | No |
| 645 | Associations between central nervous system disorder and congenital esotropia | 2013 | Title and abstract clearly do not meet inclusion criteria | No |
| 646 | Peak width of skeletonised DTI Metrics as a marker for white matter disruption in dyskinetic cerebral palsy | 2021 | Title and abstract clearly do not meet inclusion criteria | No |
| 647 | Outcome following pallidal DBS correlates with white matter microstructural integrity: A diffusion tensor imaging study | 2014 | Title and abstract clearly do not meet inclusion criteria | No |
| 648 | Whether Erythropoietin can be a Neuroprotective Agent against Premature Brain Injury: Cellular Mechanisms and Clinical Efficacy | 2022 | Title and abstract clearly do not meet inclusion criteria | No |
| 649 | Use of advanced neuroimaging and non invasive brain stimulation techniques to predict hand function ability in hemiplegic cerebral palsy | 2011 |  | Yes |
| 650 | Does motor deficit in children with cerebral palsy correlate with diffusion tensor metrics abnormalities in thalamocortical pathways? | 2021 | No correlation analysis | No |
| 651 | Constraint-induced movement therapy improves neural processing efficiency and neurobehavioral function in children with hemiparetic cerebral palsy | 2011 | Title and abstract clearly do not meet inclusion criteria | No |
| 652 | Resting state and diffusion neuroimaging predictors of clinical improvements following constraint-induced movement therapy in children with hemiplegic cerebral palsy | 2015 | Duplicate Article | No |
| 653 | Neuroplastic cerebral grey and white matter changes following constraint-induced movement therapy for chronic hemiparetic MS: Randomised controlled trial | 2015 | Title and abstract clearly do not meet inclusion criteria | No |
| 654 | Neurodevelopmental outcome in children with congenital heart disease | 2013 | Title and abstract clearly do not meet inclusion criteria | No |
| 655 | The Effects of Low Frequency Repetitive Transcranial Magnetic Stimulation on White Matter Structural Connectivity in Children with Cerebral Palsy | 2018 | Duplicate Article | No |
| 656 | Neonatal neurobehavior after therapeutic hypothermia for hypoxic ischemic encephalopathy | 2015 | Duplicate Article | No |
| 657 | Magnetic resonance imaging-Insights into brain injury and outcomes in premature infants | 2009 | Duplicate Article | No |
| 658 | Understanding Brain Injury and Neurodevelopmental Disabilities in the Preterm Infant: The Evolving Role of Advanced Magnetic Resonance Imaging | 2010 | Title and abstract clearly do not meet inclusion criteria | No |
| 659 | Long-Term Neuropathological Changes Associated with Cerebral Palsy in a Nonhuman Primate Model of Hypoxic-Ischemic Encephalopathy | 2017 | Duplicate Article | No |
| 660 | Dystonia in Childhood: How Insights from Paediatric Research Enrich the Network Theory of Dystonia | 2023 | Title and abstract clearly do not meet inclusion criteria | No |
| 661 | Assessment of motor and sensory pathways of the brain using diffusion-tensor tractography in children with cerebral palsy | 2014 | Duplicate Article | No |
| 662 | Neonatal Functional and Structural Connectivity Are Associated with Cerebral Palsy at Two Years of Age | 2020 | Duplicate Article | No |
| 663 | Macrostructural and Microstructural Brain Lesions Relate to Gait Pathology in Children with Cerebral Palsy | 2016 | Duplicate Article | No |
| 664 | From selective vulnerability to connectivity: insights from newborn brain imaging | 2009 | Title and abstract clearly do not meet inclusion criteria | No |
| 665 | Umbilical cord blood therapy potentiated with erythropoietin for children with cerebral palsy: A double-blind, randomized, placebo-controlled trial | 2013 | Duplicate Article | No |
| 666 | Potentiation of cord blood cell therapy with erythropoietin for children with CP: a 2 × 2 factorial randomized placebo-controlled trial | 2020 | Title and abstract clearly do not meet inclusion criteria | No |
| 667 | Reliability of fractional anisotropy measurement for children with cerebral palsy | 2014 | Duplicate Article | No |
| 668 | Modifiable risk factors for preterm brain injury | 2014 | Title and abstract clearly do not meet inclusion criteria | No |
| 669 | Cerebellar peduncles injury in preterm infants with cerebral palsy as detected by quantitative tractography | 2011 | Title and abstract clearly do not meet inclusion criteria | No |
| 670 | The Role of the Corpus Callosum in Pediatric Dysphagia: Preliminary Findings from a Diffusion Tensor Imaging Study in Children with Unilateral Spastic Cerebral Palsy | 2017 | Duplicate Article | No |
| 671 | Fiber-tracking techniques can predict the degree of neurologic impairment for periventricular leukomalacia | 2008 | Duplicate Article | No |
| 672 | Long-term Outcome of Preterm Infants and the Role of Neuroimaging | 2009 | Title and abstract clearly do not meet inclusion criteria | No |
| 673 | Diffusion tensor imaging in children with periventricular leukomalacia: Variability of injuries to white matter tracts | 2007 | No correlation analysis | No |
| 674 | Diffusion tensor imaging on teenagers, born at term with moderate hypoxic-ischemic encephalopathy | 2005 | Duplicate Article | No |
| 675 | Neurite orientation dispersion and density imaging quantifies corticospinal tract microstructural organization in children with unilateral cerebral palsy | 2019 | Duplicate Article | No |
| 676 | Impact of Lower Limb Active Movement Training in Individuals With Spastic Type Cerebral Palsy on Neuromuscular Control Outcomes: A Systematic Review | 2020 | review | No |
| 677 | The utility of the fronto-temporal horn ratio on cranial ultrasound in premature newborns: a ventriculomegaly marker | 2021 | Duplicate Article | No |
| 678 | Quantitative evaluation of brain development using anatomical MRI and diffusion tensor imaging | 2013 | Duplicate Article | No |
| 679 | Reprint of "Quantitative evaluation of brain development using anatomical MRI and diffusion tensor imaging" | 2014 | Duplicate Article | No |
| 680 | Diffusion tensor imaging in infants with basal ganglia-thalamic lesions | 2008 | No correlation analysis | No |
| 681 | Diffusion Tensor Imaging in Arginase Deficiency Reveals Damage to Corticospinal Tracts | 2010 | Duplicate Article | No |
| 682 | Single-Institution Comparative Study of Magnetic Resonance–Guided Laser Interstitial Thermal Therapy and Open Corpus Callosotomy | 2023 | Title and abstract clearly do not meet inclusion criteria | No |
| 683 | Reconstruction of the visual ventral stream in children based on DTI fiber tracking | 2009 | Title and abstract clearly do not meet inclusion criteria | No |
| 684 | Visual perception in preterm children: What are we currently measuring? | 2011 | Duplicate Article | No |
| 685 | Neuroimaging biomarkers of preterm brain injury: Toward developing the preterm connectome | 2012 | Duplicate Article | No |
| 686 | Fixel-based analysis reveals alterations is brain microstructure and macrostructure of preterm-born infants at term equivalent age | 2018 | Duplicate Article | No |
| 687 | Advanced diffusion weighted magnetic resonance imaging of the extremely preterm infant's brain | 2014 | Title and abstract clearly do not meet inclusion criteria | No |
| 688 | Brain microstructure and morphology of very preterm-born infants at term equivalent age: Associations with motor and cognitive outcomes at 1 and 2 years | 2020 | Duplicate Article | No |
| 689 | Magnetic resonance diffusion tractography of the preterm infant brain: A systematic review | 2014 | review | No |
| 690 | Cerebral palsy: A lifelong challenge asks for early intervention | 2015 | Title and abstract clearly do not meet inclusion criteria | No |
| 691 | Altered White Matter Connectivity Associated with Intergyral Brain Disorganization in Hemiplegic Cerebral Palsy | 2019 | Duplicate Article | No |
| 692 | Cortical somatosensory reorganization in children with spastic cerebral palsy: A multimodal neuroimaging study | 2014 | Duplicate Article | No |
| 693 | Altered white matter connectivity associated with intergyral brain disorganization in cerebral palsy | 2016 | Title and abstract clearly do not meet inclusion criteria | No |
| 694 | Reorganization of the somatosensory cortex in children with cerebral palsy due to impaired thalamocortical sensory tracts | 2016 | Title and abstract clearly do not meet inclusion criteria | No |
| 695 | Reorganization of the somatosensory cortex in hemiplegic cerebral palsy associated with impaired sensory tracts | 2018 | Duplicate Article | No |
| 696 | Maturation of sensory and motor tracts in children with congenital hemiplegia using diffusion tensor imaging | 2018 | No correlation analysis | No |
| 697 | Maturation of Corticospinal Tracts in Children With Hemiplegic Cerebral Palsy Assessed by Diffusion Tensor Imaging and Transcranial Magnetic Stimulation | 2019 | Duplicate Article | No |
| 698 | Neuroimaging of basal ganglia in neurometabolic diseases in children | 2020 | review | No |
| 699 | Functional and neuroplastic changes after a 2-weeks motor-skill learning intervention in a child with neurodegenerative ataxia: A case study | 2021 | Title and abstract clearly do not meet inclusion criteria | No |
| 700 | Increased GABA-A receptor binding and reduced connectivity at the motor cortex in children with hemiplegic cerebral palsy: A multimodal investigation using 18F-fluoroflumazenil PET, immunohistochemistry, and MR imaging | 2013 | Duplicate Article | No |
| 701 | Neural correlates of developmental coordination disorder | 2013 | Duplicate Article | No |
| 702 | Early motor trajectories in very low birth weight premature infants: Use of diffusion tensor imaging, general movement assessment and the test of infant motor performance | 2013 | No correlation analysis | No |
| 703 | Structural and functional motor development in preterm infants: Feasibility of serial MRI and motor assessment | 2023 | Title and abstract clearly do not meet inclusion criteria | No |
| 704 | Detailed analysis of general movements and functional brain connectivity in preterm infants | 2018 | Title and abstract clearly do not meet inclusion criteria | No |
| 705 | Relationship between brain structure and Cerebral Visual Impairment in children with Cerebral Palsy: A systematic review | 2020 | review | No |
| 706 | Role of diffusion tensor imaging as an independent predictor of cognitive and language development in extremely low-birth-weight infants | 2014 | Duplicate Article | No |
| 707 | Effect of antenatal magnesium sulphate on MRI biomarkers of white matter development at term equivalent age: The magnum study | 2020 | Duplicate Article | No |
| 708 | Effect of antenatal magnesium sulphate on MRI biomarkers of white matter development at term equivalent age: The MagNUM Study | 2022 | Duplicate Article | No |
| 709 | Neuroregenerative potential of intravenous G-CSF and autologous peripheral blood stem cells in children with cerebral palsy: A randomized, double-blind, cross-over study | 2017 | Duplicate Article | No |
| 710 | MR Imaging of hypoxic ischemic | 2013 | Duplicate Article | No |
| 711 | Therapeutic effects of an anti-gravity locomotor training (AlterG) on postural balance and cerebellum structure in children with Cerebral Palsy | 2017 | Duplicate Article | No |
| 712 | Changes in White Matter Integrity following Intensive Voice Treatment (LSVT LOUD?) in Children with Cerebral Palsy and Motor Speech Disorders | 2017 | Duplicate Article | No |
| 713 | Surface-based fMRI-driven diffusion tractography in the presence of significant brain pathology: A study linking structure and function in cerebral palsy | 2016 | Duplicate Article | No |
| 714 | Comparing quantitative tractography metrics of motor and sensory pathways in children with periventricular leukomalacia and different levels of gross motor function | 2012 | Duplicate Article | No |
| 715 | Detection of focal cerebral injury using diffusion tensor magnetic resonance imaging in a boy with becker muscular dystrophy | 2009 | Duplicate Article | No |
| 716 | Diffusion tensor imaging study of the response to constraint-induced movement therapy of children with hemiparetic cerebral palsy and adults with chronic stroke | 2014 | Duplicate Article | No |
| 717 | Communication skills in children aged 6-8?years, without cerebral palsy cooled for neonatal hypoxic-ischemic encephalopathy | 2022 | Duplicate Article | No |
| 718 | Improvement in upper extremity function in children with unilateral spastic cerebral palsy after intensive training correlates with interhemispheric connectivity | 2018 | Duplicate Article | No |
| 719 | Corpus Callosum Integrity Relates to Improvement of Upper-Extremity Function Following Intensive Rehabilitation in Children With Unilateral Spastic Cerebral Palsy | 2021 | Duplicate Article | No |
| 720 | Neonatal erythropoietin mitigates impaired gait, social interaction and diffusion tensor imaging abnormalities in a rat model of prenatal brain injury | 2018 | Duplicate Article | No |
| 721 | Neonatal erythropoietin reversal of a spectrum of chronic deficits correlates with diffusion tensor imaging in a preclinical model of cerebral palsy from prematurity | 2016 | Non-CP | No |
| 722 | Early neurodevelopmental outcomes of extremely preterm infants | 2016 | Title and abstract clearly do not meet inclusion criteria | No |
| 723 | eonatal brain structure on MRI and diffusion tensor imaging, sex, and neurodevelopment in very-low-birthweight preterm children | 2009 | No correlation analysis | No |
| 724 | Neonatal microstructural development of the internal capsule on diffusion tensor imaging correlates with severity of gait and motor deficits | 2007 | No correlation analysis | No |
| 725 | Movement disorders due to bilirubin toxicity | 2015 | Duplicate Article | No |
| 726 | Neonatal physiological correlates of early brain development on MRI and DTI in very-low-birthweight preterm infants | 2014 | Title and abstract clearly do not meet inclusion criteria | No |
| 727 | Neonatal neural correlates, physiological risk factors, and early motor development in very low birth weight preterm children: A diffusion imaging study | 2012 | Title and abstract clearly do not meet inclusion criteria | No |
| 728 | Epidemiology and pathogenesis of stroke in preterm infants: A systematic review | 2022 | review | No |
| 729 | Neonatal DTI early after birth predicts motor outcome in preterm infants with periventricular hemorrhagic infarction | 2015 | Duplicate Article | No |
| 730 | Magnetic resonance imaging in perinatal brain injury: Clinical presentation, lesions and outcome | 2006 | Title and abstract clearly do not meet inclusion criteria | No |
| 731 | Magnetic resonance imaging of white matter diseases of prematurity | 2010 | Duplicate Article | No |
| 732 | Intrauterine endotoxin administration leads to white matter diffusivity changes in newborn rabbits | 2009 | Duplicate Article | No |
| 733 | Soleus muscle weakness in cerebral palsy: Muscle architecture revealed with Diffusion Tensor Imaging | 2019 | Duplicate Article | No |
| 734 | Erratum: Soleus muscle weakness in cerebral palsy: Muscle architecture revealed with Diffusion Tensor Imaging (PLoS ONE (2019) 14:2 (e0205944) DOI: 10.1371/journal.pone.0205944) | 2020 | Duplicate Article | No |
| 735 | Experimental cerebral palsy causes microstructural brain damage in areas associated to motor deficits but no spatial memory impairments in the developing rat | 2021 | Duplicate Article | No |
| 736 | Prediction of cognitive and motor development in preterm infants using forward feature selection and linear regression on nearterm regional white matter microstructure assessed with diffusion tensor imaging | 2016 | No correlation analysis | No |
| 737 | Prediction of neurodevelopment using linear regression model and forward feature selection of near-term regional white matter microstructure in children born preterm | 2016 | No correlation analysis | No |
| 738 | Imaging Predictors of Improvement from a Motor Learning-Based Intervention for Children with Unilateral Cerebral Palsy | 2016 | Duplicate Article | No |
| 739 | Hyperactivity and disturbed motor coordination in adolescent mice after neonatal expsoure to hyperoxia | 2012 | Title and abstract clearly do not meet inclusion criteria | No |
| 740 | The role of modern imaging modalities on deep brain stimulation targeting for mental illness | 2008 | Title and abstract clearly do not meet inclusion criteria | No |
| 741 | Which factors predict outcomes of neonates with hypoxic-ischemic encephalopathy following therapeutic hypothermia? | 2021 | Non-CP | No |
| 742 | Imaging for diagnosis and treatment of cerebral palsy | 2008 | Title and abstract clearly do not meet inclusion criteria | No |
| 743 | MRI-based radiologic scoring system for extent of brain injury in children with hemiplegia | 2014 | Duplicate Article | No |
| 744 | Sex differences in outcome and associations with neonatal brain morphology in extremely preterm children | 2014 | Title and abstract clearly do not meet inclusion criteria | No |
| 745 | Diffusion tensor imaging demonstrates focal lesions of the corticospinal tract in hemiparetic patients with cerebral palsy | 2007 | Duplicate Article | No |
| 746 | Diffusion tensor tractography can predict hemiparesis in infants with high risk factors | 2009 | Duplicate Article | No |
| 747 | Response to Correspondence on “Stress in Parents of Children With Genetically Determined Leukoencephalopathies: A Pilot Study” | 2021 | Title and abstract clearly do not meet inclusion criteria | No |
| 748 | Reorganization after pre- and perinatal brain lesions | 2010 | Title and abstract clearly do not meet inclusion criteria | No |
| 749 | Early motor outcomes in infants with critical congenital heart disease are related to neonatal brain development and brain injury | 2022 | Title and abstract clearly do not meet inclusion criteria | No |
| 750 | Functional assessment and DTI-MRI correlates of congenital hemiplegia | 2009 | Title and abstract clearly do not meet inclusion criteria | No |
| 751 | Diffusion Tensor Imaging to Predict Neurodevelopmental Impairment in Infants After Hypoxic-Ischemic Injury | 2022 | No correlation analysis | No |
| 752 | Association of Diffusion Tensor Imaging Measures at Term with Absent Fidgety Movements in Infants with Hypoxic-ischemic Encephalopathy | 2022 | No correlation analysis | No |
| 753 | Sex influences on the neurocognitive outcome of preterm children | 2023 | Title and abstract clearly do not meet inclusion criteria | No |
| 754 | Protocol for the Birth Asphyxia in African Newborns (Baby BRAiN) Study: A Neonatal Encephalopathy Feasibility Cohort Study | 2022 | Title and abstract clearly do not meet inclusion criteria | No |
| 755 | Longitudinal evaluation of DTI for children with cerebral palsy | 2012 | Title and abstract clearly do not meet inclusion criteria | No |
| 756 | Quantitative diffusion tensor imaging in cerebral palsy due to periventricular white matter injury | 2005 | Duplicate Article | No |
| 757 | Concurrent erythropoietin and hypothermia treatment improve outcomes in a term nonhuman primate model of perinatal asphyxia | 2013 | Duplicate Article | No |
| 758 | Concurrent erythropoietin and hypothermia treatment improve outcomes in a term nonhuman primate model of perinatal asphyxia | 2013 | Duplicate Article | No |
| 759 | Neuroimaging of structural and functional connectivity in preterm infants with intraventricular hemorrhage | 2022 | Non-CP | No |
| 760 | Correlation of quantitative sensorimotor tractography with clinical grade of cerebral palsy | 2010 | Duplicate Article | No |
| 761 | Treatment-Induced Plasticity in Cerebral Palsy: A Diffusion Tensor Imaging Study | 2008 | Duplicate Article | No |
| 762 | Review: Clinical application of diffusion tensor imaging | 2008 | review | No |
| 763 | Neurophysiological assessments of brain and spinal cord associated with lower limb functions in children with cerebral palsy: A protocol for systematic review and meta-analysis | 2021 | review | No |
| 764 | Combining advanced MRI and EEG techniques better explains long-term motor outcome after very preterm birth | 2022 | Title and abstract clearly do not meet inclusion criteria | No |
| 765 | Does diffusion tensor imaging-based tractography at 3 months of age contribute to the prediction of motor outcome after perinatal arterial ischemic stroke? | 2011 | Non-CP | No |
| 766 | Relation between brain lesions on MRI and gait pathology in children with cerebral palsy | 2013 |  | Yes |
| 767 | Outcome Assessment and Function in Cerebral Palsy | 2020 | Title and abstract clearly do not meet inclusion criteria | No |
| 768 | Neonatal microstructural development of the left superior temporal gyrus and cognitive development in very low birth weight preterm children at 18-20 months: A diffusion imaging study | 2013 | Title and abstract clearly do not meet inclusion criteria | No |
| 769 | Prediction of language development using forward feature selection and linear regression model of near-term regional brain white matter microstructure in children born preterm | 2016 | No correlation analysis | No |
| 770 | Developmental Abnormalities of Temporal Lobe in Children | 2008 | Title and abstract clearly do not meet inclusion criteria | No |
| 771 | Improved Myelination following Camp Leg Power, a Selective Motor Control Intervention for Children with Spastic Bilateral Cerebral Palsy: A Diffusion Tensor MRI Study | 2023 | Duplicate Article | No |
| 772 | Early prediction of unilateral cerebral palsy in infants at risk: MRI versus the hand assessment for infants | 2020 | Duplicate Article | No |
| 773 | Endoscope-assisted hemispherotomy: Translation of technique from cadaveric anatomical feasibility study to clinical implementation | 2019 | Title and abstract clearly do not meet inclusion criteria | No |
| 774 | Diffusion tensor MR imaging in the assessment of cognitive functions in children with periventricular leukomalacia | 2012 | No correlation analysis | No |
| 775 | Effects of three kinds of head acupuncture therapies on regulation of brain microenvironment and rehabilitation of nerve function in rats with cerebral palsy | 2021 | Duplicate Article | No |
| 776 | Quantification and Monitoring of the Effect of Botulinum Toxin A on Paretic Calf Muscles of Children With Cerebral Palsy With MRI: A Preliminary Study | 2021 | Duplicate Article | No |
| 777 | Understanding the relationship between brain and upper limb function in children with unilateral motor impairments: A multimodal approach | 2018 | Duplicate Article | No |
| 778 | Does damage to somatosensory circuits underlie motor impairment in cerebral palsy? | 2009 | Title and abstract clearly do not meet inclusion criteria | No |
| 779 | Retinal optical coherence tomography for children with cerebral palsy | 2020 | Title and abstract clearly do not meet inclusion criteria | No |
| 780 | Magnetic resonance imaging of bilirubin encephalopathy: Current limitations and future promise | 2014 | Title and abstract clearly do not meet inclusion criteria | No |
| 781 | Efficacy and mechanism of scalp acupuncture for spastic cerebral palsy | 2023 | Title and abstract clearly do not meet inclusion criteria | No |
| 782 | Developmental outcomes of very preterm and extremely preterm infants at the 12 months corrected age | 2023 | Title and abstract clearly do not meet inclusion criteria | No |
| 783 | DTI for stroke imaging | 2010 | Title and abstract clearly do not meet inclusion criteria | No |
| 784 | Morphological assessments of neonatal hypoxia-ischemia: White matter and blood-brain barrier injury | 2012 | Title and abstract clearly do not meet inclusion criteria | No |
| 785 | Neural plasticity after pre-linguistic injury to the arcuate and superior longitudinal fasciculi | 2013 | Title and abstract clearly do not meet inclusion criteria | No |
| 786 | Neuroimaging in developmental coordination disorder | 2021 | Title and abstract clearly do not meet inclusion criteria | No |
| 787 | Anatomical characterization of athetotic and spastic cerebral palsy using an atlas-based analysis | 2013 | Title and abstract clearly do not meet inclusion criteria | No |
| 788 | Diffusion-weighted imaging and diffusion-tensor imaging study of children with a clinical diagnosis of athetotic cerebral palsy | 2010 | Title and abstract clearly do not meet inclusion criteria | No |
| 789 | Athetotic and spastic cerebral palsy: Anatomic characterization based on diffusion-tensor imaging | 2011 | Title and abstract clearly do not meet inclusion criteria | No |
| 790 | Quantitative diffusion tensor tractography of the motor and sensory tract in children with cerebral palsy | 2010 | Title and abstract clearly do not meet inclusion criteria | No |
| 791 | Changes of White Matter Diffusion Anisotropy in Response to a 6-Week iPad Application-Based Occupational Therapy Intervention in Children with Surgically Treated Hydrocephalus: A Pilot Study | 2016 | Title and abstract clearly do not meet inclusion criteria | No |
| 792 | Use of Thalamus L-Sign to Differentiate Periventricular Leukomalacia From Neurometabolic Disorders | 2023 | Title and abstract clearly do not meet inclusion criteria | No |
| 793 | Pathophysiology of periventricular leukomalacia: What we learned from animal models | 2017 | review | No |
| 794 | Cerebral white matter injuries following a hypoxic/ischemic insult during the perinatal period: Pathophysiology, prognostic factors, and future strategy of treatment approach. a minireview | 2015 | review | No |
| 795 | The safety and efficacy of umbilical cord blood mononuclear cells in individuals with spastic cerebral palsy: a randomized double-blind sham-controlled clinical trial | 2022 | No correlation analysis | No |
| 796 | Multivariate analysis and machine learning in cerebral palsy research | 2017 | review | No |
| 797 | DTI Tract-Based Quantitative Susceptibility Mapping: An Initial Feasibility Study to Investigate the Potential Role of Myelination in Brain Connectivity Change in Cerebral Palsy Patients During Autologous Cord Blood Cell Therapy Using a Rotationally-Invariant Quantitative Measure | 2021 | Title and abstract clearly do not meet inclusion criteria | No |
| 798 | Changes of Structural Brain Network Following Repetitive Transcranial Magnetic Stimulation in Children With Bilateral Spastic Cerebral Palsy: A Diffusion Tensor Imaging Study | 2020 | No correlation analysis | No |
| 799 | Illness severity in the first 24 hours of life is related to abnormal motor pathway development in preterm newborns | 2012 | Non-CP | No |
| 800 | Score for neonatal acute physiology-II and neonatal pain predict Corticospinal tract development in premature newborns | 2013 | Non-CP | No |
| From Web of science | | | | |
| 801 | 21st Congress of the European Society for Stereotactic and Functional Neurosurgery, Maastrict, Netherlands, September 17-20, 2014 Abstracts | 2014 | Title and abstract clearly do not meet inclusion criteria | No |
| 802 | British Society for Stereotactic and Functional Neurosurgery, Cambridge 23 and 24 May 2019 Abstracts | 2019 | Title and abstract clearly do not meet inclusion criteria | No |
| 803 | Neuroimaging in cerebral palsy | 2004 | Title and abstract clearly do not meet inclusion criteria | No |
| 804 | Early Imaging and Adverse Neurodevelopmental Outcome in Asphyxiated Newborns Treated With Hypothermia | 2017 | Duplicate Article | No |
| 805 | A connectome-based approach to assess motor outcome after neonatal arterial ischemic stroke | 2021 | Duplicate Article | No |
| 806 | Diffusion tensor imaging detects ventilation-induced brain injury in preterm lambs | 2017 | Title and abstract clearly do not meet inclusion criteria | No |
| 807 | MR Imaging and Outcome of Term Neonates with Perinatal Asphyxia: Value of Diffusion-weighted MR Imaging and <SUP>1</SUP>H MR Spectroscopy | 2011 | Title and abstract clearly do not meet inclusion criteria | No |
| 808 | Effects of Piano Training in Unilateral Cerebral Palsy Using Probabilistic and Deterministic Tractography: A Case Report | 2021 | Duplicate Article | No |
| 809 | Clinical and imaging outcomes after intrathecal injection of umbilical cord tissue mesenchymal stem cells in cerebral palsy: a randomized double-blind sham-controlled clinical trial | 2021 | Duplicate Article | No |
| 810 | DIFFUSION WEIGHTED MAGNETIC RESONANCE IMAGING IN CHILD CEREBRAL PALSY WITH SYMPTOMATIC EPILEPSY | 2017 | Duplicate Article | No |
| 811 | Diffusion Weighted Magnetic Resonance Imaging in Child Cerebral Palsy with Symptomatic Epilepsy | 2017 | Title and abstract clearly do not meet inclusion criteria | No |
| 812 | Specific indicators of diffusion weighted magnetic resonance imaging in child cerebral palsy with symptomatic epilepsy | 2019 | Title and abstract clearly do not meet inclusion criteria | No |
| 813 | Association between preterm brain injury and exposure to chorioamnionitis during fetal life | 2016 | Title and abstract clearly do not meet inclusion criteria | No |
| 814 | Advanced MR imaging of the placenta: Exploring the in utero placenta-brain connection | 2015 | Title and abstract clearly do not meet inclusion criteria | No |
| 815 | Clinical and Radiological Profiles of COVID-19 Patients with Neurological Symptomatology: A Comparative Study | 2021 | Title and abstract clearly do not meet inclusion criteria | No |
| 816 | Impact of early brain lesions on the optic radiations in children with cerebral palsy | 2022 | Duplicate Article | No |
| 817 | Brain lesions in preterm infants: initial diagnosis and follow-up | 2010 | Duplicate Article | No |
| 818 | The effects of hemorrhagic parenchymal infarction on the establishment of sensori-motor structural and functional connectivity in early infancy | 2014 | Duplicate Article | No |
| 819 | Whole-Brain DTI Assessment of White Matter Damage in Children with Bilateral Cerebral Palsy: Evidence of Involvement beyond the Primary Target of the Anoxic Insult | 2016 | Duplicate Article | No |
| 820 | Diffusion tensor brain imaging findings at term-equivalent age may predict neurologic abnormalities in low birth weight preterm infants | 2003 | Duplicate Article | No |
| 821 | A RARE CASE OF PERCHERON ARTERY INFARCT | 2015 | Title and abstract clearly do not meet inclusion criteria | No |
| 822 | Impact of anti-gravity locomotion (AlterG) training on structure and function of corticospinal tract and gait in children with cerebral palsy | 2019 | Duplicate Article | No |
| 823 | Assessment of neuroplasticity of corticospinal tract induced by antigravity treadmill (AlterG) in cerebral palsy children | 2018 | Duplicate Article | No |
| 824 | Contribution of altered corticospinal microstructure to gait impairment in children with cerebral palsy | 2021 | Duplicate Article | No |
| 825 | The impact of an anti-gravity treadmill (AlterG) training on walking capacity and corticospinal tract structure in children with cerebral palsy | 2017 | Duplicate Article | No |
| 826 | The impact of AlterG training on balance and structure of vestibulospinal tract in cerebral palsy children | 2018 | Duplicate Article | No |
| 827 | Early predictors of neurodevelopment after perinatal arterial ischemic stroke: a systematic review and meta-analysis | 2023 | review | No |
| 828 | CST recovery in pediatric hemiplegic patients: Diffusion tensor tractography study | 2013 | Duplicate Article | No |
| 829 | Susceptibility-Weighted Imaging Identifies Iron-Oxide-Labeled Human Neural Stem Cells: Automated Computational Detection | 2016 | Duplicate Article | No |
| 830 | Whole-Brain Structural Connectivity in Dyskinetic Cerebral Palsy and Its Association With Motor and Cognitive Function | 2017 | Duplicate Article | No |
| 831 | Neuroimaging in perinatal hypoxic-ischemic injury | 1997 | Title and abstract clearly do not meet inclusion criteria | No |
| 832 | Segmental Alterations of the Corpus Callosum in Progressive Supranuclear Palsy: A Multiparametric Magnetic Resonance Imaging Study | 2021 | Title and abstract clearly do not meet inclusion criteria | No |
| 833 | Prophylactic maternal N-acetylcysteine in rats prevents maternal inflammation-induced offspring cerebral injury shown on magnetic resonance imaging | 2013 | Duplicate Article | No |
| 834 | The influence of body temperature on tissue stiffness, blood perfusion, and water diffusion in the mouse brain | 2019 | Title and abstract clearly do not meet inclusion criteria | No |
| 835 | Biomechanical properties of the hypoxic and dying brain quantified by magnetic resonance elastography | 2020 | Title and abstract clearly do not meet inclusion criteria | No |
| 836 | Developmental Dynamic Dysphasia: Are Bilateral Brain Abnormalities a Signature of Inefficient Neural Plasticity? | 2020 | Title and abstract clearly do not meet inclusion criteria | No |
| 837 | Neuroimaging Perspectives of Perinatal Arterial Ischemic Stroke | 2020 | Duplicate Article | No |
| 838 | Image-based musculoskeletal modeling: Applications, advances, and future opportunities | 2007 | Title and abstract clearly do not meet inclusion criteria | No |
| 839 | Motor Skill Training May Restore Impaired Corticospinal Tract Fibers in Children With Cerebral Palsy | 2020 | Duplicate Article | No |
| 840 | Capturing neuroplastic changes after bimanual intensive rehabilitation in children with unilateral spastic cerebral palsy: A combined DTI, TMS and fMRI pilot study | 2015 | No correlation analysis | No |
| 841 | Corticospinal dysgenesis and upper-limb deficits in congenital hemiplegia: A diffusion tensor imaging study | 2007 | No correlation analysis | No |
| 842 | A new framework for analysis of three-dimensional shape and architecture of human skeletal muscles from in vivo imaging data | 2022 | Duplicate Article | No |
| 843 | Cerebral MRI of Preterm Neonates at Term Equivalent Age at 3 Tesla: Hints at White Matter Damage on T2-and Diffusion Weighted Images | 2010 | Title and abstract clearly do not meet inclusion criteria | No |
| 844 | Mitii™ ABI: study protocol of a randomised controlled trial of a web-based multi-modal training program for children and adolescents with an Acquired Brain Injury (ABI) | 2015 | Duplicate Article | No |
| 845 | Microstructure of the Dorsal Anterior Cingulum Bundle in Very Preterm Neonates Predicts the Preterm Behavioral Phenotype at 5 Years of Age | 2021 | Title and abstract clearly do not meet inclusion criteria | No |
| 846 | Diffusion Tensor Imaging Abnormalities in the Cerebral White Matter Correlate with Sex-Dependent Neurobehavioral Deficits in Adult Mice with Neonatal Ischemia | 2016 | No correlation analysis | No |
| 847 | Parechovirus Encephalitis and Neurodevelopmental Outcomes | 2016 | Title and abstract clearly do not meet inclusion criteria | No |
| 848 | Imaging Approaches to Parkinson Disease | 2010 | Title and abstract clearly do not meet inclusion criteria | No |
| 849 | Neuroimaging and Occupational Therapy: Bridging the Gap to Advance Rehabilitation in Developmental Coordination Disorder | 2017 | Title and abstract clearly do not meet inclusion criteria | No |
| 850 | Effect of a NICU to Home Physical Therapy Intervention on White Matter Trajectories, Motor Skills, and Problem-Solving Skills of Infants Born Very Preterm: A Case Series | 2022 | Title and abstract clearly do not meet inclusion criteria | No |
| 851 | Development of the corpus callosum and cognition after neonatal encephalopathy | 2023 | Duplicate Article | No |
| 852 | Brain-behavior relationships in young traumatic brain injury patients: Fractional anisotropy measures are highly correlated with dynamic visuomotor tracking performance | 2010 | Title and abstract clearly do not meet inclusion criteria | No |
| 853 | Etiology of impaired selective motor control: emerging evidence and its implications for research and treatment in cerebral palsy | 2014 | Duplicate Article | No |
| 854 | Diffusion Tensor Magnetic Resonance Imaging Tractography in Progressive Supranuclear Palsy | 2011 | Duplicate Article | No |
| 855 | Atypical "nine" syndrome in bilateral pontine infarction A case report | 2019 | Title and abstract clearly do not meet inclusion criteria | No |
| 856 | Structural and functional connectivity of motor circuits after perinatal stroke: A machine learning study | 2020 | Title and abstract clearly do not meet inclusion criteria | No |
| 857 | Electric field simulations of transcranial direct current stimulation in children with perinatal stroke | 2023 | Duplicate Article | No |
| 858 | White Matter Tract Changes Associated with Clinical Improvement in an Open-Label Trial Assessing Autologous Umbilical Cord Blood for Treatment of Young Children with Autism | 2019 | Title and abstract clearly do not meet inclusion criteria | No |
| 859 | Regional vulnerability of longitudinal cortical association connectivity Associated with structural network topology alterations in preterm children with cerebral palsy | 2015 | Duplicate Article | No |
| 860 | Clinical severity in CADASIL related to ultrastructural damage in white matter - In vivo study with diffusion tensor MRI | 1999 | No correlation analysis | No |
| 861 | Cortical ischaemic patterns in term partial-prolonged hypoxic-ischaemic injury-the inter-arterial watershed demonstrated through atrophy, ulegyria and signal change on delayed MRI scans in children with cerebral palsy | 2020 | Title and abstract clearly do not meet inclusion criteria | No |
| 862 | Early micro- and macrostructure of sensorimotor tracts and development of cerebral palsy in high risk infants | 2021 | Title and abstract clearly do not meet inclusion criteria | No |
| 863 | Degenerative changes of the corticospinal tract in pediatric patients showing deteriorated motor function: A diffusion tensor tractography study | 2015 | Duplicate Article | No |
| 864 | Diffusion tensor imaging demonstrated radiologic differences between diplegic and quadriplegic cerebral palsy | 2012 | Duplicate Article | No |
| 865 | Double decussated ipsilateral corticospinal tract in schizencephaly | 2009 | Title and abstract clearly do not meet inclusion criteria | No |
| 866 | Comparative assessment of therapeutic response to physiotherapy with or without botulinum toxin injection using diffusion tensor tractography and clinical scores in term diplegic cerebral palsy children | 2013 | Duplicate Article | No |
| 867 | Postnatal infection is associated with widespread abnormalities of brain development in premature newborns | 2012 | Title and abstract clearly do not meet inclusion criteria | No |
| 868 | Chorioamnionitis in the Pathogenesis of Brain Injury in Preterm Infants | 2014 | Duplicate Article | No |
| 869 | Study of Effect of Sympathetic Nerve on Children?s Brain Diseases Based on Analysis of Magnetic Resonance Imaging Kurtosis | 2020 | Title and abstract clearly do not meet inclusion criteria | No |
| 870 | Intravenous thrombolytic therapy in patients with stroke mimics: baseline characteristics and safety profile | 2011 | Duplicate Article | No |
| 871 | Case report and literature review: fatal cerebral fat embolism following facial autologous fat graft | 2023 | review | No |
| 872 | <i>In utero</i> methadone exposure permanently alters anatomical and functional connectivity: A preclinical evaluation | 2023 | Title and abstract clearly do not meet inclusion criteria | No |
| 873 | Objective and Clinically Feasible Analysis of Diffusion MRI Data can Help Predict Dystonia After Neonatal Brain Injury | 2021 | Title and abstract clearly do not meet inclusion criteria | No |
| 874 | Diffusion Tensor Imaging-Demonstrated Differences between Hemiplegic and Diplegic Cerebral Palsy with Symmetric Periventricular Leukomalacia | 2013 | Duplicate Article | No |
| 875 | Neurodevelopmental Outcomes of Neonatal Rotavirus-Associated Leukoencephalopathy | 2022 | Duplicate Article | No |
| 876 | Traumatic bilateral common carotid artery dissection due to strangulation - A case report | 2006 | Title and abstract clearly do not meet inclusion criteria | No |
| 877 | Three-dimensional skeletal muscle architecture in the lower legs of living human infants | 2023 | Title and abstract clearly do not meet inclusion criteria | No |
| 878 | Cerebral venous thrombosis associated with tentorial subdural hematoma during oxymetholone therapy | 2001 | Title and abstract clearly do not meet inclusion criteria | No |
| 879 | Consequences of Intraventricular Hemorrhage in a Rabbit Pup Model | 2009 | Duplicate Article | No |
| 880 | Hypoglycemic encephalopathy mimicking acute ischemic stroke in clinical presentation and magnetic resonance imaging: a case report | 2019 | Title and abstract clearly do not meet inclusion criteria | No |
| 881 | Gray and White Matter Correlates of Dysphagia in Progressive Supranuclear Palsy | 2021 | Duplicate Article | No |
| 882 | Correlating early motor skills to white matter abnormalities in preterm infants using diffusion tensor imaging | 2016 | No correlation analysis | No |
| 883 | Full Activation Profiles and Integrity of Corticospinal Pathways in Adults With Bilateral Spastic Cerebral Palsy | 2019 | Duplicate Article | No |
| 884 | Thalamo-cortical connectivity in children born preterm mapped using probabilistic magnetic resonance tractography | 2007 | Title and abstract clearly do not meet inclusion criteria | No |
| 885 | Axial and radial diffusivity in preterm infants who have diffuse white matter changes on magnetic resonance imaging at term-equivalent age | 2006 | Title and abstract clearly do not meet inclusion criteria | No |
| 886 | Autologous cellular therapy for cerebral palsy: a randomized, crossover trial | 2022 | Title and abstract clearly do not meet inclusion criteria | No |
| 887 | Developmental neuroplasticity of the white matter connectome in children with perinatal stroke | 2020 | Title and abstract clearly do not meet inclusion criteria | No |
| 888 | Imaging Developmental and Interventional Plasticity Following Perinatal Stroke | 2021 | Duplicate Article | No |
| 889 | Imaging diagnosis and legal implications of brain injury in survivors following single intrauterine fetal demise from monochorionic twins - a review of the literature | 2021 | review | No |
| 890 | Thalamopeduncular Tumors in Pediatric Age: Advanced Preoperative Imaging to Define Safe Surgical Planning: A Multicentric Experience | 2023 | Duplicate Article | No |
| 891 | Muscle architecture in children with cerebral palsy and ankle contractures: an investigation using diffusion tensor imaging | 2019 | Duplicate Article | No |
| 892 | Central tegmental tract hyperintensity: follow-up outcomes from a single-center study | 2023 | Title and abstract clearly do not meet inclusion criteria | No |
| 893 | The role of neuroimaging in the diagnosis of the atypical parkinsonian syndromes in clinical practice | 2015 | Title and abstract clearly do not meet inclusion criteria | No |
| 894 | CSF neopterin and quinolinic acid are biomarkers of neuroinflammation and neurotoxicity in FIRES and other infection-triggered encephalopathy syndromes | 2023 | Title and abstract clearly do not meet inclusion criteria | No |
| 895 | Functional and Structural Brain Connectivity in Children With Bilateral Cerebral Palsy Compared to Age-Related Controls and in Response to Intensive Rapid-Reciprocal Leg Training | 2022 | Title and abstract clearly do not meet inclusion criteria | No |
| 896 | Tractography of white-matter tracts in very preterm infants: a 2-year follow-up study | 2013 | Duplicate Article | No |
| 897 | Arterial spin-labelling perfusion MRI and outcome in neonates with hypoxic-ischemic encephalopathy | 2015 | Non-CP | No |
| 898 | Myth: Cerebral palsy cannot be predicted by neonatal brain imaging | 2011 | Non-CP | No |
| 899 | Diffusion tensor imaging in a patient with cerebral palsy and hypersomnia Introduction | 2014 | No correlation analysis | No |
| 900 | Very preterm children at risk for developmental coordination disorder have brain alterations in motor areas | 2019 | Duplicate Article | No |
| 901 | Is Faster Better? Relationships of Cesarean Acuity with Risk of Low Apgar Scores and Fetal Acidemia | 2023 | Title and abstract clearly do not meet inclusion criteria | No |
| 902 | Long Term Motor Function after Neonatal Stroke: Lesion Localization above All | 2015 | Title and abstract clearly do not meet inclusion criteria | No |
| 903 | Harnessing Neuroimaging Capability in Pediatric Stroke: Proceedings of the Stroke Imaging Laboratory for Children Workshop | 2017 | Title and abstract clearly do not meet inclusion criteria | No |
| 904 | Early Detection of Hypothermic Neuroprotection Using T2-Weighted Magnetic Resonance Imaging in a Mouse Model of Hypoxic Ischemic Encephalopathy | 2018 | Title and abstract clearly do not meet inclusion criteria | No |
| 905 | Concurrent Decrease of Brain White Matter Tracts' Thicknesses and Fractional Anisotropy After Antenatal Hypoxia-Ischemia Detected With Tract-Based Spatial Statistics Analysis | 2017 | Duplicate Article | No |
| 906 | Near-Term Fetal Hypoxia-Ischemia in Rabbits MRI Can Predict Muscle Tone Abnormalities and Deep Brain Injury | 2012 | Title and abstract clearly do not meet inclusion criteria | No |
| 907 | Fetal brain magnetic resonance imaging response acutely to hypoxia-ischemia predicts postnatal outcome | 2007 | Title and abstract clearly do not meet inclusion criteria | No |
| 908 | White matter injury correlates with hypertonia in an animal model of cerebral palsy | 2007 | Title and abstract clearly do not meet inclusion criteria | No |
| 909 | Unmyelinated Axon Loss with Postnatal Hypertonia after Fetal Hypoxia | 2014 | Duplicate Article | No |
| 910 | Motor Deficits Are Triggered by Reperfusion-Reoxygenation Injury as Diagnosed by MRI and by a Mechanism Involving Oxidants | 2012 | Title and abstract clearly do not meet inclusion criteria | No |
| 911 | Spinal cord injury in hypertonic newborns after antenatal hypoxia-ischemia in a rabbit model of cerebral palsy | 2017 | Duplicate Article | No |
| 912 | Developmental changes in diffusion anisotropy coincide with immature oligodendrocyte progression and maturation of compound action potential | 2005 | Duplicate Article | No |
| 913 | Occult Spastic Diplegic Cerebral Palsy Recognition Using Efficient Machine Learning for Big Data and Structural Connectivity Abnormalities Analysis | 2018 | Title and abstract clearly do not meet inclusion criteria | No |
| 914 | Brain Development in Infants Born Preterm: Looking Beyond Injury | 2013 | Duplicate Article | No |
| 915 | Neonatal Neurobehavior and Diffusion MRI Changes in Brain Reorganization Due to Intrauterine Growth Restriction in a Rabbit Model | 2012 | Title and abstract clearly do not meet inclusion criteria | No |
| 916 | Can diffusion tensor imaging predict cerebral palsy in term neonates with hypoxic ischemic encephalopathy? | 2019 | Duplicate Article | No |
| 917 | Diffuse reduction of white matter connectivity in cerebral palsy with specific vulnerability of long range fiber tracts | 2013 | Duplicate Article | No |
| 918 | Brain structural connectivity increases concurrent with functional improvement: Evidence from diffusion tensor MRI in children with cerebral palsy during therapy | 2015 | Duplicate Article | No |
| 919 | Abnormal corpus callosum in neonates after hypoxic-ischemic injury | 2012 | Title and abstract clearly do not meet inclusion criteria | No |
| 920 | Diffusion tractography and neuromotor outcome in very preterm children with white matter abnormalities | 2014 | Title and abstract clearly do not meet inclusion criteria | No |
| 921 | Potential of diffusion tensor MRI in the assessment of periventricular leukomalacia | 2006 | No correlation analysis | No |
| 922 | Quantitative analysis of brain pathology based on MRI and brain atlases-Applications for cerebral palsy | 2011 | No correlation analysis | No |
| 923 | Atlas-based analysis of neurodevelopment from infancy to adulthood using diffusion tensor imaging and applications for automated abnormality detection | 2010 | No correlation analysis | No |
| 924 | Systemic Inflammation Disrupts the Developmental Program of White Matter | 2011 | Title and abstract clearly do not meet inclusion criteria | No |
| 925 | Coaction of individual and environmental factors: a review of intensive therapy paradigms for children with unilateral spastic cerebral palsy | 2017 | review | No |
| 926 | Uncrossed corticospinal tract in health and genetic disorders: Review, case report, and clinical implications | 2021 | Duplicate Article | No |
| 927 | Plasticity following early-life brain injury: Insights from quantitative MRI | 2015 | Title and abstract clearly do not meet inclusion criteria | No |
| 928 | Validity of semi-quantitative scale for brain MRI in unilateral cerebral palsy due to periventricular white matter lesions: Relationship with hand sensorimotor function and structural connectivity | 2015 | Duplicate Article | No |
| 929 | Corticopontocerebellar Connectivity Disruption in Congenital Hemiplegia | 2015 | Duplicate Article | No |
| 930 | Lower-extremity selective voluntary motor control in patients with spastic cerebral palsy: increased distal motor impairment | 2010 | No correlation analysis | No |
| 931 | An Individualized Approach to Neuroplasticity After Early Unilateral Brain Damage | 2019 | Duplicate Article | No |
| 932 | Effects of gestational age at birth on perinatal structural brain development in healthy term-born babies | 2022 | No correlation analysis | No |
| 933 | Magnetic Resonance Imaging Correlates of White Matter Gliosis and Injury in Preterm Fetal Sheep Exposed to Progressive Systemic Inflammation | 2020 | Title and abstract clearly do not meet inclusion criteria | No |
| 934 | White matter changes associated with cognitive visual dysfunctions in children with cerebral palsy: A diffusion tensor imaging study | 2018 | Duplicate Article | No |
| 935 | Bilateral Thalamic Ischemic Stroke Secondary to Occlusion of the Artery of Percheron | 2018 | Duplicate Article | No |
| 936 | White Matter Involvement in Idiopathic Parkinson Disease: A Diffusion Tensor Imaging Study | 2009 | Title and abstract clearly do not meet inclusion criteria | No |
| 937 | Diffusion tensor imaging of pyramidal tract reorganization after pediatric stroke | 2014 | No correlation analysis | No |
| 938 | Prediction of childhood brain outcomes in infants born preterm using neonatal MRI and concurrent clinical biomarkers (PREBO-6): study protocol for a prospective cohort study | 2020 | Title and abstract clearly do not meet inclusion criteria | No |
| 939 | Correlation Between White Matter Injury Identified by Neonatal Diffusion Tensor Imaging and Neurodevelopmental Outcomes Following Term Neonatal Asphyxia and Therapeutic Hypothermia: An Exploratory Pilot Study | 2019 | Non-CP | No |
| 940 | MAGNESIUM SULFATE (MG) PREVENTS MATERNAL INFLAMMATION INDUCED OFFSPRING CEREBRAL INJURY EVIDENT ON MRI BUT NOT VIA IL-1β | 2017 | Title and abstract clearly do not meet inclusion criteria | No |
| 941 | Diffusion tensor MR imaging tractography of the pyramidal tracts correlates with clinical motor function in children with congenital hemiparesis | 2007 | No correlation analysis | No |
| 942 | Nuclear imaging in Parkinson's disease: The past, the present, and the future | 2022 | Title and abstract clearly do not meet inclusion criteria | No |
| 943 | Regional microstructural damage and patterns of eye movement impairment: a DTI and video-oculography study in neurodegenerative parkinsonian syndromes | 2017 | Duplicate Article | No |
| 944 | Association of transcallosal motor fibres with function of both hands after unilateral neonatal arterial ischemic stroke | 2017 | Title and abstract clearly do not meet inclusion criteria | No |
| 945 | Motor Abilities in Adolescents Born Preterm Are Associated With Microstructure of the Corpus Callosum | 2019 | Duplicate Article | No |
| 946 | The Impact of Early Neuroimaging and Developmental Assessment in a Preterm Infant Diagnosed with Cerebral Palsy | 2019 | Title and abstract clearly do not meet inclusion criteria | No |
| 947 | Effect of sensory and motor connectivity on hand function in pediatric hemiplegia | 2017 | Duplicate Article | No |
| 948 | Neural Correlates of Impaired Grasp Function in Children with Unilateral Spastic Cerebral Palsy | 2023 | Duplicate Article | No |
| 949 | Case Report: Brain Metastasis Confined to the Infarcted Area Following Stroke | 2021 | Title and abstract clearly do not meet inclusion criteria | No |
| 950 | Changes of Neural Pathways after Vojta Approach in a Child with Developmental Delay | 2021 | Title and abstract clearly do not meet inclusion criteria | No |
| 951 | Language Comprehension in Young People with Severe Cerebral Palsy in Relation to Language Tracts: A Diffusion Tensor Imaging Study | 2013 | No correlation analysis | No |
| 952 | Diffusion-weighted imaging changes in cerebral watershed distribution following neonatal encephalopathy are not invariably associated with an adverse outcome | 2013 | Non-CP | No |
| 953 | Creutzfeldt-Jakob Disease with Paralysis of the Unilateral Vocal Cord and Soft Palate | 2011 | Duplicate Article | No |
| 954 | Cerebellar peduncle injury predicts motor impairments in preterm infants: A quantitative tractography study at term-equivalent age | 2018 | Duplicate Article | No |
| 955 | Superior alternating hemiplegia (Weber?s syndrome)- Case report | 2022 | Title and abstract clearly do not meet inclusion criteria | No |
| 956 | Assessment of the Contralesional Corticospinal Tract in Early-Onset Pediatric Hemiplegia: Preliminary Findings | 2014 | Title and abstract clearly do not meet inclusion criteria | No |
| 957 | Pontine and cerebellar injury in neonatal hypoxic-ischemic encephalopathy: MRI features and clinical outcomes | 2020 | Duplicate Article | No |
| 958 | Long-term preservation of pharyngeal swallowing function in MM2-cortical-type sporadic Creutzfeldt-Jakob disease | 2021 | Duplicate Article | No |
| 959 | Changes in the Thalamus in Atypical Parkinsonism Detected Using Shape Analysis and Diffusion Tensor Imaging | 2014 | Duplicate Article | No |
| 960 | Learning an Infant Body Model from RGB-D Data for Accurate Full Body Motion Analysis | 2018 | Title and abstract clearly do not meet inclusion criteria | No |
| 961 | Pure Motor Monoparesis Due to Ischemic Stroke | 2011 | Title and abstract clearly do not meet inclusion criteria | No |
| 962 | Segmental Diffusion Properties of the Corticospinal Tract and Motor Outcome in Hemiparetic Children With Perinatal Stroke | 2017 | Duplicate Article | No |
| 963 | White matter alterations and their associations with motor function in young adults born preterm with very low birth weight | 2018 | Duplicate Article | No |
| 964 | Diffusion MRI in corticofugal fibers correlates with hand function in unilateral cerebral palsy | 2011 | Duplicate Article | No |
| 965 | Neuroimaging in cerebral palsy: Patterns of brain dysgenesis and injury | 2005 | Duplicate Article | No |
| 966 | Cerebral Palsy | 2007 | review | No |
| 967 | Neuroimaging in spasticity and movement disorders | 2003 | Duplicate Article | No |
| 968 | PATHOGENESIS, NEUROIMAGING AND MANAGEMENT IN CHILDREN WITH CEREBRAL PALSY BORN PRETERM | 2010 | review | No |
| 969 | Diffusion tensor imaging of periventricular leukomalacia shows affected sensory cortex white matter pathways | 2002 | No correlation analysis | No |
| 970 | Neuroimaging: Applications in disorders of early brain development | 2000 | Duplicate Article | No |
| 971 | Sensory and motor deficits in children with cerebral palsy born preterm correlate with diffusion tensor imaging abnormalities in thalamocortical pathways | 2009 | Title and abstract clearly do not meet inclusion criteria | No |
| 972 | Sensory and motor deficits in children with cerebral palsy born preterm correlate with diffusion tensor imaging abnormalities in thalamocortical pathways (vol 51, pg 697, 2009) | 2009 | Title and abstract clearly do not meet inclusion criteria | No |
| 973 | Potential of Advanced MR Imaging Techniques in the Differential Diagnosis of Parkinsonism | 2009 | Title and abstract clearly do not meet inclusion criteria | No |
| 974 | White and gray matter development in human fetal, newborn and pediatric brains | 2006 | review | No |
| 975 | Relationship Between Integrity of the Corpus Callosum and Bimanual Coordination in Children With Unilateral Spastic Cerebral Palsy | 2019 | Duplicate Article | No |
| 976 | Transient dysautonomia in an acute phase of encephalopathy with biphasic seizures and late reduced diffusion | 2017 | Duplicate Article | No |
| 977 | Early Environmental Enrichment Enhances Abnormal Brain Connectivity in a Rabbit Model of Intrauterine Growth Restriction | 2018 | Title and abstract clearly do not meet inclusion criteria | No |
| 978 | Neurodevelopmental Effects of Undernutrition and Placental Underperfusion in Fetal Growth Restriction Rabbit Models | 2017 | Title and abstract clearly do not meet inclusion criteria | No |
| 979 | Semi-automated segmentation of the lateral periventricular regions using diffusion magnetic resonance imaging | 2020 | Title and abstract clearly do not meet inclusion criteria | No |
| 980 | Microstructural Periventricular White Matter Injury in Post-hemorrhagic Ventricular Dilatation | 2022 | Title and abstract clearly do not meet inclusion criteria | No |
| 981 | MR diffusion changes in the perimeter of the lateral ventricles demonstrate periventricular injury in post-hemorrhagic hydrocephalus of prematurity | 2019 | Title and abstract clearly do not meet inclusion criteria | No |
| 982 | Clinicoradiological changes of brain NK/T cell lymphoma manifesting pure akinesia: a case report | 2011 | Title and abstract clearly do not meet inclusion criteria | No |
| 983 | Using Diffusion Tensor Imaging Based Measurements to Predict Outcomes of Constraint Induced Movement Therapy in Children with Hemiplegic Cerebral Palsy | 2017 | Title and abstract clearly do not meet inclusion criteria | No |
| 984 | Using Diffusion Tensor Imaging Based Measurements to Predict Outcomes of Constraint Induced Movement Therapy in Children with Hemiplegic Cerebral Palsy | 2017 | Duplicate Article | No |
| 985 | Giant axonal neuropathy A clinicoradiopathologic diagnosis | 2014 | Title and abstract clearly do not meet inclusion criteria | No |
| 986 | Diffusion tensor brain imaging and tractography | 2002 | Duplicate Article | No |
| 987 | Diffusion tensor analysis of corpus callosum in progressive supranuclear palsy | 2008 | Duplicate Article | No |
| 988 | Periventricular low intensities on fluid attenuated inversion recovery imaging in the newborn infant: Relationships to chronic white matter lesions | 2004 | Title and abstract clearly do not meet inclusion criteria | No |
| 989 | Rehabilitation-induced brain changes detected through magnetic resonance imaging in children with neurodevelopmental disorders: A systematic review | 2019 | review | No |
| 990 | Limb-specific thalamocortical tracts are impaired differently in hemiplegic and diplegic subtypes of cerebral palsy | 2023 | No correlation analysis | No |
| 991 | Altered corpus callosum structure in adolescents with cerebral palsy: connection to gait and balance | 2023 | Duplicate Article | No |
| 992 | Brain structural and functional connectivity and network organization in cerebral palsy: A scoping review | 2023 | review | No |
| 993 | Retinal ganglion cell topography predicts visual field function in spastic cerebral palsy | 2020 | Duplicate Article | No |
| 994 | Diffusion Tensor Imaging Tractography Detecting Isolated Oculomotor Nerve Damage After Traumatic Brain Injury | 2017 | Duplicate Article | No |
| 995 | The contribution of the clinical examination, electroencephalogram, and brain MRI in assessing the prognosis in term newborns with neonatal encephalopathy. A cohort of 30 newborns before the introduction of treatment with hypothermia | 2014 | Title and abstract clearly do not meet inclusion criteria | No |
| 996 | Aberrant Pyramidal Tract in Comparison with Pyramidal Tract on Diffusion Tensor Tractography: A Mini-Review | 2017 | Duplicate Article | No |
| 997 | Fetal and Neonatal Effects of N-Acetylcysteine When Used for Neuroprotection in Maternal Chorioamnionitis | 2016 | Duplicate Article | No |
| 998 | Dissecting Aneurysm of Vertebral Artery Manifestating as Contralateral Abducens Nerve Palsy | 2013 | Duplicate Article | No |
| 999 | Early Diagnosis of Spastic Cerebral Palsy in Infants with Periventricular White Matter Injury Using Diffusion Tensor Imaging | 2019 | Duplicate Article | No |
| 1000 | Specific White Matter Lesions Related to Motor Dysfunction in Spastic Cerebral Palsy: A Meta-analysis of Diffusion Tensor Imaging Studies | 2020 | Duplicate Article | No |
| 1001 | Structural network performance for early diagnosis of spastic cerebral palsy in periventricular white matter injury | 2021 | Duplicate Article | No |
| 1002 | A comparison of microstructural maturational changes of the corpus callosum in preterm and full-term children: a diffusion tensor imaging study | 2012 | No correlation analysis | No |
| 1003 | Diffusion tensor imaging of white matter and developmental outcome | 2008 | Duplicate Article | No |
| 1004 | Microstructure of transcallosal motor fibers reflects type of cortical (re-)organization in congenital hemiparesis | 2014 | No correlation analysis | No |
| 1005 | Medial Lemniscus Lesion in Pediatric Hemiplegic Patients without Corticospinal Tract and Posterior Thalamic Radiation Lesion | 2012 | Duplicate Article | No |
| 1006 | Gray Matter Abnormalities in Idiopathic Parkinson's Disease: Evaluation by Diffusional Kurtosis Imaging and Neurite Orientation Dispersion and Density Imaging | 2017 | Title and abstract clearly do not meet inclusion criteria | No |
| 1007 | Early Diffusion-Weighted Images in Infants With Subcortical Leukomalacia | 2010 | Duplicate Article | No |
| 1008 | Brain structural and microstructural alterations associated with cerebral palsy and motor impairments in adolescents born extremely preterm and/or extremely low birthweight | 2015 | No correlation analysis | No |
| 1009 | Axon Density and Axon Orientation Dispersion in Children Born Preterm | 2016 | No correlation analysis | No |
| 1010 | White matter tracts related to memory and emotion in very preterm children | 2021 | No correlation analysis | No |
| 1011 | White Matter NAA/Cho and Cho/Cr Ratios at MR Spectroscopy Are Predictive of Motor Outcome in Preterm Infants | 2014 | Title and abstract clearly do not meet inclusion criteria | No |
| 1012 | Altered diffusion in the frontal lobe in Parkinson disease | 2008 | Title and abstract clearly do not meet inclusion criteria | No |
| 1013 | Neurodevelopmental impairment is associated with altered white matter development in a cohort of school-aged children born very preterm | 2021 | Duplicate Article | No |
| 1014 | Different Patterns of Punctate White Matter Lesions in Serially Scanned Preterm Infants | 2014 | Title and abstract clearly do not meet inclusion criteria | No |
| 1015 | Corticospinal Tract Injury Precedes Thalamic Volume Reduction in Preterm Infants with Cystic Periventricular Leukomalacia | 2015 | Title and abstract clearly do not meet inclusion criteria | No |
| 1016 | Pure motor trigeminal neuropathy in a woman with tegmental pontine infarction | 2013 | Duplicate Article | No |
| 1017 | Neonatal diffusion tensor brain imaging predicts later motor outcome in preterm neonates with white matter abnormalities | 2016 | Duplicate Article | No |
| 1018 | Limb Length Discrepancy and Corticospinal Tract Disruption in Hemiplegic Cerebral Palsy | 2022 | Duplicate Article | No |
| 1019 | Chronic fetal hypoxia affects axonal maturation in guinea pigs during development: A longitudinal diffusion tensor imaging and <i>T</i><sub>2</sub> mapping study | 2015 | Title and abstract clearly do not meet inclusion criteria | No |
| 1020 | Motor function outcomes of pediatric patients with hemiplegic cerebral palsy after rehabilitation treatment: a diffusion tensor imaging study | 2015 | Duplicate Article | No |
| 1021 | Activation of less affected corticospinal tract and poor motor outcome in hemiplegic pediatric patients: a diffusion tensor tractography imaging study | 2015 |  | Yes |
| 1022 | Usefulness of diffusion tensor imaging in patients who showed sustained unexplainable clinical symptom of torticollis | 2012 | Title and abstract clearly do not meet inclusion criteria | No |
| 1023 | Modeling Developmental Plasticity After Perinatal Stroke: Defining Central Therapeutic Targets in Cerebral Palsy | 2013 | Duplicate Article | No |
| 1024 | Quantified corticospinal tract diffusion restriction predicts neonatal stroke outcome | 2007 | Non-CP | No |
| 1025 | Association between brain structural network efficiency at term-equivalent age and early development of cerebral palsy in very preterm infants | 2021 | Title and abstract clearly do not meet inclusion criteria | No |
| 1026 | Neurovisualization features of brain anatomy in children with spastic cerebral palsy revealed by magnetic resonance tractography | 2021 | Title and abstract clearly do not meet inclusion criteria | No |
| 1027 | Neuro-imaging characteristics of sensory impairment in cerebral palsy; a systematic review | 2023 | Duplicate Article | No |
| 1028 | Unusual presentation of acute encephalopathy with biphasic seizures and late reduced diffusion in Miller-Dieker syndrome | 2022 | Duplicate Article | No |
| 1029 | Anisotropy of transcallosal motor fibres indicates functional impairment in children with periventricular leukomalacia | 2011 | Duplicate Article | No |
| 1030 | The importance of MRI tractography in the examination of adult patients with cerebral palsy | 2020 | Title and abstract clearly do not meet inclusion criteria | No |
| 1031 | Sensory Tractography and Robot-Quantified Proprioception in Hemiparetic Children with Perinatal Stroke | 2017 | Duplicate Article | No |
| 1032 | Corticospinal tract diffusion properties and robotic visually guided reaching in children with hemiparetic cerebral palsy | 2018 | Duplicate Article | No |
| 1033 | Dyskinesia Impairment Scale scores in Dutch pre-school children after neonatal therapeutic hypothermia | 2020 | Non-CP | No |
| 1034 | Serial evaluation of swallowing function in a long-term survivor of V180I genetic Creutzfeldt-Jakob disease | 2020 | Duplicate Article | No |
| 1035 | Using diffusion tensor imaging to identify corticospinal tract projection patterns in children with unilateral spastic cerebral palsy | 2017 | Duplicate Article | No |
| 1036 | Early-onset or rapidly progressive scoliosis in children: Check the eyes! | 2013 | Duplicate Article | No |
| 1037 | Injury to the Cerebellum in Term Asphyxiated Newborns Treated with Hypothermia | 2015 | Title and abstract clearly do not meet inclusion criteria | No |
| 1038 | Changes in diffusion tensor tractographic findings associated with constraint-induced movement therapy in young children with cerebral palsy | 2014 | Duplicate Article | No |
| 1039 | Corticoreticular tract lesion in children with developmental delay presenting with gait dysfunction and trunk instability | 2017 | Duplicate Article | No |
| 1040 | Early diffusion-weighted MRI and <SUP>1</SUP>H-magnetic resonance spectroscopy in asphyxiated full-term neonates | 2005 | Title and abstract clearly do not meet inclusion criteria | No |
| 1041 | Diffusion tensor imaging is associated with motor outcomes of very preterm born children at 11 years of age | 2020 | No correlation analysis | No |
| 1042 | Starting a DBS service for children: It's not the latitude but the attitude - Establishment of the paediatric DBS centre in Northern Finland | 2022 | Duplicate Article | No |
| 1043 | Artery of Percheron infarction: review of literature with a case report | 2015 | Duplicate Article | No |
| 1044 | White matter integrity in dyskinetic cerebral palsy: Relationship with intelligence quotient and executive function | 2017 | Duplicate Article | No |
| 1045 | White matter microstructure and receptive vocabulary in children with cerebral palsy: The role of interhemispheric connectivity | 2023 | Duplicate Article | No |
| 1046 | Frontal interhemispheric structural connectivity, attention, and executive function in children with perinatal stroke | 2022 | Duplicate Article | No |
| 1047 | Altered neonatal white and gray matter microstructure is associated with neurodevelopmental impairments in very preterm infants with high-grade brain injury | 2019 | Title and abstract clearly do not meet inclusion criteria | No |
| 1048 | Changes in a cerebellar peduncle lesion in a patient with Dandy-Walker malformation <i>A diffusion tensor imaging study</i> | 2013 | Title and abstract clearly do not meet inclusion criteria | No |
| 1049 | Analysis of Structure-Function Network Decoupling in the Brain Systems of Spastic Diplegic Cerebral Palsy | 2017 | Duplicate Article | No |
| 1050 | Detection of thinned corticospinal tract and corticoreticular pathway in a patient with a calf circumference discrepancy | 2018 | Title and abstract clearly do not meet inclusion criteria | No |
| 1051 | Motor pathway injury in patients with periventricular leucomalacia and spastic diplegia | 2011 | Duplicate Article | No |
| 1052 | Feasibility and Test-Retest Reliability of an Electroencephalography-Based Brain Mapping System in Children With Cerebral Palsy: A Preliminary Investigation | 2012 | Title and abstract clearly do not meet inclusion criteria | No |
| 1053 | Surgical and molecular considerations in the treatment of pediatric thalamopeduncular tumors | 2017 | Duplicate Article | No |
| 1054 | Diffusion-tensor MR imaging and fiber tractography: A new method of describing aberrant fiber connections in developmental CNS anomalies | 2005 | Duplicate Article | No |
| 1055 | Safety and feasibility of countering neurological impairment by intravenous administration of autologous cord blood in cerebral palsy | 2012 | Duplicate Article | No |
| 1056 | Comparing brain white matter on sequential cranial ultrasound and MRI in very preterm infants | 2008 | Title and abstract clearly do not meet inclusion criteria | No |
| 1057 | Alterations in White Matter Microstructure Are Associated with Goal-Directed Upper-Limb Movement Segmentation in Children Born Extremely Preterm | 2017 | Title and abstract clearly do not meet inclusion criteria | No |
| 1058 | Advanced Fiber Tracking in Early Acquired Brain Injury Causing Cerebral Palsy | 2015 | Duplicate Article | No |
| 1059 | Damage to the Immature Optic Radiation Causes Severe Reduction of the Retinal Nerve Fiber Layer, Resulting in Predictable Visual Field Defects | 2014 | Title and abstract clearly do not meet inclusion criteria | No |
| 1060 | Comparing ultrasound with conventional and diffusion tensor MRI as predictors of cerebral palsy in very low birth weight infants: A longitudinal study | 2004 | Title and abstract clearly do not meet inclusion criteria | No |
| 1061 | A Semi-Supervised Graph Convolutional Network for Early Prediction of Motor Abnormalities in Very Preterm Infants | 2023 | Title and abstract clearly do not meet inclusion criteria | No |
| 1062 | Diffusion tensor imaging assesses white matter injury in neonates with hypoxic-ischemic encephalopathy | 2017 | Non-CP | No |
| 1063 | Brain White Matter Network Measures for Cerebral Palsy | 2018 | Title and abstract clearly do not meet inclusion criteria | No |
| 1064 | Contemporary therapeutic strategies for occlusion of the artery of Percheron: a review of the literature | 2015 | Title and abstract clearly do not meet inclusion criteria | No |
| 1065 | New Insights into the Developing Rabbit Brain Using Diffusion Tensor Tractography and Generalized q-Sampling MRI | 2015 | Title and abstract clearly do not meet inclusion criteria | No |
| 1066 | Clinical seizures and unfavorable brain MRI patterns in neonates with hypoxic ischemic encephalopathy | 2021 | Title and abstract clearly do not meet inclusion criteria | No |
| 1067 | Structure of brain grey and white matter in infants with spastic cerebral palsy and periventricular white matter injury | 2023 | Title and abstract clearly do not meet inclusion criteria | No |
| 1068 | Treatment response prediction of rehabilitation program in children with cerebral palsy using radiomics strategy: protocol for a multicenter prospective cohort study in west China | 2019 | Title and abstract clearly do not meet inclusion criteria | No |
| 1069 | White Matter Abnormalities Are Related to Microstructural Changes in Preterm Neonates at Term-Equivalent Age: A Diffusion Tensor Imaging and Probabilistic Tractography Study | 2012 | Title and abstract clearly do not meet inclusion criteria | No |
| 1070 | Transclival Approach for Resection of a Pontine Cavernous Malformation: 2-Dimensional Operative Video | 2020 | Title and abstract clearly do not meet inclusion criteria | No |
| 1071 | Peak Width of Skeletonized Mean Diffusivity as a Marker of Diffuse Cerebrovascular Damage | 2020 | Duplicate Article | No |
| 1072 | Diffusion tensor imaging of the pyramidal tracts in infants with motor dysfunction | 2008 | Title and abstract clearly do not meet inclusion criteria | No |
| 1073 | Fractional anisotropy in children with dystonia or spasticity correlates with the selection for DBS or ITB movement disorder surgery | 2016 | Title and abstract clearly do not meet inclusion criteria | No |
| 1074 | Deep brain stimulation for childhood dystonia: Is 'where' as important as in 'whom'? | 2017 | Title and abstract clearly do not meet inclusion criteria | No |
| 1075 | Central Motor Conduction Time and Diffusion Tensor Imaging metrics in children with complex motor disorders | 2015 | Title and abstract clearly do not meet inclusion criteria | No |
| 1076 | Neuroimaging in Vascular Parkinsonism | 2019 | Title and abstract clearly do not meet inclusion criteria | No |
| 1077 | Association of Acute Infarct Topography With Development of Cerebral Palsy and Neurologic Impairment in Neonates With Stroke | 2023 | Title and abstract clearly do not meet inclusion criteria | No |
| 1078 | Upper limb function and cortical organization in youth with unilateral cerebral palsy | 2014 | Duplicate Article | No |
| 1079 | Correlation Between Fractional Anisotropy and Motor Outcomes in One-Year-Old Infants With Periventricular Brain Injury | 2014 |  | Yes |
| 1080 | Does motor deficit in children with cerebral palsy correlate with diffusion tensor metrics abnormalities in thalamocortical pathways? | 2021 | Title and abstract clearly do not meet inclusion criteria | No |
| 1081 | Significance of MRI in Diagnosis and Differential Diagnosis of Parkinson's Disease | 2010 | Duplicate Article | No |
| 1082 | The relationship between neuroimaging and motor outcome in children with cerebral palsy: A systematic review-Part B diffusion imaging and tractography | 2020 | Duplicate Article | No |
| 1083 | White matter characteristics of motor, sensory and interhemispheric tracts underlying impaired upper limb function in children with unilateral cerebral palsy | 2020 |  | Yes |
| 1084 | Advanced MRI analysis to detect white matter brain injury in growth restricted newborn lambs | 2019 | Title and abstract clearly do not meet inclusion criteria | No |
| 1085 | Acid-Base Parameters for Predicting Magnetic Resonance Imaging Measures of Neurologic Outcome after Perinatal Hypoxia-Ischemia: Is the Strong Ion Gap Superior to Base Excess and Lactate? | 2012 | Title and abstract clearly do not meet inclusion criteria | No |
| 1086 | Resting State and Diffusion Neuroimaging Predictors of Clinical Improvements Following Constraint-Induced Movement Therapy in Children With Hemiplegic Cerebral Palsy | 2015 | Duplicate Article | No |
| 1087 | Spatial analysis of diffusion tensor tractography statistics along the inferior fronto-occipital fasciculus with application in progressive supranuclear palsy | 2013 | Duplicate Article | No |
| 1088 | MRI Based Preterm White Matter Injury Classification: The Importance of Sequential Imaging in Determining Severity of Injury | 2016 | Title and abstract clearly do not meet inclusion criteria | No |
| 1089 | The Challenge of Diffusion Magnetic Resonance Imaging in Cerebral Palsy: A Proposed Method to Identify White Matter Pathways | 2023 | Duplicate Article | No |
| 1090 | Foix-Chavany-Marie syndrome caused by a disconnection between the right pars opercularis of the inferior frontal gyms and the supplementary motor area Case report | 2012 | Title and abstract clearly do not meet inclusion criteria | No |
| 1091 | The Effects of Low Frequency Repetitive Transcranial Magnetic Stimulation on White Matter Structural Connectivity in Children with Cerebral Palsy | 2018 | Duplicate Article | No |
| 1092 | Neonatal neurobehavior after therapeutic hypothermia for hypoxic ischemic encephalopathy | 2015 | Duplicate Article | No |
| 1093 | Quantitative analysis of magnetic resonance images and neurological outcome in encephalopathic neonates treated with whole-body hypothermia | 2010 | Duplicate Article | No |
| 1094 | Maturation of Corpus Callosum Anterior Midbody Is Associated with Neonatal Motor Function in Eight Preterm-Born Infants | 2013 | Title and abstract clearly do not meet inclusion criteria | No |
| 1095 | Magnetic resonance imaging-Insights into brain injury and outcomes in premature infants | 2009 | Title and abstract clearly do not meet inclusion criteria | No |
| 1096 | Long-Term Neuropathological Changes Associated with Cerebral Palsy in a Nonhuman Primate Model of Hypoxic-Ischemic Encephalopathy | 2017 | Duplicate Article | No |
| 1097 | Central motor conduction studies and diagnostic magnetic resonance imaging in children with severe primary and secondary dystonia | 2011 | Title and abstract clearly do not meet inclusion criteria | No |
| 1098 | Discovering the sense of touch: protocol for a randomised controlled trial examining the efficacy of a somatosensory discrimination intervention for children with hemiplegic cerebral palsy | 2018 | Duplicate Article | No |
| 1099 | Quantitative MRI Characterization of the Extremely Preterm Brain at Adolescence: Atypical versus Neurotypical Developmental Pathways | 2022 | Title and abstract clearly do not meet inclusion criteria | No |
| 1100 | Practice parameter: Neuroimaging of the neonate - Report of the Quality Standards Subcommittee of the American Academy of Neurology and the Practice Committee of the Child Neurology Society | 2002 | Title and abstract clearly do not meet inclusion criteria | No |
| 1101 | The structural connectome in children: basic concepts, how to build it, and synopsis of challenges for the developing pediatric brain | 2017 | Title and abstract clearly do not meet inclusion criteria | No |
| 1102 | Neonatal Functional and Structural Connectivity Are Associated with Cerebral Palsy at Two Years of Age | 2020 | Duplicate Article | No |
| 1103 | Macrostructural and Microstructural Brain Lesions Relate to Gait Pathology in Children With Cerebral Palsy | 2016 | Duplicate Article | No |
| 1104 | Umbilical Cord Blood Therapy Potentiated with Erythropoietin for Children with Cerebral Palsy: A Double-blind, Randomized, Placebo-Controlled Trial | 2013 | Duplicate Article | No |
| 1105 | Potentiation of cord blood cell therapy with erythropoietin for children with CP: a 2x2 factorial randomized placebo-controlled trial | 2020 | Title and abstract clearly do not meet inclusion criteria | No |
| 1106 | Reliability of Fractional Anisotropy Measurement for Children with Cerebral Palsy | 2014 | Duplicate Article | No |
| 1107 | SARS-CoV-19-associated Rhino-orbital and cerebral mucormycosis: clinical and radiological presentations | 2022 | Title and abstract clearly do not meet inclusion criteria | No |
| 1108 | Magnetic resonance imaging pattern recognition in childhood bilateral basal ganglia disorders | 2020 | Title and abstract clearly do not meet inclusion criteria | No |
| 1109 | Acute pseudobulbar paralysis:: The use of diffusion techniques with magnetic resonance | 1999 | Duplicate Article | No |
| 1110 | Basal ganglia infarction and hemorrhage associated with subarachnoid hemorrhage after trauma in an adult patient: A case report | 2023 | Title and abstract clearly do not meet inclusion criteria | No |
| 1111 | Low-grade intraventricular hemorrhage disrupts cerebellar white matter in preterm infants: evidence from diffusion tensor imaging | 2015 | Title and abstract clearly do not meet inclusion criteria | No |
| 1112 | The Role of the Corpus Callosum in Pediatric Dysphagia: Preliminary Findings from a Diffusion Tensor Imaging Study in Children with Unilateral Spastic Cerebral Palsy | 2017 | Duplicate Article | No |
| 1113 | Spatial Patterns of Whole Brain Grey and White Matter Injury in Patients with Occult Spastic Diplegic Cerebral Palsy | 2014 | Title and abstract clearly do not meet inclusion criteria | No |
| 1114 | Quantitative Cranial Magnetic Resonance Imaging in Neonatal Hypoxic-Ischemic Encephalopathy | 2012 | Duplicate Article | No |
| 1115 | Fiber-tracking techniques can predict the degree of neurologic impairment for periventricular leukomalacia | 2008 | Duplicate Article | No |
| 1116 | White matter microstructure is associated with language in children born very preterm | 2018 | Title and abstract clearly do not meet inclusion criteria | No |
| 1117 | Diffusion tensor imaging in children with periventricular leukomalacia: Variability of injuries to white matter tracts | 2007 | Title and abstract clearly do not meet inclusion criteria | No |
| 1118 | Diffusion tensor imaging on teenagers, born at term with moderate hypoxic-ischemic encephalopathy | 2005 | Duplicate Article | No |
| 1119 | Recent advances in the use of MRI to assess early human cortical development | 2018 | Title and abstract clearly do not meet inclusion criteria | No |
| 1120 | Neurite orientation dispersion and density imaging quantifies corticospinal tract microstructural organization in children with unilateral cerebral palsy | 2019 | Duplicate Article | No |
| 1121 | The brain's kryptonite: Overview of punctate white matter lesions in neonates | 2019 | Title and abstract clearly do not meet inclusion criteria | No |
| 1122 | Magnetic Resonance Imaging in Tauopathy Animal Models | 2022 | Title and abstract clearly do not meet inclusion criteria | No |
| 1123 | Artery of Percheron Infarct: A Rarity Not to be Missed | 2017 | Title and abstract clearly do not meet inclusion criteria | No |
| 1124 | Empyema necessitatis due to <i>Pseudomonas aeruginosa</i> in a child with cerebral palsy | 2020 | Title and abstract clearly do not meet inclusion criteria | No |
| 1125 | Speech and Oromotor Outcome in Adolescents Born Preterm: Relationship to Motor Tract Integrity | 2012 | Title and abstract clearly do not meet inclusion criteria | No |
| 1126 | Detection of Botulinum Toxin Muscle Effect in Humans Using Magnetic Resonance Imaging: A Qualitative Case Series | 2017 | Title and abstract clearly do not meet inclusion criteria | No |
| 1127 | The utility of the fronto-temporal horn ratio on cranial ultrasound in premature newborns: a ventriculomegaly marker | 2021 | Duplicate Article | No |
| 1128 | Clinical study of six patients with pure dysarthria and dysarthria-(Central) facial nerve palsy/isolated central facial nerve palsy caused by extracerebellar infarction | 2023 | Title and abstract clearly do not meet inclusion criteria | No |
| 1129 | Apparent diffusion coefficient measurements in progressive supranuclear palsy | 2000 | Duplicate Article | No |
| 1130 | Quantitative evaluation of brain development using anatomical MRI and diffusion tensor imaging | 2013 | Duplicate Article | No |
| 1131 | Quantitative evaluation of brain development using anatomical MRI and diffusion tensor imaging (Reprinted from International Journal of Developmental Neuroscience, vol 31, pg 512-524, 2013) | 2014 | Title and abstract clearly do not meet inclusion criteria | No |
| 1132 | Low signal intensity and increased anisotropy on magnetic resonance imaging in the white matter lesion after head trauma: Unrecognized findings of diffuse axonal injury | 2007 | Duplicate Article | No |
| 1133 | Diffusion tensor imaging in infants with basal ganglia-thalamic lesions | 2008 | Duplicate Article | No |
| 1134 | Diffusion Tensor Imaging in Arginase Deficiency Reveals Damage to Corticospinal Tracts | 2010 | Duplicate Article | No |
| 1135 | Creutzfeldt-Jakob Disease: Atypical Presentation of a Very Rare Disease | 2021 | Duplicate Article | No |
| 1136 | THREE PATTERNS OF MIRROR MOVEMENTS OF THE HAND IN HEMIPLEGIC CEREBRAL PALSY EVALUATED BY FUNCTIONAL MRI AND DIFFUSION TENSOR IMAGING | 2010 | Title and abstract clearly do not meet inclusion criteria | No |
| 1137 | Visual Perception in Preterm Children: What Are We Currently Measuring? | 2011 | Duplicate Article | No |
| 1138 | Voxel-based analysis derived from fractional anisotropy images of white matter volume changes with aging | 2008 | Title and abstract clearly do not meet inclusion criteria | No |
| 1139 | Neuroimaging biomarkers of preterm brain injury: toward developing the preterm connectome | 2012 | Duplicate Article | No |
| 1140 | Assessment of the structural brain network reveals altered connectivity in children with unilateral cerebral palsy due to periventricular white matter lesions | 2014 | Duplicate Article | No |
| 1141 | Fixel-based analysis reveals alterations is brain microstructure and macrostructure of preterm-born infants at term equivalent age | 2018 | Duplicate Article | No |
| 1142 | Brain microstructure and morphology of very preterm-born infants at term equivalent age: Associations with motor and cognitive outcomes at 1 and 2 years | 2020 | Duplicate Article | No |
| 1143 | Assessment of Structural Connectivity in the Preterm Brain at Term Equivalent Age Using Diffusion MRI and T<sub>2</sub> Relaxometry: A Network-Based Analysis | 2013 | Title and abstract clearly do not meet inclusion criteria | No |
| 1144 | Magnetic resonance diffusion tractography of the preterm infant brain: a systematic review | 2014 | Duplicate Article | No |
| 1145 | Altered White Matter Connectivity Associated with Intergyral Brain Disorganization in Hemiplegic Cerebral Palsy | 2019 | Duplicate Article | No |
| 1146 | Cortical somatosensory reorganization in children with spastic cerebral palsy: a multimodal neuroimaging study | 2014 | Duplicate Article | No |
| 1147 | Reorganization of the somatosensory cortex in hemiplegic cerebral palsy associated with impaired sensory tracts | 2018 | Duplicate Article | No |
| 1148 | Maturation of Corticospinal Tracts in Children With Hemiplegic Cerebral Palsy Assessed by Diffusion Tensor Imaging and Transcranial Magnetic Stimulation | 2019 | Duplicate Article | No |
| 1149 | Diffusion MRI Microstructural Abnormalities at Term-Equivalent Age Are Associated with Neurodevelopmental Outcomes at 3 Years of Age in Very Preterm Infants | 2021 | Title and abstract clearly do not meet inclusion criteria | No |
| 1150 | Advanced neuroimaging and its role in predicting neurodevelopmental outcomes in very preterm infants | 2016 | Duplicate Article | No |
| 1151 | Early Detection of Cerebral Palsy Using Sensorimotor Tract Biomarkers in Very Preterm Infants | 2019 | Duplicate Article | No |
| 1152 | Brain PET, MRI and tractography in cerebral palsy | 2005 | Title and abstract clearly do not meet inclusion criteria | No |
| 1153 | Brain PET, MRI and tractography in cerebral palsy | 2005 | Duplicate Article | No |
| 1154 | Diffusion tensor imaging with tractography and GABA<sub>A</sub> receptor imaging in cerebral palsy | 2007 | Title and abstract clearly do not meet inclusion criteria | No |
| 1155 | Increased GABA-A Receptor Binding and Reduced Connectivity at the Motor Cortex in Children with Hemiplegic Cerebral Palsy: A Multimodal Investigation Using <SUP>18</SUP>F-Fluoroflumazenil PET, Immunohistochemistry, and MR Imaging | 2013 | Duplicate Article | No |
| 1156 | Different clinical courses of various radiologic findings in fibromuscular dysplasia during a 7-year follow-up A case report | 2020 | Duplicate Article | No |
| 1157 | Rapid Regression of Carotid Artery Stenosis Shortly after Intensive Medical Therapy | 2022 | Duplicate Article | No |
| 1158 | Cranial MRI beyond the Neonatal Period and Neurodevelopmental Outcomes in Neonatal Encephalopathy Due to Perinatal Asphyxia: A Systematic Review | 2023 | Non-CP | No |
| 1159 | Magnetic resonance imaging markers of Parkinson's disease nigrostriatal signature | 2010 | Title and abstract clearly do not meet inclusion criteria | No |
| 1160 | Neural correlates of developmental coordination disorder | 2013 | Duplicate Article | No |
| 1161 | Ischemic stroke of the cortical "hand knob" area: stroke mechanisms and prognosis | 2009 | Title and abstract clearly do not meet inclusion criteria | No |
| 1162 | White Matter Injury and General Movements in High-Risk Preterm Infants | 2017 | Duplicate Article | No |
| 1163 | Multimodal assessment of motor pathways and intracortical connections in functional hemispherectomy | 2020 | Duplicate Article | No |
| 1164 | Role of Diffusion Tensor Imaging as an Independent Predictor of Cognitive and Language Development in Extremely Low-Birth-Weight Infants | 2014 | Duplicate Article | No |
| 1165 | RETRACTED: Effect of antenatal magnesium sulphate on MRI biomarkers of white matter development at term equivalent age: The magnum study (Retracted article. See vol. 78, 2022) | 2020 | Title and abstract clearly do not meet inclusion criteria | No |
| 1166 | Deep Brain Stimulation and Hypoxemic Perinatal Encephalopathy: State of Art and Perspectives | 2021 | Duplicate Article | No |
| 1167 | Structural brain connectivity in children after neonatal stroke: A whole-brain fixel-based analysis | 2022 | Non-CP | No |
| 1168 | Exploring structural connectomes in children with unilateral cerebral palsy using graph theory | 2023 | Duplicate Article | No |
| 1169 | Neuroregenerative potential of intravenous G-CSF and autologous peripheral blood stem cells in children with cerebral palsy: a randomized, double-blind, cross-over study | 2017 | Duplicate Article | No |
| 1170 | DTI correlates of cognition in term children with spastic diplegic cerebral palsy | 2013 | Duplicate Article | No |
| 1171 | Cerebral venous thrombosis, intraventricular haemorrhage and white matter lesions in a preterm newborn with factor V (Leiden) mutation | 2002 | Title and abstract clearly do not meet inclusion criteria | No |
| 1172 | MR Imaging of hypoxic ischemic encephalopathy - Distribution Patterns and ADC value correlations | 2018 | Duplicate Article | No |
| 1173 | Therapeutic Effects of an Anti-Gravity Locomotor Training (AlterG) on Postural Balance and Cerebellum Structure in Children with Cerebral Palsy | 2017 | Duplicate Article | No |
| 1174 | Diffusion microstructure imaging in progressive supranuclear palsy: reduced axonal volumes in the superior cerebellar peduncles, dentato-rubro-thalamic tracts, ventromedial thalami, and frontomesial white matter | 2022 | Non-CP | No |
| 1175 | Widespread white matter oedema in subacute COVID-19 patients with neurological symptoms | 2022 | Title and abstract clearly do not meet inclusion criteria | No |
| 1176 | Changes in White Matter Integrity following Intensive Voice Treatment (LSVT LOUD®) in Children with Cerebral Palsy and Motor Speech Disorders | 2017 | Duplicate Article | No |
| 1177 | Surface-Based fMRI-Driven Diffusion Tractography in the Presence of Significant Brain Pathology: A Study Linking Structure and Function in Cerebral Palsy | 2016 | Duplicate Article | No |
| 1178 | Surface-Based fMRI-Driven Diffusion Tractography in the Presence of Significant Brain Pathology: A Study Linking Structure and Function in Cerebral Palsy (vol 11, e0159540, 2016) | 2016 | Title and abstract clearly do not meet inclusion criteria | No |
| 1179 | Measuring neuroplasticity associated with cerebral palsy rehabilitation: An MRI based power analysis | 2017 | Duplicate Article | No |
| 1180 | Midbrain and bilateral paramedian thalamic stroke due to artery of Percheron occlusion | 2016 | Duplicate Article | No |
| 1181 | Comparing quantitative tractography metrics of motor and sensory pathways in children with periventricular leukomalacia and different levels of gross motor function | 2012 | Duplicate Article | No |
| 1182 | Detection of Focal Cerebral Injury using Diffusion Tensor Magnetic Resonance imaging in a Boy with Becker Muscular Dystrophy | 2009 | Duplicate Article | No |
| 1183 | Microstructural white matter tract alteration in Prader-Willi syndrome: A diffusion tensor imaging study | 2017 | review | No |
| 1184 | Diffusion Tensor Imaging Study of the Response to Constraint-Induced Movement Therapy of Children With Hemiparetic Cerebral Palsy and Adults With Chronic Stroke | 2014 | Duplicate Article | No |
| 1185 | Diffusion-weighted brain imaging study of patients with clinical diagnosis of corticobasal degeneration, progressive supranuclear palsy and Parkinson's disease | 2008 | Duplicate Article | No |
| 1186 | Brain Diffusion-Weighted Imaging in Friedreich's Ataxia | 2011 | Title and abstract clearly do not meet inclusion criteria | No |
| 1187 | Brain MR Contribution to the Differential Diagnosis of Parkinsonian Syndromes: An Update | 2016 | Title and abstract clearly do not meet inclusion criteria | No |
| 1188 | Intensive Bimanual Intervention for Children Who Have Undergone Hemispherectomy: A Pilot Study | 2021 | Non-CP | No |
| 1189 | Corpus Callosum Integrity Relates to Improvement of Upper-Extremity Function Following Intensive Rehabilitation in Children With Unilateral Spastic Cerebral Palsy | 2021 | Duplicate Article | No |
| 1190 | Neonatal erythropoietin mitigates impaired gait, social interaction and diffusion tensor imaging abnormalities in a rat model of prenatal brain injury | 2018 | Duplicate Article | No |
| 1191 | MRI Predicts Efficacy of Constraint-Induced Movement Therapy in Children With Brain Injury | 2013 | No correlation analysis | No |
| 1192 | Comparison of neonatal MRI examinations with and without an MR-compatible incubator: Advantages in examination feasibility and clinical decision-making | 2010 | Title and abstract clearly do not meet inclusion criteria | No |
| 1193 | Neonatal brain structure on MRI and diffusion tensor imaging, sex, and neurodevelopment in very-low-birthweight preterm children | 2009 | Duplicate Article | No |
| 1194 | Movement disorders due to bilirubin toxicity | 2007 | Duplicate Article | No |
| 1195 | Movement disorders due to bilirubin toxicity | 2015 | Duplicate Article | No |
| 1196 | Neonatal physiological correlates of near-term brain development on MRI and DTI in very-low-birth-weight preterm infants | 2014 | Title and abstract clearly do not meet inclusion criteria | No |
| 1197 | Neonatal DTI early after birth predicts motor outcome in preterm infants with periventricular hemorrhagic infarction | 2015 | Duplicate Article | No |
| 1198 | The extent of intrauterine growth restriction determines the severity of cerebral injury and neurobehavioural deficits in rodents | 2017 | Title and abstract clearly do not meet inclusion criteria | No |
| 1199 | Magnetic resonance imaging of white matter diseases of prematurity | 2010 | review | No |
| 1200 | Intrauterine Endotoxin Administration Leads to White Matter Diffusivity Changes in Newborn Rabbits | 2009 | Title and abstract clearly do not meet inclusion criteria | No |
| 1201 | Evaluation of Role of Magnetic Resonance Imagine in Newborns with Suspected Hypoxic Ischaemic Injury and its Association with Clinical Staging: A Cross-sectional Study | 2022 | Non-CP | No |
| 1202 | Imaging biomarkers in Parkinson's disease and Parkinsonian syndromes: current and emerging concepts | 2017 | Duplicate Article | No |
| 1203 | Neuroimaging Advances in Parkinson's Disease and Atypical Parkinsonian Syndromes | 2020 | Duplicate Article | No |
| 1204 | Predicting motor outcome in preterm infants from very early brain diffusion MRI using a deep learning convolutional neural network (CNN) model | 2020 | Duplicate Article | No |
| 1205 | Investigating Brain Age Deviation in Preterm Infants: A Deep Learning Approach | 2018 | Title and abstract clearly do not meet inclusion criteria | No |
| 1206 | Radiological Imaging in Ataxia Telangiectasia: a Review | 2014 | Title and abstract clearly do not meet inclusion criteria | No |
| 1207 | Soleus muscle weakness in cerebral palsy: Muscle architecture revealed with Diffusion Tensor Imaging | 2019 | Duplicate Article | No |
| 1208 | Soleus muscle weakness in cerebral palsy: Muscle architecture revealed with Diffusion Tensor Imaging (vol 14, e0205944, 2019) | 2020 | Duplicate Article | No |
| 1209 | Isolated cerebral mucormycosis: A case discussion | 2023 | Duplicate Article | No |
| 1210 | In vivo evaluation of white matter pathology in patients of progressive supranuclear palsy using TBSS | 2012 | Duplicate Article | No |
| 1211 | Imaging Findings in Intracranial Aspergillus Infection in Immunocompetent Patients | 2010 | Title and abstract clearly do not meet inclusion criteria | No |
| 1212 | Six Months Guided Exercise Therapy Improves Motor Abilities and White Matter Connectivity in Children with Cerebral Palsy | 2020 | Duplicate Article | No |
| 1213 | Characterisation of the Corticospinal Tract Using Diffusion Magnetic Resonance Imaging in Unilateral and Bilateral Cerebral Palsy Patients | 2018 | Duplicate Article | No |
| 1214 | Experimental cerebral palsy causes microstructural brain damage in areas associated to motor deficits but no spatial memory impairments in the developing rat | 2021 | Duplicate Article | No |
| 1215 | Can Deep Learning Hit a Moving Target? A Scoping Review of Its Role to Study Neurological Disorders in Children | 2021 | review | No |
| 1216 | Corticospinal tract abnormalities and ventricular dilatation: A transdiagnostic comparative tractography study | 2021 | Duplicate Article | No |
| 1217 | Normal diffusion-weighted imaging in cerebral air embolism complicating angiography | 2000 | Title and abstract clearly do not meet inclusion criteria | No |
| 1218 | New insights into the pathology of white matter tracts in cerebral palsy from diffusion magnetic resonance imaging: a systematic review | 2012 | Duplicate Article | No |
| 1219 | Extent of altered white matter in unilateral and bilateral periventricular white matter lesions in children with unilateral cerebral palsy | 2016 | Duplicate Article | No |
| 1220 | Structural connectivity of the anterior cingulate in children with unilateral cerebral palsy due to white matter lesions | 2015 | Duplicate Article | No |
| 1221 | Imaging Predictors of Improvement From a Motor Learning-Based Intervention for Children With Unilateral Cerebral Palsy | 2016 | Duplicate Article | No |
| 1222 | Adolescent hyperactivity and impaired coordination after neonatal hyperoxia | 2012 | Title and abstract clearly do not meet inclusion criteria | No |
| 1223 | Potential Role of High-Field MRI for Studies in Parkinson's Disease | 2009 | Title and abstract clearly do not meet inclusion criteria | No |
| 1224 | Midbrain ischemia presenting as vertical gaze palsy: Value of diffusion-weighted magnetic resonance Imaging | 2004 | Duplicate Article | No |
| 1225 | MRI for the differential diagnosis of neurodegenerative parkinsonism in clinical practice | 2007 | Title and abstract clearly do not meet inclusion criteria | No |
| 1226 | Brain Magnetic Resonance Imaging Techniques in the Diagnosis of Parkinsonian Syndromes | 2010 | Duplicate Article | No |
| 1227 | An update on conventional and advanced magnetic resonance imaging techniques in the differential diagnosis of neurodegenerative parkinsonism | 2005 | Title and abstract clearly do not meet inclusion criteria | No |
| 1228 | Patterns of excitotoxin-induced brain lesions in the newborn rabbit: A neuropathological and MRI correlation | 2005 | Title and abstract clearly do not meet inclusion criteria | No |
| 1229 | Diffusion Tensor Tractography of the Cerebellar Peduncles in Prematurely Born 7-Year-Old Children | 2017 | Title and abstract clearly do not meet inclusion criteria | No |
| 1230 | Therapeutic N-Acetyl-Cysteine (Nac) Following Initiation of Maternal Inflammation Attenuates Long-Term Offspring Cerebral Injury, as Evident in Magnetic Resonance Imaging (MRI) | 2019 | Title and abstract clearly do not meet inclusion criteria | No |
| 1231 | Selective hand motor cortex lesions masquerading as "Pseudoperipheral Nerve Palsy" | 2020 | Duplicate Article | No |
| 1232 | A SPATIO-TEMPORAL ATLAS OF NEONATAL DIFFUSION MRI BASED ON KERNEL RIDGE REGRESSION | 2017 | Title and abstract clearly do not meet inclusion criteria | No |
| 1233 | Imaging for Diagnosis and Treatment of Cerebral Palsy | 2008 | Duplicate Article | No |
| 1234 | MRI-Based Radiologic Scoring System for Extent of Brain Injury in Children with Hemiplegia | 2014 | Duplicate Article | No |
| 1235 | Sex Differences in Outcome and Associations with Neonatal Brain Morphology in Extremely Preterm Children | 2014 | Duplicate Article | No |
| 1236 | Neonatal brain injury and aberrant connectivity | 2019 | review | No |
| 1237 | Cortical Gray and Adjacent White Matter Demonstrate Synchronous Maturation in Very Preterm Infants | 2016 | Title and abstract clearly do not meet inclusion criteria | No |
| 1238 | Assessment of the corticospinal fiber integrity in mirror movement disorder | 2018 | review | No |
| 1239 | Diffusion tensor imaging demonstrates focal lesions of the corticospinal tract in hemiparetic patients with cerebral palsy | 2007 | Duplicate Article | No |
| 1240 | Improvement of Gait Dysfunction after Applying a Hinged Ankle-Foot Orthosis in a Hemiplegic Cerebral Palsy Patient with Disrupted Medial Lemniscus: A Case Report | 2021 | Duplicate Article | No |
| 1241 | Diffusion tensor tractography can predict hemiparesis in infants with high risk factors | 2009 | Duplicate Article | No |
| 1242 | Motor function and white matter connectivity in children cooled for neonatal encephalopathy | 2021 | Duplicate Article | No |
| 1243 | Disrupted brain connectivity in children treated with therapeutic hypothermia for neonatal encephalopathy | 2021 | Duplicate Article | No |
| 1244 | Mammillary body abnormalities and cognitive outcomes in children cooled for neonatal encephalopathy | 2023 | Duplicate Article | No |
| 1245 | Diffusion Imaging of Cerebral Diaschisis in Neonatal Arterial Ischemic Stroke | 2019 | Duplicate Article | No |
| 1246 | Investigations on the plasticity of the childhood brain | 2007 | Title and abstract clearly do not meet inclusion criteria | No |
| 1247 | Tensor and non-tensor tractography for the assessment of the corticospinal tract of children with motor disorders: a comparative study | 2016 | Title and abstract clearly do not meet inclusion criteria | No |
| 1248 | Cortical gray and subcortical white matter associations in Parkinson's disease | 2017 | Title and abstract clearly do not meet inclusion criteria | No |
| 1249 | Delayed Neural Network Degeneration after Neonatal Hypoxia-Ischemia | 2008 | Title and abstract clearly do not meet inclusion criteria | No |
| 1250 | DiffusionTensor Imaging to Predict Neurodevelopmental Impairment in Infants after Hypoxic-Ischemic Injury | 2023 | Duplicate Article | No |
| 1251 | Widespread diffusion changes differentiate Parkinson's disease and progressive supranuclear palsy | 2018 | Duplicate Article | No |
| 1252 | Nutritive sucking abnormalities and brain microstructural abnormalities in infants with established brain injury: a pilot study | 2019 | Title and abstract clearly do not meet inclusion criteria | No |
| 1253 | Clinical features and imaging markers of small vessel disease in symptomatic acute subcortical cerebral microinfarcts | 2022 | Title and abstract clearly do not meet inclusion criteria | No |
| 1254 | A whole-brain analysis in de novo Parkinson disease | 2008 | Title and abstract clearly do not meet inclusion criteria | No |
| 1255 | Fine Motor Skill Mediates Visual Memory Ability with Microstructural Neuro-correlates in Cerebellar Peduncles in Prematurely Born Adolescents | 2017 | Non-CP | No |
| 1256 | Quantitative diffusion tensor imaging in cerebral palsy due to periventricular white matter injury | 2005 | Title and abstract clearly do not meet inclusion criteria | No |
| 1257 | Structural connectivity relates to perinatal factors and functional impairment at 7 years in children born very preterm | 2016 | Title and abstract clearly do not meet inclusion criteria | No |
| 1258 | Characterization of the corpus callosum in very preterm and full-term infants utilizing MRI | 2011 | Title and abstract clearly do not meet inclusion criteria | No |
| 1259 | Corpus callosum alterations in very preterm infants: Perinatal correlates and 2 year neurodevelopmental outcomes | 2012 | Title and abstract clearly do not meet inclusion criteria | No |
| 1260 | Regional white matter microstructure in very preterm infants: Predictors and 7 year outcomes | 2014 | Title and abstract clearly do not meet inclusion criteria | No |
| 1261 | Accelerated Corpus Callosum Development in Prematurity Predicts Improved Outcome | 2015 | Title and abstract clearly do not meet inclusion criteria | No |
| 1262 | Alterations in the optic radiations of very preterm children-Perinatal predictors and relationships with visual outcomes | 2014 | Non-CP | No |
| 1263 | Concurrent Erythropoietin and Hypothermia Treatment Improve Outcomes in a Term Nonhuman Primate Model of Perinatal Asphyxia | 2013 | Duplicate Article | No |
| 1264 | Correlation of quantitative sensorimotor tractography with clinical grade of cerebral palsy | 2010 | Duplicate Article | No |
| 1265 | Treatment-Induced Plasticity in Cerebral Palsy: A Diffusion Tensor Imaging Study | 2008 | Duplicate Article | No |
| 1266 | Changes in the integrity of thalamocortical connections are associated with sensorimotor deficits in children with congenital hemiplegia | 2015 | Duplicate Article | No |
| 1267 | Reduced integrity of sensorimotor projections traversing the posterior limb of the internal capsule in children with congenital hemiparesis | 2014 | Duplicate Article | No |
| 1268 | Skew Deviation: Case Report and Review of the Literature | 2017 | Duplicate Article | No |
| 1269 | Differential progression of magnetization transfer imaging changes depending on severity of cerebral hypoxic-ischemic injury | 2008 | Title and abstract clearly do not meet inclusion criteria | No |
| 1270 | Prediction of neurodevelopmental outcome after hypoxic-ischemic encephalopathy treated with hypothermia by diffusion tensor imaging analyzed using tract-based spatial statistics | 2012 | Non-CP | No |
| 1271 | MR imaging of term infants with hypoxic-ischaemic encephalopathy as a predictor of neurodevelopmental outcome and late MRI appearances | 2010 | Title and abstract clearly do not meet inclusion criteria | No |
| 1272 | Neonatal MRI is associated with future cognition and academic achievement in preterm children | 2015 | Title and abstract clearly do not meet inclusion criteria | No |
| 1273 | Gray and white matter structural changes in corticobasal syndrome | 2016 | Title and abstract clearly do not meet inclusion criteria | No |
| 1274 | Aortic dissection diagnosed on stroke computed tomography protocol: a case report | 2021 | Title and abstract clearly do not meet inclusion criteria | No |
| 1275 | Does Diffusion Tensor Imaging-Based Tractography at 3 Months of Age Contribute to the Prediction of Motor Outcome After Perinatal Arterial Ischemic Stroke? | 2011 | Title and abstract clearly do not meet inclusion criteria | No |
| 1276 | Fiber Tracking at Term Displays Gender Differences Regarding Cognitive and Motor Outcome at 2 Years of Age in Preterm Infants | 2011 | Title and abstract clearly do not meet inclusion criteria | No |
| 1277 | Human parechovirus causes encephalitis with white matter injury in Neonates | 2008 | Title and abstract clearly do not meet inclusion criteria | No |
| 1278 | Mutation in the <i>AP4M1</i> Gene Provides a Model for Neuroaxonal Injury in Cerebral Palsy | 2009 | Title and abstract clearly do not meet inclusion criteria | No |
| 1279 | Neuroimaging in Dementia | 2008 | Title and abstract clearly do not meet inclusion criteria | No |
| 1280 | Selective Motor Control is a Clinical Correlate of Brain Motor Tract Impairment in Children with Spastic Bilateral Cerebral Palsy | 2021 | Title and abstract clearly do not meet inclusion criteria | No |
| 1281 | Improved Myelination following Camp Leg Power, a Selective Motor Control Intervention for Children with Spastic Bilateral Cerebral Palsy: A Diffusion Tensor MRI Study | 2023 | Title and abstract clearly do not meet inclusion criteria | No |
| 1282 | Early prediction of unilateral cerebral palsy in infants at risk: MRI versus the hand assessment for infants | 2020 | Title and abstract clearly do not meet inclusion criteria | No |
| 1283 | Potential of diffusion tensor MR imaging in the assessment of cognitive impairments in children with periventricular leukomalacia born preterm | 2013 | review | No |
| 1284 | DTI Study of Cerebral Normal-Appearing White Matter in Hereditary Neuropathy With Liability to Pressure Palsies (HNPP) | 2015 | Title and abstract clearly do not meet inclusion criteria | No |
| 1285 | Effects of three kinds of head acupuncture therapies on regulation of brain microenvironment and rehabilitation of nerve function in rats with cerebral palsy | 2021 | Title and abstract clearly do not meet inclusion criteria | No |
| 1286 | A novel form of autosomal recessive hereditary spastic paraplegia caused by a new <i>SPG7</i> mutation | 2007 | Title and abstract clearly do not meet inclusion criteria | No |
| 1287 | Neurological manifestations of Behcet's disease | 2002 | Title and abstract clearly do not meet inclusion criteria | No |
| 1288 | A Novel Magnetic Resonance Imaging Score Predicts Neurodevelopmental Outcome After Perinatal Asphyxia and Therapeutic Hypothermia | 2018 | Title and abstract clearly do not meet inclusion criteria | No |
| 1289 | Assessment of paramedian thalamic infarcts: MR imaging, clinical features and prognosis | 2004 | Title and abstract clearly do not meet inclusion criteria | No |
| 1290 | Quantification and Monitoring of the Effect of Botulinum Toxin A on Paretic Calf Muscles of Children With Cerebral Palsy With MRI: A Preliminary Study | 2021 | Title and abstract clearly do not meet inclusion criteria | No |
| 1291 | Interhemispheric and intrahemispheric connectivity and manual skills in children with unilateral cerebral palsy | 2014 |  | Yes |
| 1292 | Understanding the relationship between brain and upper limb function in children with unilateral motor impairments: A multimodal approach | 2018 | Title and abstract clearly do not meet inclusion criteria | No |
| 1293 | Brain Plasticity following Intensive Bimanual Therapy in Children with Hemiparesis: Preliminary Evidence | 2015 |  | Yes |
| 1294 | Lack of X-linked inhibitor of apoptosis protein leads to increased apoptosis and tissue loss following neonatal brain injury | 2009 | Title and abstract clearly do not meet inclusion criteria | No |
| 1295 | Radiological Biomarkers for Diagnosis in PSP: Where Are We and Where Do We Need to Be? | 2017 | Title and abstract clearly do not meet inclusion criteria | No |
| 1296 | Imaging Measures Predict Progression in Progressive Supranuclear Palsy | 2012 | Title and abstract clearly do not meet inclusion criteria | No |
| 1297 | Alteration of Human Fetal Subplate Layer and Intermediate Zone During Normal Development on MR and Diffusion Tensor Imaging | 2010 | Title and abstract clearly do not meet inclusion criteria | No |
| 1298 | Diagnostic and therapy of salivary gland diseases | 2009 | Title and abstract clearly do not meet inclusion criteria | No |
| 1299 | Factors associated with MRI success in children cooled for neonatal encephalopathy and controls | 2023 | Title and abstract clearly do not meet inclusion criteria | No |
| 1300 | Diffusion Tensor Imaging of Parkinson's Disease, Multiple System Atrophy and Progressive Supranuclear Palsy: A Tract-Based Spatial Statistics Study | 2014 | Title and abstract clearly do not meet inclusion criteria | No |
| 1301 | The Most Cited Original Articles in Brain Imaging of Children With Cerebral Palsy: A Bibliometric Analysis Between 1984 and 2019 | 2020 | Title and abstract clearly do not meet inclusion criteria | No |
| 1302 | The plasticity of the corticospinal tract in children with obstetric brachial plexus palsy after Botulinum Toxin A treatment | 2018 | Title and abstract clearly do not meet inclusion criteria | No |
| 1303 | Radial Coherence of Diffusion Tractography in the Cerebral White Matter of the Human Fetus: Neuroanatomic Insights | 2014 | Title and abstract clearly do not meet inclusion criteria | No |
| 1304 | Preclinical chorioamnionitis dysregulates CXCL1/CXCR2 signaling throughout the placental-fetal-brain axis | 2018 | Title and abstract clearly do not meet inclusion criteria | No |
| 1305 | Distribution of the corticobulbar tract in the internal capsule | 2013 | Title and abstract clearly do not meet inclusion criteria | No |
| 1306 | Impaired glymphatic system revealed by DTI-ALPS in cerebral palsy due to periventricular leukomalacia: relation with brain lesion burden and hand dysfunction | 2023 | Non-CP | No |
| 1307 | Motor Organization in Schizencephaly: Outcomes of Transcranial Magnetic Stimulation and Diffusion Tensor Imaging of Motor Tract Projections Correlate with the Different Domains of Hand Function | 2021 | Non-CP | No |
| 1308 | Anatomical characterization of athetotic and spastic cerebral palsy using an atlas-based analysis | 2013 | No correlation analysis | No |
| 1309 | Athetotic and Spastic Cerebral Palsy: Anatomic Characterization Based on Diffusion-Tensor Imaging | 2011 | review | No |
| 1310 | Symmetrical central tegmental tract (CTT) hyperintense lesions on magnetic resonance imaging in children | 2009 | Title and abstract clearly do not meet inclusion criteria | No |
| 1311 | Quantitative diffusion tensor tractography of the motor and sensory tract in children with cerebral palsy | 2010 |  | Yes |
| 1312 | Bihemispheric alterations in myelination in children following unilateral perinatal stroke | 2018 | Duplicate Article | No |
| 1313 | Changes of White Matter Diffusion Anisotropy in Response to a 6-Week iPad Application-Based Occupational Therapy Intervention in Children with Surgically Treated Hydrocephalus: A Pilot Study | 2016 | Title and abstract clearly do not meet inclusion criteria | No |
| 1314 | Prediction of adverse outcome with cerebral lactate level and apparent diffusion coefficient in infants with perinatal asphyxia | 2002 | Title and abstract clearly do not meet inclusion criteria | No |
| 1315 | The safety and efficacy of umbilical cord blood mononuclear cells in individuals with spastic cerebral palsy: a randomized double-blind sham-controlled clinical trial | 2022 | Title and abstract clearly do not meet inclusion criteria | No |
| 1316 | Kernohan-Woltman notch phenomenon: a review article | 2017 | Title and abstract clearly do not meet inclusion criteria | No |
| 1317 | Exploration of rich-club reorganization in facial synkinesis: insights from structural and functional brain network analysis | 2023 | Title and abstract clearly do not meet inclusion criteria | No |
| 1318 | Multivariate Analysis and Machine Learning in Cerebral Palsy Research | 2017 | Title and abstract clearly do not meet inclusion criteria | No |
| 1319 | DTI Tract-Based Quantitative Susceptibility Mapping: An Initial Feasibility Study to Investigate the Potential Role of Myelination in Brain Connectivity Change in Cerebral Palsy Patients During Autologous Cord Blood Cell Therapy Using a Rotationally-Invariant Quantitative Measure | 2021 | Title and abstract clearly do not meet inclusion criteria | No |
| 1320 | Changes of Structural Brain Network Following Repetitive Transcranial Magnetic Stimulation in Children With Bilateral Spastic Cerebral Palsy: A Diffusion Tensor Imaging Study | 2021 | No correlation analysis | No |
| 1321 | Clinical observation on scalp acupuncture for diffusion tensor imaging in cerebral palsy children with periventricular leukomalacia | 2017 | No correlation analysis | No |
| 1322 | Similarities and differences between infantile and early childhood onset vanishing white matter disease | 2018 | Title and abstract clearly do not meet inclusion criteria | No |
| 1323 | Score for Neonatal Acute Physiology-II and Neonatal Pain Predict Corticospinal Tract Development in Premature Newborns | 2013 | Title and abstract clearly do not meet inclusion criteria | No |
| From other resources | | | | |
| 1324 | Altered microstructural connectivity of the superior and middle cerebellar peduncles are related to motor dysfunction in children with diffuse periventricular leucomalacia born preterm: a DTI tractography study | 2014 |  | Yes |
| 1325 | Arcuate fasciculus in young people with severe cerebral palsy: A diffusion tensor imaging study | 2011 | Duplicate Article | No |
| 1326 | Relation between brain lesions on MRI and gait pathology in children with cerebral palsy | 2013 | Duplicate Article | No |
| 1327 | Athetotic and Spastic Cerebral Palsy: Anatomic Characterization Based on Diffusion-Tensor Imaging | 2011 | review | No |
| 1328 | Capturing neuroplastic changes after bimanual intensive rehabilitation in children with unilateral spastic cerebral palsy: A combined DTI, TMS and fMRI pilot study | 2015 | No correlation analysis | No |
| 1329 | Diffusion tensor imaging demonstrates focal lesions of the corticospinal tract in hemiparetic patients with cerebral palsy | 2007 | No correlation analysis | No |
| 1330 | Impact of Lower Limb Active Movement Training in Individuals With Spastic Type Cerebral Palsy on Neuromuscular Control Outcomes: A Systematic Review | 2020 | review | No |
| 1331 | Activation of less affected corticospinal tract and poor motor outcome in hemiplegic pediatric patients: a diffusion tensor tractography imaging study | 2015 | Duplicate Article | No |
