# Supplementary material for: White matter lesions and DTI metrics related to various types of dysfunction in cerebral palsy: A meta-analysis and systematic review
Source: PLoS One. 2025 Jan 24;20(1):e0312378. doi: 10.1371/journal.pone.0312378 (PMC11760009; doi:10.1371/journal.pone.0312378)
Supplement: Attachment — Submitted filename: Response to Reviewers.docx [file pone.0312378.s011.docx]

Please use the space provided to explain your answers to the questions above. You may also include additional comments for the author, including concerns about dual publication, research ethics, or publication ethics. (Please upload your review as an attachment if it exceeds 20,000 characters)

Reviewer #1: The meta-analysis and review manuscript by Jiang et al. assesses the correlation between various types of disfunction and DTI metrics in cerebral palsy. The manuscript is well written and it is technically sound. There are only a few minor modifications required:

1. There are some instances that the full version of the abbreviations are not mentioned in the text or table (e.g., GPS, ADC, etc.). In addition, there are some discrepancies in the abbreviations and the metric names. For instance, AFD is the abbreviation for 'apparent fiber density'; whereas it is noted as axial diffusivity in the text. You can confirm it with the reference (ref. 20). I suggest adding a table explaining the abbreviations for scales, pathways and DTI metrics and proofreading all the shortened forms in the text and table.

**Dear Reviewer,I have proofread and revised the abbreviations.** **Here is the table I have compiled.**

| Abbreviation | |
| --- | --- |
| Cerebral Palsy | CP |
| unilateral cerebral palsy | UCP |
| Magnetic Resonance Imaging | MRI |
| Diffusion Tensor Imaging | DTI |
| Diffusion Tensor Image Analysis along the Perivascular Space | DTI-ALPS |
| White Matter | WM |
| Fractional Anisotropy | FA |
| Mean Diffusivity | MD |
| Radial Diffusivity | RD |
| Average Diffusion Coefficient | ADC |
| Fiber Number | FN |
| Apparent Fiber Density | AFD |
| Asymmetry Index | AI |
| Anterior Cingulate Cortex | ACC |
| Corticospinal Tract | CST |
| Posterior Limb of the Internal Capsule | PLIC |
| Posterior Thalamic Radiation | PTR |
| Corpus Callosum | CC |
| Optic Radiation | OR |
| Superior Longitudinal Fasciculus | SLF |
| Periventricular Leukomalacia | PVL |
| Parietal White Matter | PWM |
| Frontal White Matter | FWM |
| Occipital White Matter | OWM |
| Temporal White Matter | TWM |
| Melbourne assess-ment 2 | MA2 |
| Gross Motor Function Classification System | GMFCS |
| Gross Motor Function Measure | GMFM |
| Box and Block Test | BBT |
| Assisting Hand Assessment | AHA |
| Manual Ability Classification System | MACS |
| Jebsen-Taylor Hand Function Test | JTHFT |
| Children's Hand-use Experience Questionnaire | CHEQ |
| Selective Control Assessment of the Lower Extremity | SCALE |
| Time Up and Go Test | TUG |
| Dysphagia Disorder Survey | DDS |
| Peabody Picture Vocabulary Test | PPVT |
| Wisconsin Card Sorting Test | WCST |
| Global Pain Scale | GPS |
| three-dimensional instrumented gait analysis | 3DGA |
| 10 Meter Walk Test | 10MWT |
| Functional Level of Hemiplegia | FxL |
| Revisie Amsterdamse Kinder Intelligentie Test | RAKIT |

2. There are several typos in table 1. For example:

- 'country' in the first row is not spelled correctly

- non-English font in DTI metrics

- countries 'Spanish', 'Indian', and 'Iranian' should change to 'Spain', 'India', and 'Iran'.

**Thank you for pointing that out. I have made the corrections in the manuscript.**

3. There are studies in the field from established research groups which are not cited in the manuscript. For example, the DTI studies of Papadelis group have been omitted from this review paper.

**I have added the citation, such as the third reference, which is a study by the Papadelis group.**

Reviewer #2: The article has some novelty and positive results. Overall the methodology is sound and the conclusions are supported by the results. However, the details of this paper should be improved. See below for some comments in the different sections of the paper:

1.Abstract：The relatively r values,95% CIs and I² value should be given in main results. The parts of results section seems a little simple.

**Dear Reviewer,I have added to the results section of the abstract.** **Marked in red were the added sections.**

**“Results：46 studies involving 1458 children with cerebral palsy, were included. 19 articles for Meta-analysis and 27 articles were descriptively analyzed. DTI metrics such as FA、MD in both sensory and motor pathways significantly correlated with various function ratings. In overall motor dysfunction, compared with the CST and PTR, FA of the PLIC correlated more strongly with GMFCS ,and showed no significant heterogeneity (r=-1.28, confidence interval [CI]-1.70 to -0.87,I2=38.2%,P=0.233). In upper limb dysfunction, compared with the AHA and MA2, FA of the CST correlated more strongly with BBT,and showed no significant heterogeneity (r=-0.56, confidence interval [CI]-0.78 to -0.34,I2=0.0%,P =0.511). Lower limb dysfunction and other dysfunctions we used qualitative analysis.The qualitative analysis offered a concise overview of each investigation.”**

2.Introduction：

（1）Consider more about the primary and secondary objective of this systematic review and emphasize it.

**I have distinguished between the primary and secondary objectives.**

**In the last paragraph of the introduction:“Thus, this work examined the relationship between DTI metrics of sensorimotor pathways and different dysfunction scores using meta-analysis and systematic review. Our primary objective was to identify the specific white matter lesions and DTI metrics linked to various types of impairment in individuals with cerebral palsy. Our secondary objective was to summarize commonly used scales for assessing various types of functional impairment in cerebral palsy clinics”**

（2）When mentioned DTI indexes and metric analysis,all the recommended names and clinical significance in the following paper should be involved,for example ADC is missed.

**I have made the additions.**

**In the second paragraph of the introduction:”** **DTI primarily utilizes Fractional Anisotropy (FA) as a metric to measure the anisotropy of water diffusion in tissues. FA can provide insights into the connectivity of nerve fiber bundles in various brain areas. Mean Diffusivity (MD) is a frequently utilized DTI metric that measures of intra- and extra-cellular water diffusion[3]. It is used to evaluate the integrity of brain tissue. The main function of the apparent diffusion coefficient (ADC) value is to quantitatively analyse the dispersion degree of molecules, and this value represents the diffusion efficiency of water molecules in tissues.Axial Diffusivity (AD),Radial Diffusivity (RD) and apparent fiber density（AFD）can be more specific about the direction and size of water molecule diffusion in tissues. They are reliable pathologic features of cerebral palsy. DTI metric analysis can provide insights into the extent of brain injury, assess the impact of rehabilitation therapy, and inform the development of treatment strategies[4, 5].”**

3.Methods：

（1）The search term strategy was not comprehensive (cerebral palsy was used only in cerebral palsy, and no other free words were used).Please improve your search strategy.

**I have improved the search strategy.**

**The search terms we used were "cerebral palsy", "CP", "congenital cerebral palsy" , "monoplegic cerebral palsy"or " quadriplegic infantile cerebral palsy and "diffusion magnetic resonance imaging", "diffusion MRI", "diffusion tensor imaging","DTI"or "tractography."**

（2）Bias was not assessed.Please provide a funnel plot for publication bias in Appendix.

Funnel plot of publication bias for studies on the correlation between FA in CST and GMFCS.

Funnel plot of publication bias for studies on the correlation between FA in PTR and GMFCS.

Funnel plot of publication bias for studies on the correlation between FA in PLIC and GMFCS.

Funnel plot of publication bias for studies on the correlation between FA in CST and BBT.

Funnel plot of publication bias for studies on the correlation between FA in CST and MA2.

Funnel plot of publication bias for studies on the correlation between FA in CST and AHA.

（3）A fixed-effect model should be taken when I2 < 50%，it is not mentioned in this article.

**I have added.**

**Meta-analysis and systematic review**

**In this meta-analysis, the r values for the correlation between DTI metrics within different sensorimotor pathways (CST、PTR、PLIC，etc) and the function scores were pooled respectively. The r values were extracted for each study, and the extent of r was quantitatively explored. We converted Spearman correlation coefficients to Pearson correlation coefficients.Data heterogeneity was analyzed by using the inconsistency index (I2) value. I2 >50% indicated high heterogeneity and a random effects model was used to analyze combined data from the selected studies. A fixed-effect model should be taken when I2 < 50%.All analyses were performed using the software Stata, version 14. P<0.05 was considered statistically significant. We conducted a qualitative descriptive report for studies where meta-analysis could not be performed.**

4.Results：

（1）Please list the NOS evaluation score form of all studies.

（2）Upper limb motor function disorder：After pooling 3 studies[9, 10, 22], the FA in CST correlated with MA2 (r =-0.43,95% confidence interval [CI] –1.33 to 0.46) and was markedly heterogeneous （I2=95.1%，P=0.000）. The 95% confidence interval [CI] contains 0,so it's not correlated between FA in CST and MA2.

（3）Upper limb motor function disorder：After pooling 8 studies[9, 22-28], the FA in CST correlated with Assisting Hand Assessment（AHA） (r =0.46,95% confidence interval [CI] 0.32 to 0.60) and was markedly heterogeneous （I2=14.5%，P=0.317）. I2＜50%，P＞0.05，it is not markedly heterogeneous.

**Thank you to the reviewer for pointing out the errors; I have made the corrections.**

**“upper limb motor function disorder**

**A total of 12 studies conducted meta-analyses, all studied the correlation between FA in CST and upper limb motor function, and the summarized r values were shown in Figure 3.After pooling 3 studies[11, 21, 22], the FA in CST correlated with Box and Block Test （BBT） (r =-0.56,95% confidence interval [CI] –0.78 to –0.34) and was not markedly heterogeneous(I2=0.0%，P =0.511）. After pooling 3 studies[10, 11, 23], the FA in CST uncorrelated with MA2 (r =-0.43,95% confidence interval [CI] –1.33 to 0.46). After pooling 8 studies[10, 23-29], the FA in CST correlated with Assisting Hand Assessment（AHA） (r =0.46,95% confidence interval [CI] 0.32 to 0.60) and was not markedly heterogeneous （I2=14.5%，P=0.317）.“**

**Thank you to the reviewer for pointing out the errors; I have made the corrections.**

5.Discussion：No comments.

**I have revised the discussion section.** **In the first and second paragraphs of the discussion, the added sections are marked in red. “The results of this study suggest that the combined effects of damage in multiple white matter regions may lead to motor impairments in patients with cerebral palsy.Through systematic review and evaluation, it has been found that in cerebral palsy, various sensory-motor pathways such as CST, PTR, and PLIC's FA were significantly correlated with overall motor function scores, such as the GMFCS score, without significant heterogeneity observed. Among these, the lesions in the PLIC were most closely associated with overall motor dysfunction. Additionally, other regions of the brain such as the CC, cingulum, and superior and inferior longitudinal fasciculi also showed correlations with GMFCS, although they were not included in the meta-analysis. Clinically, GMFCS is a set of objective evaluation system designed for comprehensive assessment of motor function in cerebral palsy patients, and it is the most used classification system, ranging from level I to V, where higher levels indicate poorer functional abilities. In DTI, FA is a commonly used objective indicator for assessing the extent of brain white matter damage. Previous research has predominantly focused on the association between motor impairments in spastic cerebral palsy and reduced FA values. However, cerebral palsy includes various types, such as mixed cerebral palsy and dyskinetic cerebral palsy. This study comprehensively included all types of cerebral palsy during the search and still found the same conclusion. Therefore, FA can be considered as one of the indicators for predicting motor impairments in patients with cerebral palsy. In DTI, ADC can be used to quantitatively assess the directionality and extent of water molecule movement in bodily tissues and has been found to be related to GMFCS. However, currently, there is only one study on the predictive value of ADC, and further development in this area is needed.**

**Upper limb movements are finer and more complex, and there are also many scales available for assessing upper limb movements. The results of this study showed that upper limb motor impairments were correlated with damage to the CST and had the highest correlation with the BBT score, but the most studies included in the research applied the AHA to assess upper limb function. The BBT is a simple test method for assessing hand function flexibility, while the AHA is commonly used clinically to assess fine upper limb activities. This study found that the FA in the CST is not correlated with MA2. MA2 is a comprehensive scale for assessing upper limb function in children with neurological disorders. This result is contrary to previous studies, and therefore, increasing the sample size for further investigation is needed.Therefore, there is still a need in clinical practice to further explore comprehensive scales for evaluating upper limb function in cerebral palsy, covering various aspects of upper limb movement function as much as possible.“**

6.Overall:General writing quality should be ameliorated.

**Thank you to the reviewer for pointing out the shortcomings.**
