# Supplementary material for: White matter lesions and DTI metrics related to various types of dysfunction in cerebral palsy: A meta-analysis and systematic review
Source: PLoS One. 2025 Jan 24;20(1):e0312378. doi: 10.1371/journal.pone.0312378 (PMC11760009; doi:10.1371/journal.pone.0312378)
Supplement: Attachment — Submitted filename: Response to Reviewers.docx [file pone.0312378.s012.docx]

**Response to Reviewers**

Reviewer #1: (No Response)

Reviewer #2: Thanks for your specific revision.Now there is still a little minor details should be revised:

（1）A minor error exists in part“Literature Search”：After removing 391 duplicate articles, the remaining 940（not 930） articles were screened.

**Thank you for pointing out the error carefully. I have already corrected it.**

（2）Table 1：Adjust the form to three-wire watch and add the corresponding NOS score in the last column.

**I have adjusted the table format according to your suggestion and added a column for the NOS score.**

（3）After adding the corresponding NOS score to table 1，table S2（the NOS Evaluation Score Form of All Studies）and table S1（Abbreviation）in appendix can choosed to be deleted,else there are too many tables.（or you can discuss it with editor eventually）

**I have deleted table S2 according to your suggestion, but I have temporarily kept table S1.**

**Additionally, I would like to thank the reviewers and editors for their efforts. There were fewer revision suggestions this time, so the revision process was quick. I also highly value this paper, so I resubmitted it as soon as possible.**
